# ﻿The bees of the genus *Andrena* Fabricius, 1775 (Hymenoptera, Andrenidae) described by Ferdinand Morawitz from the collection of Aleksey Fedtschenko

**DOI:** 10.3897/zookeys.1120.90206

**Published:** 2022-09-05

**Authors:** Yulia V. Astafurova, Maxim Yu. Proshchalykin, Dmitry A. Sidorov

**Affiliations:** 1 Zoological Institute, Russian Academy of Sciences, Universitetskaya Nab., 1, Saint Petersburg, 199034, Russia Zoological Institute, Russian Academy of Sciences Saint Petersburg Russia; 2 Federal Scientific Center of the East Asia Terrestrial Biodiversity, Far Eastern Branch of Russian Academy of Sciences, Vladivostok-22, 690022, Russia Federal Scientific Center of the East Asia Terrestrial Biodiversity, Far Eastern Branch of Russian Academy of Sciences Vladivostok Russia; 3 Kemerovo State University, Kemerovo, 650000, Russia Kemerovo State University Kemerovo Russia

**Keywords:** Anthophila, Apiformes, lectotypes, Palaearctic Region, taxonomy

## Abstract

The type specimens of the genus *Andrena* Fabricius, 1775, described by Ferdinand Morawitz from the collection of Aleksey Fedtschenko deposited in the Zoological Museum of the Moscow State University and in the Zoological Institute, Russian Academy of Sciences, St. Petersburg (Russia), are critically reviewed. Precise information with illustrations of types for 52 taxa is provided; of these 39 species are valid and thirteen are invalid (ten synonyms and three homonyms). Lectotypes are here designated for the following 24 nominal taxa: *Andrenaacutilabris* Morawitz, 1876, *A.aulica* Morawitz, 1876, *A.bicarinata* Morawitz, 1876, *A.combusta* Morawitz, 1876, *A.comparata* Morawitz, 1876, *A.corallina* Morawitz, 1876, *A.discophora* Morawitz, 1876, *A.fedtschenkoi* Morawitz, 1876, *A.initialis* Morawitz, 1876, *A.laeviventris* Morawitz, 1876, *A.leucorhina* Morawitz, 1876, *A.mucorea* Morawitz, 1876, *A.nitidicollis* Morawitz, 1876, *A.oralis* Morawitz, 1876, *A.planirostris* Morawitz, 1876, *A.ravicollis* Morawitz, 1876, *A.rufilabris* Morawitz, 1876, *A.sarta* Morawitz, 1876, *A.smaragdina* Morawitz, 1876, *A.sogdiana* Morawitz, 1876, *A.subaenescens* Morawitz, 1876, *A.tuberculiventris* Morawitz, 1876, *A.turkestanica* Morawitz, 1876, and *A.virescens* Morawitz, 1876.

## ﻿Introduction

The present paper is the second part of a series of works dealing with the bee taxa described by Ferdinand Morawitz from the collection of Aleksey Fedtschenko deposited in the Zoological Museum of the Moscow State University, Moscow (ZMMU) and in the Zoological Institute of the Russian Academy of Sciences, St. Petersburg (ZISP). In the first part, the family Halictidae was considered (Astafurova and Proshchalykin 2020). The main goal of this work is to make Fedtschenko’s collection of bees more accessible and useful to scientists.

More than 140 years ago (1876), the second part of Ferdinand Morawitz’s critical study on the bees collected by Aleksey Fedtschenko 1869–1871 Expeditions in “Turkestan” was published. In the first volume, “Apidae genuinae” (Morawitz 1875), Morawitz treated a total of 255 species of numerous genera, of which many species were described as new. In this second part, “Andrenidae” (Morawitz 1876), the remaining bees were dealt with, including the species of the difficult genera *Andrena* Fabricius, *Halictus* Latreille, and *Hylaeus* Fabricius, totalling 183 species (Pesenko and Astafurova 2003). The species treatments are of a high professional standard, the localities are precisely documented (A. Fedtschenko 1871; O. Fedtschenko 1874; Baker 2004; Kuhlmann 2005; Dathe and Proshchalykin 2017) and the type series have been carefully conserved over a long period, generally in the collections of the ZMMU and ZISP. To this day, these remain some of the most important manuscripts on bees of Central Asia.

*Andrena* Fabricius, 1775 is the one of the largest bee genera, numbering about 1550 species worldwide, of which about 950 are known from the Palaearctic region (Gusenleitner and Schwarz 2002; Ascher and Pickering 2022). The genus is distributed throughout the Holarctic region, south in the Western Hemisphere to Panama, in Africa through the East African highlands and south to the Cape of Good Hope, and in Asia to the mountains of southern India and of Malaysia (Michener 2007). Only three species of *Andrena* are distributed in both the Palearctic and the Holarctic regions, whereas the others occur in only one of these zoogeographic realms. Many species appear to be widespread, and many are bivoltine, which has led to separate descriptions in different regions, especially in the Palearctic. Thus, about 3000 species descriptions were recognised at the beginning of 21^st^ century (Gusenleitner and Schwarz 2002).

The genus *Andrena* is represented in Morawitz’s 1876 publication by 68 species (Nos. 258–326). Only 16 species were previously known, while the remaining 52 were newly described (Table 1). Few of these taxa have been mentioned in subsequent publications, remaining enigmatic for decades.

**Table 1.** The nominal taxa of Fedtschenko’s *Andrena*, described by F. Morawitz, and their current status.

N	Species name	Sex	Current status	Depositaries of types
1.	*Andrenaacutilabris*	♀, ♂	Valid	LT (ZISP); PLT (ZISP/ZMMU)
2.	*Andrenaahenea*	♀, ♂	Valid	LT, PLT (ZMMU)
3.	*Andrenaamoena*	♀	Valid	LT, PLT (ZMMU)
4.	*Andrenaarenaria*	♂	Synonym	HT (ZMMU)
5.	*Andrenaaulica*	♀, ♂	Valid	LT (ZISP); PLT (ZISP/ZMMU)
6.	*Andrenabairacumensis*	♀	Valid	HT (ZMMU)
7.	*Andrenabicarinata*	♀	Synonym	LT (ZISP); PLT (ZISP/ZMMU)
8.	*Andrenacapillosa*	♀	Valid	LT (ZMMU); PLT (ZISP)
9.	*Andrenacarinifrons*	♀	Valid	LT (ZISP)
10.	*Andrenacombusta*	♀, ♂	Valid	LT, PLT (ZMMU)
11.	*Andrenacomparata*	♂	Synonym	LT (ZISP); PLT (ZISP/ZMMU)
12.	*Andrenacorallina*	♀	Synonym	LT (ZMMU)
13.	*Andrenadiscophora*	♀, ♂	Valid	LT (ZMMU); PLT (ZISP/ZMMU)
14.	*Andrenafedtschenkoi*	♀, ♂	Valid	LT (ZISP); PLT (ZISP/ZMMU)
15.	*Andrenaferghanica*	♀	Valid	HT (ZISP)
16.	*Andrenaflavitarsis*	♀, ♂	Valid	LT (ZMMU); PLT (ZISP/ZMMU)
17.	*Andrenafuscicollis*	♀, ♂	Valid	LT (ZMMU); PLT (ZISP/ZMMU)
18.	*Andrenahieroglyphica*	♀	Valid	HT (ZMMU)
19.	*Andrenainfirma*	♀, ♂	Valid	LT (ZMMU); PLT (ZISP/ZMMU)
20.	*Andrenainitialis*	♀, ♂	Valid	LT (ZISP); PLT (ZISP/ZMMU)
21.	*Andrenalaeviventris*	♀	Valid	LT, PLT (ZMMU)
22.	*Andrenalateralis*	♀, ♂	Valid	LT (ZMMU); PLT (ZISP/ZMMU)
23.	*Andrenaleucorhina*	♂	Synonym	LT (ZMMU); PLT (ZISP/ZMMU)
24.	*Andrenalucidicollis*	♂	Valid	ST (ZMMU)
25.	*Andrenamaculipes*	♀, ♂	Valid	LT (ZISP); PLT (ZISP/ZMMU)
26.	*Andrenamajalis*	♀	Valid	LT (ZMMU); PLT (ZISP/ZMMU)
27.	*Andrenamordax*	♂	Valid	LT (ZMMU); PLT (ZISP/ZMMU)
28.	*Andrenamucorea*	♀, ♂	Valid	LT (ZMMU); PLT (ZISP/ZMMU)
29.	*Andrenanigrita*	♂	Homonym	HT (ZMMU)
30.	*Andrenanitidicollis*	♀, ♂	Valid	LT, PLT (ZMMU)
31.	*Andrenanupta*	♀, ♂	Valid	LT (ZMMU); PLT (ZISP/ZMMU)
32.	*Andrenaoralis*	♀	Valid	LT (ZISP); PLT (ZMMU)
33.	*Andrenapannosa*	♀	Valid	LT (ZISP); PLT (ZISP/ZMMU)
34.	*Andrenaplanirostris*	♀, ♂	Valid	LT (ZISP); PLT (ZISP/ZMMU)
35.	*Andrenapunctifrons*	♀	Valid	HT (ZMMU)
36.	*Andrenapunctiventris*	♀	Valid	HT (ZMMU)
37.	*Andrenaquadrifasciata*	♀	Valid	HT (ZMMU)
38.	*Andrenaravicollis*	♀	Synonym	LT (ZISP); PLT (ZMMU)
39.	*Andrenarufilabris*	♀	Synonym	LT, PLT (ZMMU)
40.	*Andrenarufina*	♀, ♂	Valid	LT, PLT (ZMMU)
41.	*Andrenasarta*	♀	Valid	LT (ZMMU)
42.	*Andrenasemiaenea*	♀	Valid	LT (ZMMU); PLT (ZISP/ZMMU)
43.	*Andrenasmaragdina*	♀, ♂	Valid	LT (ZISP); PLT (ZMMU)
44.	*Andrenasogdiana*	♂	Synonym	LT (ZISP)
45.	*Andrenasordida*	♀, ♂	Homonym	LT (ZMMU); PLT (ZISP/ZMMU)
46.	*Andrenasubaenescens*	♀, ♂	Valid	LT (ZISP); PLT (ZISP/ZMMU)
47.	*Andrenatemporalis*	♂	Homonym	LT (ZMMU); PLT (ZISP/ZMMU)
48.	*Andrenatuberculiventris*	♂	Valid	LT (ZMMU); PLT (ZISP/ZMMU)
49.	*Andrenaturkestanica*	♀, ♂	Synonym	LT (ZISP); PLT (ZMMU)
50.	*Andrenaurmitana*	♂	Synonym	HT (ZMMU)
51.	*Andrenavirescens*	♀, ♂	Valid	LT (ZISP); PLT (ZISP/ZMMU)
52.	*Andrenaviridigastra*	♀, ♂	Valid	LT (ZMMU); PLT (ZISP/ZMMU)

LT – lectotype, PLT – paralectotype/s, ST – syntype.

Since the 1940s, V. Popov [ZISP] based his studies on the taxonomy and ecology of the Central Asian *Andrena* fauna using these collections (Popov 1940, 1947, 1949, 1952, 1958, 1960).

Type material of Fedtschenko’s *Andrena* was studied by K. Warncke, a teacher in Dachau (Germany), who visited the ZMMU from 26.03.1975 to 01.04.1975 (Dathe and Proshchalykin 2017). He worked his way through the drawers containing the Fedtschenko material and labelled specimens of all nominal taxa found there as “Lectotypus.” Thereby, certain specimens from some groups of bees (*Hylaeus*, *Andrena*, *Anthidium*, *Halictus*, etc.) received a red label with the inscription “Lectotypus Warncke 1975.” All other type specimens were left without nomenclatural status labels. However, unlike other groups of bees (Warncke 1981, 1982), he did not publish information about the designation of *Andrena* lectotypes.

In 1980 A.Z. Osytshnjuk continued the study of *Andrena* in the Fedtschenko’s collection of the ZISP and ZMMU. The lectotype and paralectotype designations by A. Osytshnjuk were largely unpublished, because of her tragic death in 1998. Some of these designations have been posthumously validated in subsequent publications (Osytshnjuk et al. 2005, 2008).

The present paper is the first complete, critically reviewed, illustrated summary of all species of the genus *Andrena* described by F. Morawitz from the collection of A. Fedtschenko, an invaluable reference for researchers across this region who otherwise could not easily assign names to these difficult bees.

## ﻿Materials and methods

All of the material listed below was examined for this study. In the following list, the taxa are treated in alphabetical order of the names used in the original descriptions. Each entry includes the name of the taxon in its original combination, the complete reference to the original description of the species (including the original combination and spelling of the name and the author, year, and page of the description) and a list of type specimens present in the collections of the Zoological Museum of the Moscow State University, Moscow (**ZMMU**) and Zoological Institute of the Russian Academy of Sciences, St. Petersburg (**ZISP**). The data from each label are separated by two slashes (//). Square brackets are used for English translations and when information is added to specimen label information (e.g., geographical coordinates) or published data (e.g., current name of an old place name; affiliation to a present-day country).

Photographs were made using a combination of a stereomicroscope Olympus SZX10 and a digital camera (Olympus OM-D). Illustrations were obtained by montaging from an image series that covers different focal planes into a single in-focus image with the program Helicon Focus 7. The final illustrations were post-processed for contrast and brightness using Adobe Photoshop software.

The classification and current species status mostly follow Gusenleitner and Schwarz (2002) and Pisanty et al. (2022), distribution follows Osytshnjuk et al. (2005, 2008), Proshchalykin et al. (2017), Wood and Monfared (2022), and Ascher and Pickering (2022). In the dating the publications of Morawitz’s paper, we follow Kerzhner (1984) and Ebmer (2021).

## ﻿Taxonomy

### ﻿List of species

**Genus *Andrena* Fabricius, 1775**

#### ﻿1. Andrena acutilabris

Taxon classificationAnimaliaHymenopteraAndrenidae

Morawitz, 1876

9B7E5D93-5C81-551C-BEC4-7012E1FA0BC7

Fig. 1

Andrena acutilabris Morawitz, 1876: 165 (key), 175, ♀, ♂.

##### Type locality.

Tashkent (Uzbekistan).

##### Published (original) locality.

Uzbekistan: Tashkent, Urmitan.

##### Lectotype (designated here).

♀, Ташкентъ [Uzbekistan, Tashkent, 41°18'N, 69°16'E] // к.[оллекция] Ф. Моравица [Collection of F. Morawitz] // *acutilabris* [handwritten by F. Morawitz] // Paralectotypus *Andr.acutilabris* Mor., design. Osychnjuk, 1980 <red label> // Lectotypus *Andrenaacutilabris* Morawitz, 1876, design. Astafurova et al., 2022 <red label> // Zoological Institute St. Petersburg INS_HYM_0000300 [ZISP].

**Figure 1.**
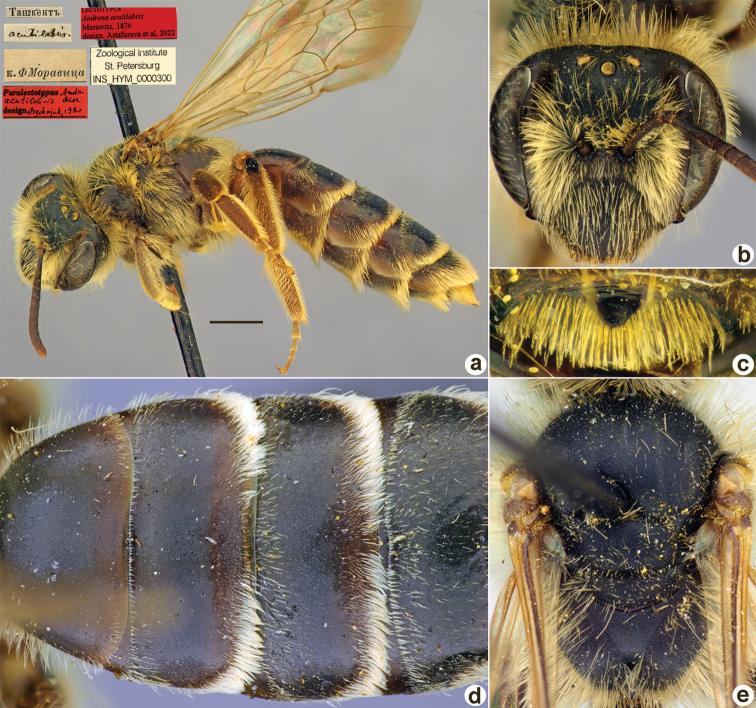
*Andrenaacutilabris* Morawitz, 1876, lectotype, female **A** habitus, lateral view and labels **B** head, frontal view **C** labrum, dorsal view **D** T1–T4, dorsal view **E** mesosoma, dorsal view. Scale bar: 1.0 mm.

##### Paralectotypes

**(3 ♀, 10 ♂).** 2 ♀, 4 ♂, 2, 3, 8, 11[IV.1871] // Ташкентъ // к.[оллекция] Ф. Моравица [Collection of F. Morawitz] // *acutilabris* Mor. [handwritten by F. Morawitz] // Paralectotypus *Andrenaacutilabris* Mor., design. Osychnjuk, 1980 <red label> [ZISP]; 6 ♂, 2., 5.[IV.1871] // Ташкентъ; 1 ♀, 11.[IV.1871] // Ташкентъ // Lectotypus Warncke 1975 // Paralectotypus *Andrenaacutilabris* Morawitz, 1876, design. Astafurova et al., 2022 <identical red labels on each paralectotype specimen> [ZMMU].

##### Current status.

Andrena (Nobandrena) acutilabris Morawitz, 1876.

##### Distribution.

Turkmenistan, Uzbekistan, Tajikistan, Kazakhstan.

#### ﻿2. Andrena ahenea

Taxon classificationAnimaliaHymenopteraAndrenidae

Morawitz, 1876

4EB0DE5A-2A36-5D91-BFE3-B0E72FF30969

Fig. 2

Andrena ahenea Morawitz, 1876: 164, 166 (key), 210, ♀, ♂.

##### Type locality.

Samarkand (Uzbekistan).

##### Published (original) locality.

Uzbekistan: Samarkand.

##### Lectotype.

♀, designated by Osychnjuk et al. 2008: 51, 16.[III.1869] // Самаркандъ [Uzbekistan, Samarkand, 39°39'N, 66°57'E] // *Andrenaahenea* Mor. [handwritten by F. Morawitz] // Lectotypus Warncke 1975 <red label> // Lectotypus *Andrenaahenea* Morawitz, 1876, design. Osytshnjuk et al., 2008 <red label, labelled by Yu. Astafurova> [ZMMU].

**Figure 2.**
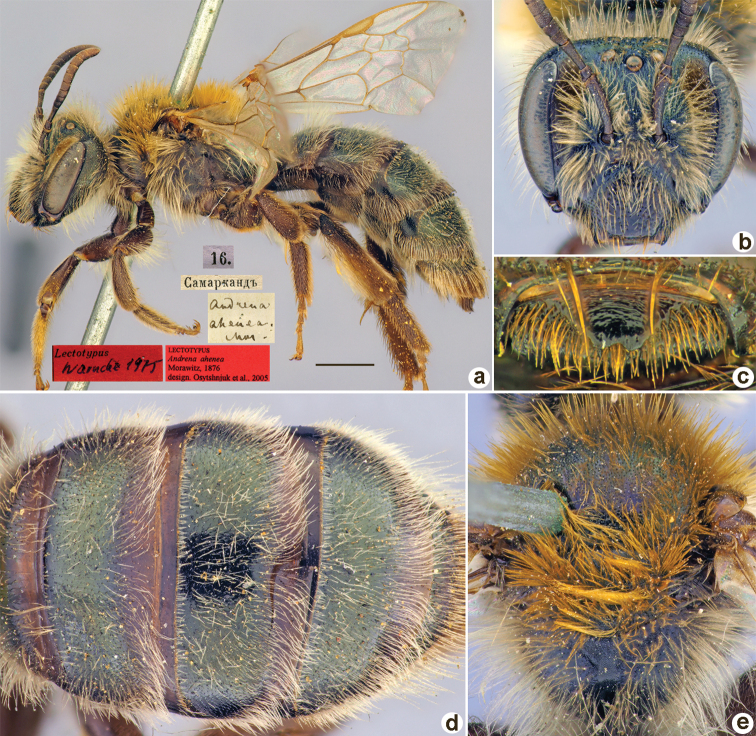
*Andrenaahenea* Morawitz, 1876, lectotype, female **A** habitus, lateral view and labels **B** head, frontal view **C** labrum, dorsal view **D** metasoma, dorsal view **E** mesosoma, dorsal view. Scale bar: 1.0 mm.

##### Paralectotype.

1 ♂, 16.[III.1876] // Самаркандъ // Paralectotypus *Andrenaahenea* Morawitz, 1876, design. Osytshnjuk et al., 2008 <red label, labelled by Yu.Astafurova> [ZMMU].

##### Current status.

Andrena (Euandrena) ahenea Morawitz, 1876.

##### Distribution.

Uzbekistan.

#### ﻿3. Andrena amoena

Taxon classificationAnimaliaHymenopteraAndrenidae

Morawitz, 1876

C47DE2A4-D7F6-5EA9-AF77-05732ADA4358

Fig. 3

Andrena amoena Morawitz, 1876: 164 (key), 211, ♀.

##### Type locality.

Chardara (Kazakhstan).

##### Published (original) locality.

Kazakhstan: Syr Darja River near Chardara.

##### Lectotype.

♀, designated by Osytshnjuk et al. 2005: 67, 25.[IV.1871] // Чардара [Kazakhstan, Chardara, 41°18'N, 67°57'E] // *Andrenaamoena* Mor. [handwritten by F. Morawitz] // Lectotypus Warncke 1975 <red label> // Lectotypus *Andrenaamoena* Morawitz, 1876, design. Osytshnjuk et al., 2005 <red label, labelled by Yu. Astafurova> [ZMMU].

**Figure 3.**
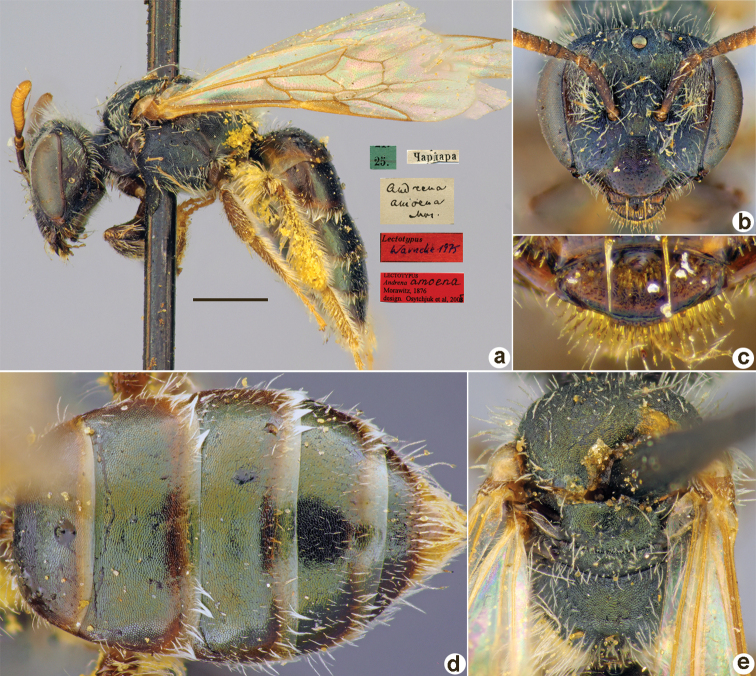
*Andrenaamoena* Morawitz, 1876, lectotype, female **A** habitus, lateral view and labels **B** head, frontal view **C** labrum, dorsal view **D** metasoma, dorsal view **E** mesosoma, dorsal view. Scale bar: 1.0 mm.

##### Paralectotypes

**(3 ♀).** 3 ♀, 25.[IV.1871] // Чардара [Chardara] // Paralectotypus *Andrenaamoena* Morawitz, 1876, design. Osytshnjuk et al., 2005 <red label, labelled by Yu. Astafurova> [ZMMU].

##### Current status.

Andrena (Aciandrena) amoena Morawitz, 1876 (according to Osytshnjuk et al. 2005: 67).

##### Remarks.

Description of male: Gusenleitner and Schwarz 2001: 99, fig. 1.

##### Distribution.

Turkmenistan, Uzbekistan, Tajikistan.

#### ﻿4. Andrena arenaria

Taxon classificationAnimaliaHymenopteraAndrenidae

Morawitz, 1876

2F76774E-CF43-5C40-B7A3-D8059933E633

Fig. 4

Andrena arenaria Morawitz, 1876: 201, ♂.

##### Type locality.

Central Asia.

##### Published (original) locality.

“Turkestan”.

##### Holotype.

♂, 487. // Туркест.[анский] кр.[ай] [Turkestan] // *Andrenaarenaria* Mor. [handwritten by F. Morawitz] // Lectotypus Warncke 1975 <red label> // Holotypus *Andrenaarenaria* Morawitz, 1876 <red label, labelled by Yu. Astafurova> // *Andrenalateralis*, D.A. Sidorov det. 2022 [ZMMU].

**Figure 4.**
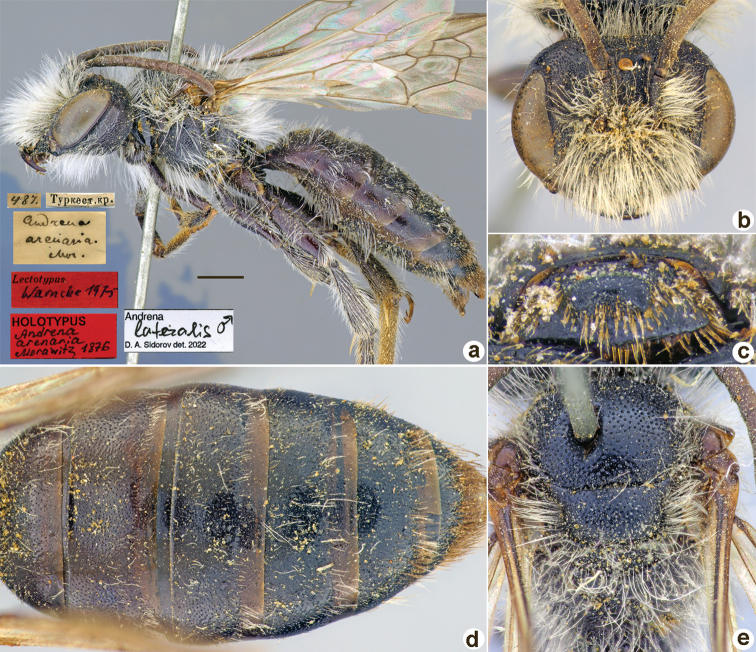
*Andrenaarenaria* Morawitz, 1876, holotype, male **A** habitus, lateral view and labels **B** head, frontal view **C** labrum, dorsal view **D** metasoma, dorsal view **E** mesosoma, dorsal view. Scale bar: 1.0 mm.

##### Current status.

*Andrena* (incertae sedis) *lateralis* Morawitz, 1876 (synonymised by Gusenleitner and Schwarz 2001: 135).

##### Distribution.

Europe, Russia (to East Siberia), Caucasus, Turkey, Israel, Iran, Afghanistan, Central Asia.

#### ﻿5. Andrena aulica

Taxon classificationAnimaliaHymenopteraAndrenidae

Morawitz, 1876

FBBADC3D-7CF1-5500-B7F1-E508B1E2BC1B

Fig. 5

Andrena aulica Morawitz, 1876: 162, 166 (key), 187, ♀, ♂.

##### Type locality.

Panjakent (Tajikistan).

##### Published (original) locality.

Uzbekistan: Tashkent, Samarkand, Oalyk Gorge; Tajikistan: Panjakent, Fan [River], Varzaminor [= Ayni], Iori Gorge.

##### Lectotype (designated here).

♀, Пянджикентъ [Tajikistan, Panjakent, 39°30'N, 67°36'E] // к.[оллекция] Ф. Моравица [Collection of F. Morawitz] // *Andrenaaulica* Morawitz, ♀ [handwritten by F. Morawitz] // Paralectotypus *Andr.aulica* F. Mor., design. Osychnjuk, 1980 <red label> // Lectotypus *Andrenaaulica* Morawitz, 1876, design. Astafurova et al., 2022 // Zoological Institute St. Petersburg INS_HYM_0000299 [ZISP].

**Figure 5.**
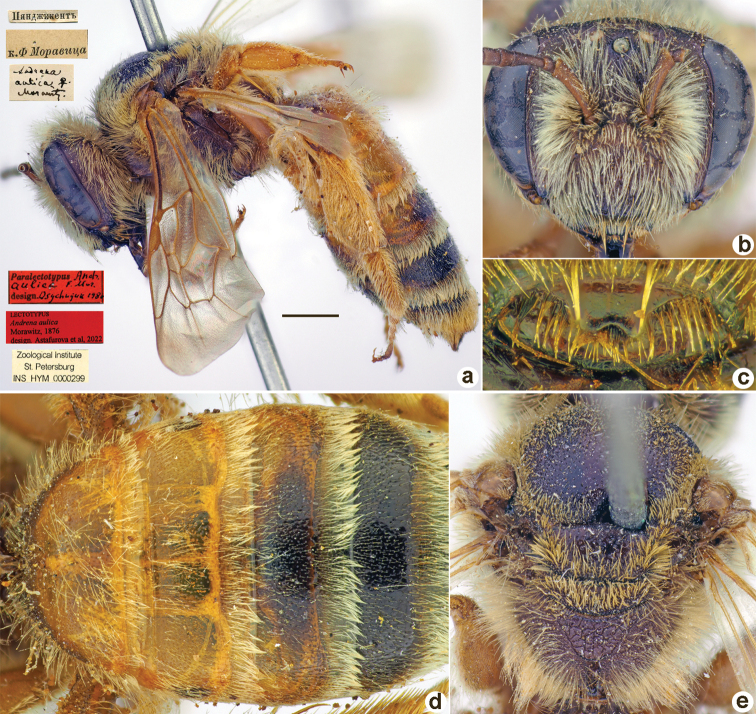
*Andrenaaulica* Morawitz, 1876, lectotype, female **A** habitus, lateral view and labels **B** head, frontal view **C** labrum, dorsal view **D** T1–T4, dorsal view **E** mesosoma, dorsal view. Scale bar: 1.0 mm.

##### Paralectotypes

**(21 ♀, 3 ♂).** 1 ♂, <golden circle> // Варзаминоръ // *aulica* Mor. Typ. ♂ [handwritten by F. Morawitz] // *Andr.ferghanica* F. Mor, ♂, Popov det, 1936; 1 ♀, Заравш. дол. // к.[оллекция] Ф. Моравица [Collection of F. Morawitz] // *aulica* Mor. [handwritten by F. Morawitz]; Paralectotypus *Andrenaaulica* Mor., design. Osychnjuk, 1980 <identical red labels on each paralectotype specimen> [ZISP]; 4 ♀, 8.[IV.1871] // Ташкентъ; 9 ♀, 20., 21., 23., 26., 30.[III], 9.[V.1869] // Самаркандъ; 3 ♀, 28., 29.[IV.1869] // Пянджикентъ; 1 ♀, 12.[VI.1870] // Фанъ; 2 ♀, 7.[VI.1870] // Варзаминоръ; 1 ♀, 2 ♂, 25.[III], 2., 7.[V.1869] // Заравш. дол. // Paralectotypus *Andrenaaulica* Morawitz, 1876, design. Astafurova et. al., 2022 <identical red labels on each paralectotype specimen> [ZMMU].

There is one female specimen labelled by Warcke as “Lectotype” [31. // Заравш. дол. // *Andrenaaulica* Mor. Typ. [handwritten by F. Morawitz] // Lectotypus Warncke 1975; ZMMU]. However, the label date (31.[II.1869]) does not correspond any date mentioned for type series by Morawitz (1876).

##### Current status.

Andrena (Plastandrena) aulica Morawitz, 1876.

##### Remarks.

According to Warncke (1967: 179) and Gusenleitner and Schwarz (2002: 130) *A.aulica* Morawitz, 1876 is a junior synonym of *A.bimaculata* (Kirby, 1802). However, Popov (1949), Osytshnjuk et al. (1978) and Astafurova et al. (2021) regarded *A.aulica* as a valid species. Taxonomic status of *A.bimaculata* sensu lato is problematic and requires a revision.

##### Distribution.

Russia (European part), Caucasus, Iran, Central Asia, India.

#### ﻿6. Andrena bairacumensis

Taxon classificationAnimaliaHymenopteraAndrenidae

Morawitz, 1876

C7F2DBE7-1D67-52CA-B700-AD8ACAE36A69

Fig. 6

Andrena bairacumensis Morawitz, 1876: 164 (key), 170, ♀.

##### Type locality.

Bairkum (Kazakhstan).

##### Published (original) locality.

Kazakhstan: Bayrakum [= Bairkum].

##### Holotype.

♀, 4.[V.1871] // Байракумъ [Kazakhstan, Syr-Darya River, Bairkum, 42°05'N, 68°10'E] // *Andrenabairacumensis* Mor. [handwritten by F. Morawitz] // Lectotypus Warncke 1975 <red label> // Holotypus *Andrenabairacumensis* Mor., 1876 <red label, labelled by Yu. Astafurova> [ZMMU].

**Figure 6.**
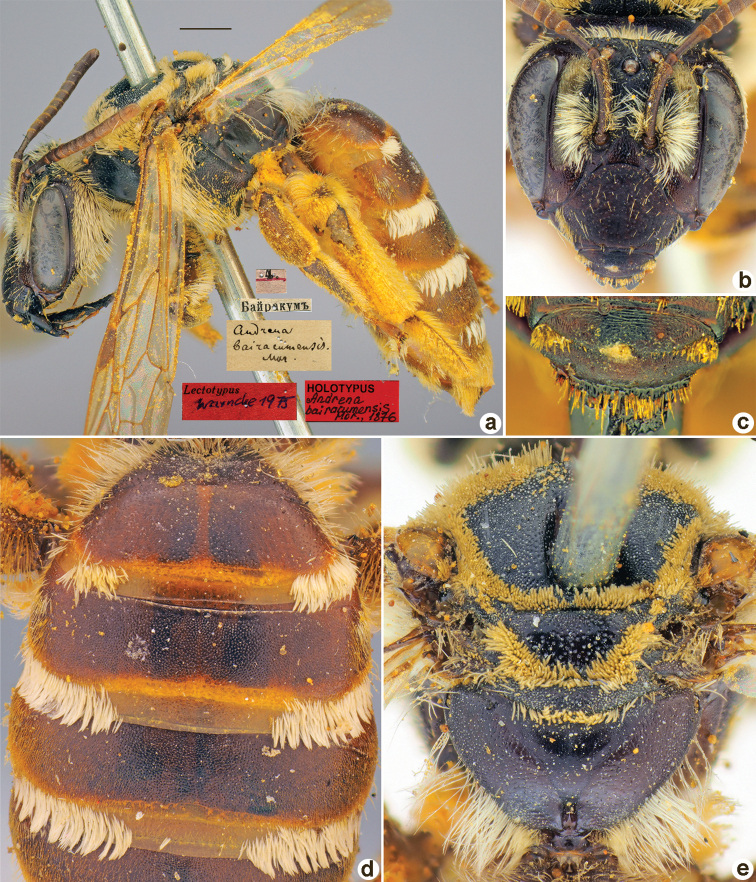
*Andrenabairacumensis* Morawitz, 1876, holotype, female **A** habitus, lateral view and labels **B** head, frontal view **C** labrum, dorsal view **D** T1–T3, dorsal view **E** mesosoma, dorsal view. Scale bar: 1.0 mm.

##### Current status.

Andrena (Leimelissa) bairacumensis Morawitz, 1876.

##### Remarks.

Description of male: Osytshnjuk 1984: 21, fig. 1.

##### Distribution.

Kazakhstan.

#### ﻿7. Andrena bicarinata

Taxon classificationAnimaliaHymenopteraAndrenidae

Morawitz, 1876

90CFDA06-597E-5519-A15B-62EBD3F0EC04

Fig. 7

Andrena bicarinata Morawitz, 1876: 164 (key), 197, ♀.

##### Type locality.

Tashkent (Uzbekistan).

##### Published (original) locality.

Uzbekistan: Tashkent, Katty-Kurgan, Urmitan [near Katty-Kurgan]; Tajikistan: Panjakent.

##### Lectotype (designated here).

♀, 11.[IV.1871] // Ташкентъ [Uzbekistan, Tashkent, 41°18'N, 69°16'E] // *Andrenabicarinata* Mor. [handwritten by F. Morawitz] // Paralectotypus *Andrenabicarinata* Mor., design. Osychnjuk, 1980 <red label> // *Andrenatuberculiventris* Mor. A. Osytshnjuk det. // Lectotypus *Andrenabicarinata* Morawitz, 1876, design. Astafurova et al., 2022 <red label> // Zoological Institute St. Petersburg INS_HYM_0000298 [ZISP].

**Figure 7.**
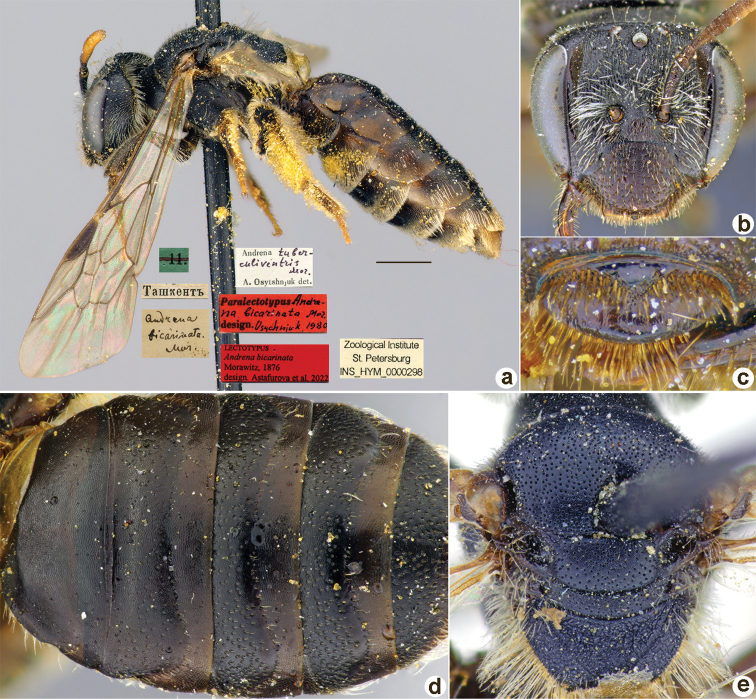
*Andrenabicarinata* Morawitz, 1876, lectotype, female **A** habitus, lateral view and labels **B** head, frontal view **C** labrum, dorsal view **D** T1–T5, dorsal view **E** mesosoma, dorsal view. Scale bar: 1.0 mm.

##### Paralectotypes

**(5 ♀, 1 ♂).** 1 ♀, 2.[IV.1871] // Ташкентъ // к.[оллекция] Ф. Моравица [Collection of F. Morawitz] // *Andrenabicarinata* Mor. [handwritten by F. Morawitz] // Paralectotypus *Andrenabicarinata* Mor., design. Osychnjuk, 1980 < red label > // *Andrenatuberculiventris*, D.A. Sidorov det.; 1 ♀, 5.[IV.1871] // к.[оллекция] Ф. Моравица [Collection of F. Morawitz] // *Andrenabicarinata F.Mor*, Cotype!, F. Morawitz det. // *Andrenatuberculiventris*, D.A. Sidorov det. [ZISP]; 1 ♀, 5.[V.1871] // Урмитанъ [Urmitan] // Lectotypus Warncke 1975 <red label> // A.tuberculiventris [det. A.Osytshnjuk]; 1 ♂, 5.[IV.1871] // Ташкентъ [Tashkent] // *Andrenatuberculiventris*, D.A. Sidorov det.; 1 ♀, 29.[VI.1869] // Katty-Kurgan // *Andrenatuberculiventris*, D.A. Sidorov det.; 1 ♀, 2.[V.1871] // Урмитанъ [Urmitan] // *Andrenatuberculiventris*, D.A. Sidorov det. // Paralectotypus *Andrenabicarinata* Morawitz, 1876, design. Astafurova et al., 2022 <identical red labels on each paralectotype specimen> [ZMMU].

##### Current status.

Andrena (Parandrenella) tuberculiventris Morawitz, 1876 (synonymised by Popov 1958: 118).

##### Distribution.

Uzbekistan, Tajikistan.

#### ﻿8. Andrena capillosa

Taxon classificationAnimaliaHymenopteraAndrenidae

Morawitz, 1876

76D969E1-5112-5B2B-92E5-C70764C11795

Fig. 8

Andrena capillosa Morawitz, 1876: 163 (key), 205, ♀.

##### Type locality.

Samarkand (Uzbekistan).

##### Published (original) locality.

Uzbekistan: Samarkand.

##### Lectotype.

♀, designated by Osytshnjuk et al. 2008: 49, ♀, 23.[II.1869] // Самаркандъ [Uzbekistan, Samarkand, 39°39'N, 66°57'E] // *Andrenacapillosa* Mor. [handwritten by F. Morawitz] // Lectotypus Warncke 1975 // Lectotypus *Andrenacapillosa* Morawitz, 1876, design. Osytshnjuk et al., 2008 <red label, labelled by Yu. Astafurova> [ZMMU].

**Figure 8.**
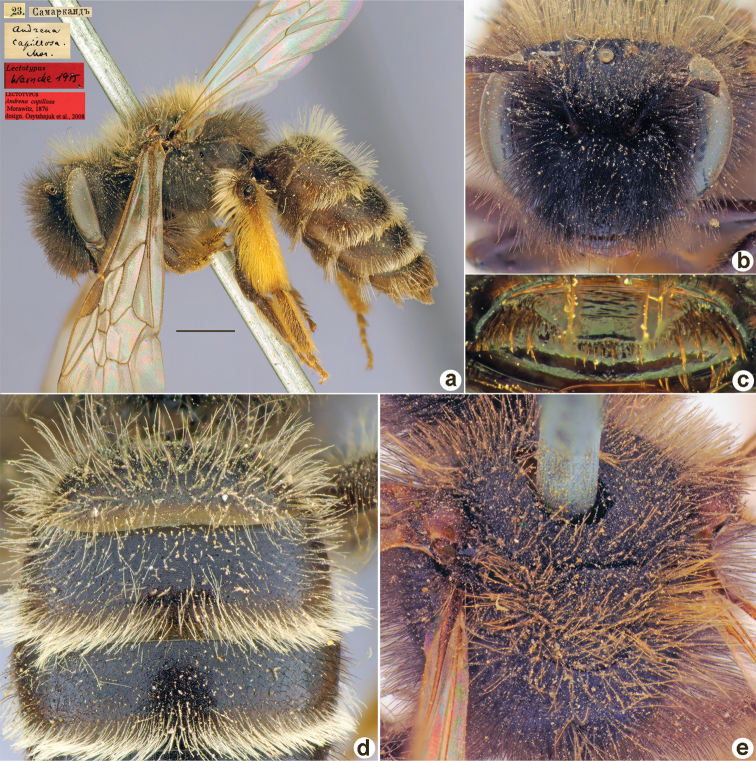
*Andrenacapillosa* Morawitz, 1876, lectotype, female **A** habitus, lateral view and labels **B** head, frontal view **C** labrum, dorsal view **D** T1–T3, dorsal view **E** mesosoma, dorsal view. Scale bar: 1.0 mm.

##### Paralectotypes

**(5 ♀).** 1 ♀, <golden circle>, 27.[II.1869] // Самаркандъ // *capillosa* Mor. Typ. [handwritten by F. Morawitz]; 4 ♀, 27.[II.1869], 3., 4.[III.1869] // Самаркандъ // к.[оллекция] Ф. Моравица [Collection of F. Morawitz] // Paralectotypus *Andrenacapillosa* Mor., design. Osychnjuk, 1980 <identical red labels on each paralectotype specimen> [ZISP].

##### Current status.

Andrena (Euandrena) capillosa Morawitz, 1876.

##### Remarks.

Description of male: Osytshnjuk et al. 2008: 50.

##### Distribution.

Uzbekistan, Tajikistan, Kazakhstan. The record from Russia (Western Siberia) by Krainov (2013) is doubtful.

#### ﻿9. Andrena carinifrons

Taxon classificationAnimaliaHymenopteraAndrenidae

Morawitz, 1876

71B337F1-1FD1-5B2B-A154-0B57EB5FE4C0

Fig. 9

Andrena carinifrons Morawitz, 1876: 164 (key), 198, ♀.

##### Type locality.

Turkistan Province (Kazakhstan).

##### Published (original) locality.

Kazakhstan: between the Karak Mts and Bairkum.

##### Lectotype.

♀, designated by Osytshnjuk et al. 2005: 161, Туркест.[анский] кр.[ай] [Kazakhstan, Turkistan Province, ≈ 42°46'N, 67°24'E] // к.[оллекция] Ф. Моравица [Collection of F. Morawitz] // *Andrenacarinifrons* Mor. [handwritten by F. Morawitz] // Lectotypus Warncke 1975 <red label> // Lectotypus *Andrenacarinifrons* Morawitz, 1876 design. by Osytchjuk et al., 2005 <red label, labelled by Yu. Astafurova> // Zoological Institute St. Petersburg INS_HYM_0000200.

**Figure 9.**
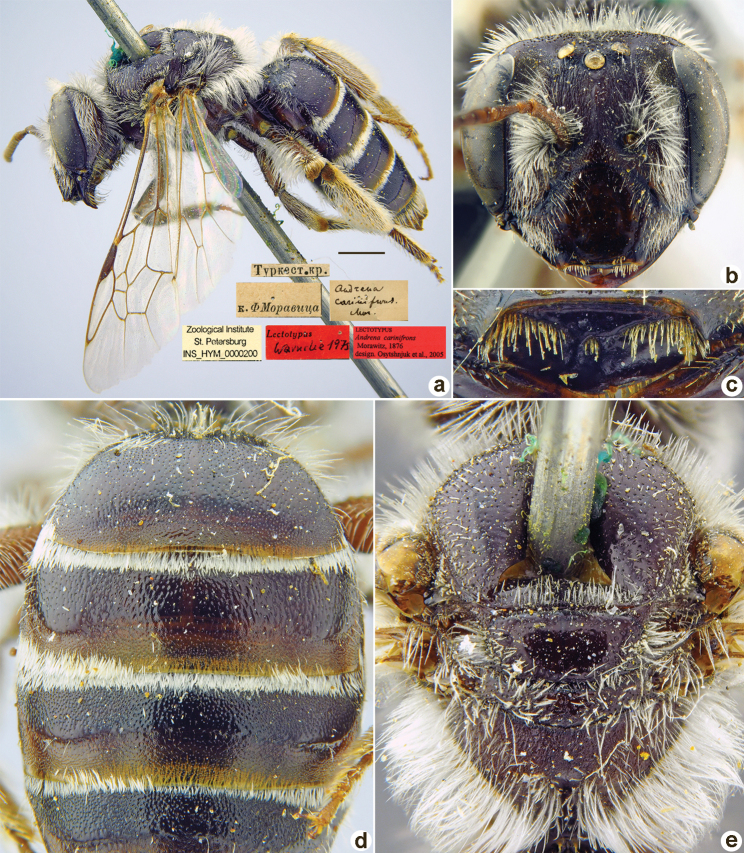
*Andrenacarinifrons* Morawitz, 1876, lectotype, female **A** habitus, lateral view and labels **B** head, frontal view **C** labrum, dorsal view **D** T1-T4, dorsal view **E** mesosoma, dorsal view. Scale bar: 1.0 mm.

##### Current status.

Andrena (Carinandrena) carinifrons Morawitz, 1876.

##### Remarks.

Description of male: Osytshnjuk 1993: 18, figs 2, 3.

##### Distribution.

Turkmenistan, Uzbekistan, Kazakhstan.

#### ﻿10. Andrena combusta

Taxon classificationAnimaliaHymenopteraAndrenidae

Morawitz, 1876

6B61927A-F633-5629-AF93-B179FBF73B45

Fig. 10

Andrena combusta Morawitz, 1876: 163, 165 (key), 189, ♀, ♂.

##### Type locality.

Kattakurgan (Uzbekistan).

##### Published (original) locality.

Uzbekistan: Tashkent and near Kattakurgan.

##### Lectotype (designated here).

♀, 29. [IV.1869] // Верхн.[ий] Заравш.[ан] [Uzbekistan, Upper Zaravshan, near Kattakurgan, 39°53'N, 66°15'E] // *Andrenacombusta* Mor. [handwritten by F. Morawitz] // Lectotypus Warncke, 1975 <red label> // Lectotypus *Andrenacombusta* Morawitz, 1876, design. Astafurova et al., 2022 <red label> [ZMMU].

**Figure 10.**
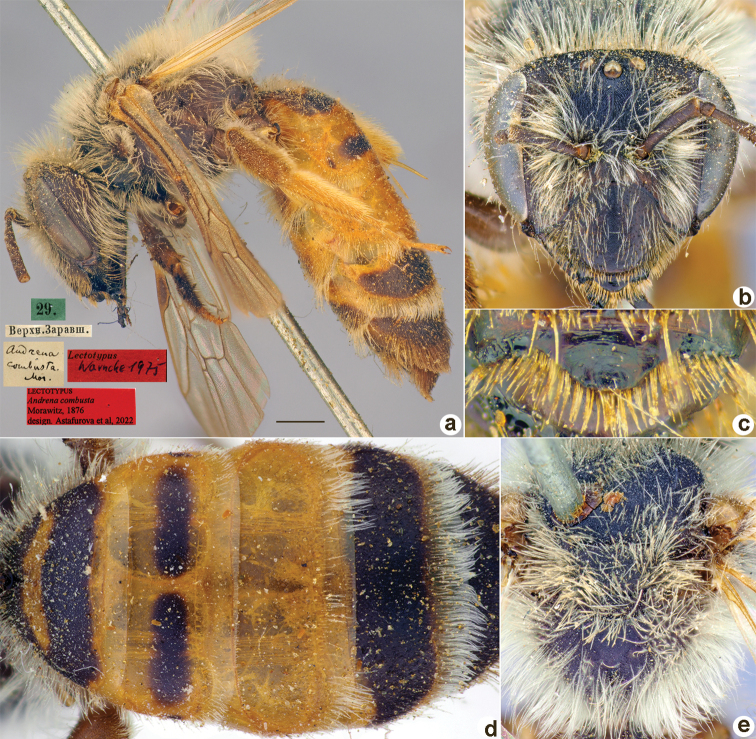
*Andrenacombusta* Morawitz, 1876, lectotype, female **A** habitus, lateral view and labels **B** head, frontal view **C** labrum, dorsal view **D** metasoma, dorsal view **E** mesosoma, dorsal view. Scale bar: 1.0 mm.

##### Paralectotypes

**(1 ♀, 1 ♂).** 1 ♀, 29. [IV.1869] // Верхн. Заравш. [Upper Zaravshan]; 1 ♂, 11.[IV.1871] // Ташкентъ // Paralectotypus *Andrenacombusta* Morawitz, 1876, design. Astafurova et al., 2022 <identical red labels on each paralectotype specimen> [ZMMU].

##### Current status.

Andrena (Truncandrena) combusta Morawitz, 1876.

##### Distribution.

Azerbaijan, Turkey, Syria, Iran, Afghanistan, Uzbekistan, Tajikistan.

#### ﻿11. Andrena comparata

Taxon classificationAnimaliaHymenopteraAndrenidae

Morawitz, 1876

172B32CF-0E9E-56B0-BEA5-69B9EF64D435

Fig. 11

Andrena comparata Morawitz, 1876: 166 (key), 188, ♂.

##### Type locality.

Tashkent (Uzbekistan).

##### Published (original) locality.

Uzbekistan: Tashkent and Samarkand.

##### Lectotype (designated here).

♂, 9.[III.1871] // Ташкентъ [Uzbekistan, Tashkent, 41°18'N, 69°16'E] // *Andrenacomparata* Mor. [handwritten by F. Morawitz] // к.[оллекция] Ф. Моравица [Collection of F. Morawitz] // *Andr.aulica* F. Mor, ♂, Popov 1936 det. // Paralectotypus *Andr.comparata* Mor., design. Osychnjuk, 1980 <red label> // Lectotypus *Andrenacomparata* Morawitz, 1876, design. Astafurova et al., 2022 <red label> // Zoological Institute St. Petersburg INS_HYM_0000297 [ZISP].

**Figure 11.**
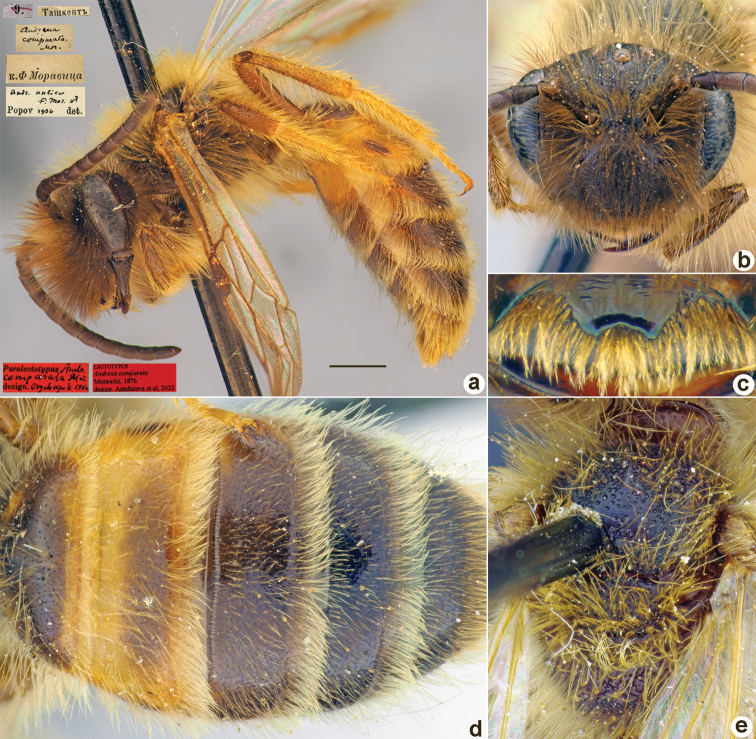
*Andrenacomparata* Morawitz, 1876, lectotype, male **A** habitus, lateral view and labels **B** head, frontal view **C** labrum, dorsal view **D** metasoma, dorsal view **E** mesosoma, dorsal view. Scale bar: 1.0 mm.

##### Paralectotypes

**(9 ♂).** 1 ♂, <golden circle>, 21.[III.1869] // Самаркандъ [Samarkand] // *Andrenacomparata* Mor. Typ. [handwritten by F. Morawitz] // *Andr.aulica* F.Mor, ♂, Popov det. 1936 // Paralectotypus *Andrenacomparata* Mor., design. Osychnjuk, 1980 <red label> [ZISP]; 1 ♂, 21.[III.1869] // Самаркандъ // *Andrenacomparata* Mor. [handwritten by F. Morawitz] // Lectotypus Warncke 1975 <red label> // *Andrenaaulica*, D.A. Sidorov det., 2022; 7 ♂, 21., 26., 30.[III.1869] // Самаркандъ // Paralectotypus *Andrenacomparata* Morawitz, 1876, design. Astafurova et al., 2022 <identical red labels on each paralectotype specimen> [ZMMU].

##### Current status.

Andrena (Plastandrena) aulica Morawitz, 1876 (synonymised by Popov 1949: 392).

##### Remarks.

Listed as Andrena (Plastandrena) bimaculata (Kirby, 1802) by Gusenleitner and Schwarz 2002: 130 (see remarks on *Andrenaaulica*, above).

##### Distribution.

Russia (European part), Caucasus, Iran, Central Asia, India.

#### ﻿12. Andrena corallina

Taxon classificationAnimaliaHymenopteraAndrenidae

Morawitz, 1876

50D305FD-02E6-5309-B6E0-03759C1DE0F6

Fig. 12

Andrena corallina Morawitz, 1876: 162 (key), 203, ♀.

##### Type locality.

Urmetan (Tajikistan).

##### Published (original) locality.

Tajikistan: Urmitan.

##### Lectotype (designated here).

♀, 2.[V.1869] // Урмитанъ [Tajikistan, Urmetan, 39°26'N, 68°15'E] // *Andrenacorallina* Mor. [handwritten by F. Morawitz] // Lectotypus Warncke 1975 // Lectotypus *Andrenacorallina* Morawitz, 1876, design. Astafurova et al., 2022 <red label> [ZMMU].

**Figure 12.**
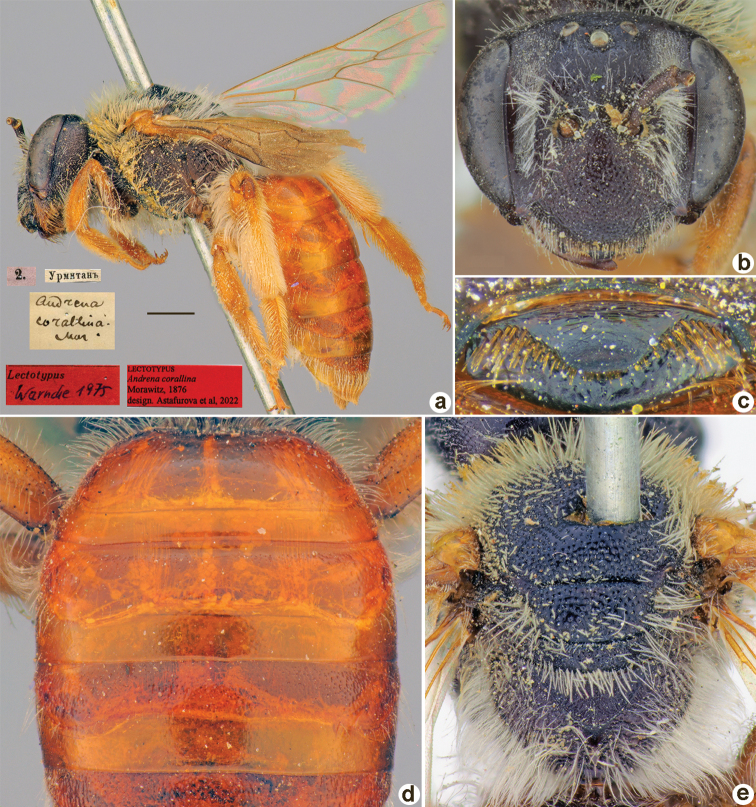
*Andrenacorallina* Morawitz, 1876, lectotype, female **A** habitus, lateral view and labels **B** head, frontal view **C** labrum, dorsal view **D** T1–T3, dorsal view **E** mesosoma, dorsal view. Scale bar: 1.0 mm.

##### Current status.

Andrena (Trachandrena) discophora Morawitz, 1876 (synonymised by Popov 1958: 159).

##### Distribution.

Uzbekistan, Tajikistan, Kazakhstan.

#### ﻿13. Andrena discophora

Taxon classificationAnimaliaHymenopteraAndrenidae

Morawitz, 1876

2A1A6A11-E2A5-5BE0-ACE5-8E5EECBF9249

Fig. 13

Andrena discophora Morawitz, 1876: 162, 166 (key), 202, ♀, ♂.

##### Type locality.

Tashkent (Uzbekistan).

##### Published (original) locality.

Uzbekistan: Tashkent.

##### Lectotype (designated here).

♀, 8.[IV.1871] // Ташкентъ [Uzbekistan, Tashkent, 41°18'N, 69°16'E] // *Andrenadiscophora* Mor. [handwritten by F. Morawitz] // Lectotypus Warncke 1975 <red label> // Lectotypus *Andrenadiscophora* Morawitz, 1876, design. Astafurova et al., 2022 <red label> [ZMMU].

**Figure 13.**
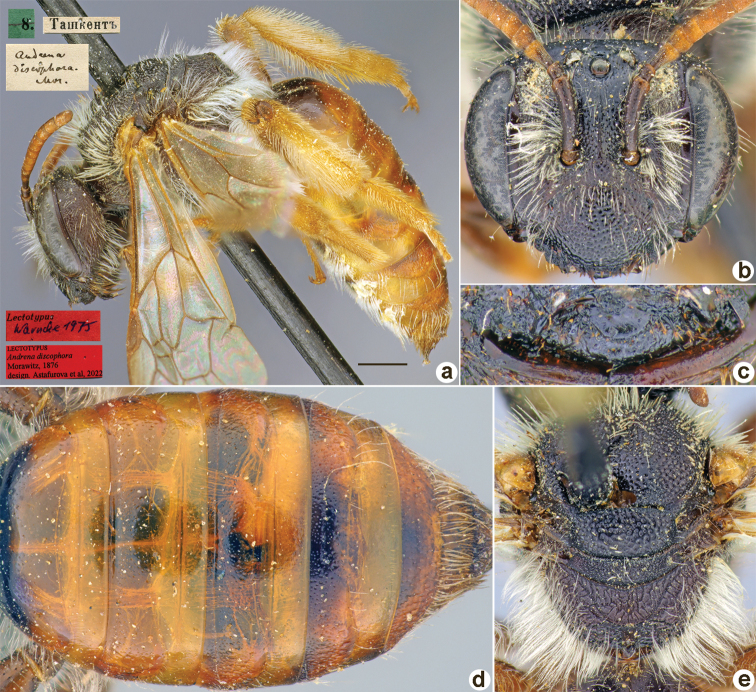
*Andrenadiscophora* Morawitz, 1876, lectotype, female **A** habitus, lateral view and labels **B** head, frontal view **C** labrum, dorsal view **D** metasoma, dorsal view **E** mesosoma, dorsal view. Scale bar: 1.0 mm.

##### Paralectotypes

**(21 ♀, 9 ♂).** 1 ♂, <golden circle> // 28.[II.1871] // Ташкентъ // *discophora* Mor. ♂, Typ. [handwritten by F. Morawitz]; 1 ♀, 4 ♂, Ташкентъ // к.[оллекция] Ф. Моравица [Collection of F. Morawitz] // *Andrenadiscophora* Mor. [handwritten by F. Morawitz]; 1 ♀, <golden circle> // 11.[IV.1871] // Ташкентъ // *discophora* Mor. ♀, Typ. [handwritten by F. Morawitz]; 2 ♀, 8.[IV.1871] and 27.[III.1987] // Ташкентъ // к.[оллекция] Ф. Моравица [Collection of F. Morawitz]; 1 ♀, 11.[IV.1981] // Ташкентъ // к.[оллекция] Ф. Моравица [Collection of F. Morawitz] // *Andr.discophora* F. Mor., Popov det. // Paralectotypus *Andrena* Mor., design. Osychnjuk, 1980 <identical red labels on each paralectotype specimen> [ZISP]; 16 ♀, 4 ♂, [28.II–11.IV.1871] // Ташкентъ // Paralectotypus *Andrenadiscophora* Morawitz, 1876, design. Astafurova et al., 2022 <identical red labels on each paralectotype specimen> [ZMMU].

##### Current status.

Andrena (Trachandrena) discophora Morawitz, 1876.

##### Distribution.

Uzbekistan, Tajikistan, Kazakhstan.

#### ﻿14. Andrena fedtschenkoi

Taxon classificationAnimaliaHymenopteraAndrenidae

Morawitz, 1876

507C9637-6F8D-5654-8D5F-0E328CC6C9BC

Fig. 14

Andrena Fedtschenkoi Morawitz, 1876: 162, 165 (key), 184, ♀, ♂.

##### Type locality.

Chardara (Kazakhstan).

##### Published (original) locality.

Kazakhstan: Kysyl-Kum [desert] near draw-well Chakany, Karak steppe, Chardara; Uzbekistan: Zeravshan River valley.

##### Lectotype (designated here).

♀, Чардара [Kazakhstan, Chardara, 41°18'N, 67°57'E] // *AndrenaFedtschenkoi* Mor. [handwritten by F. Morawitz] // к.[оллекция] Ф. Моравица [Collection of F. Morawitz] // Paralectotypus *Andrenafedtschenkoi* Mor., design. Osychnjuk, 1980 <red label> // Lectotypus *Andrenafedtschenkoi* Morawitz, 1876, design. Astafurova et al., 2022 <red label> // Zoological Institute St. Petersburg INS_HYM_0000296.

**Figure 14.**
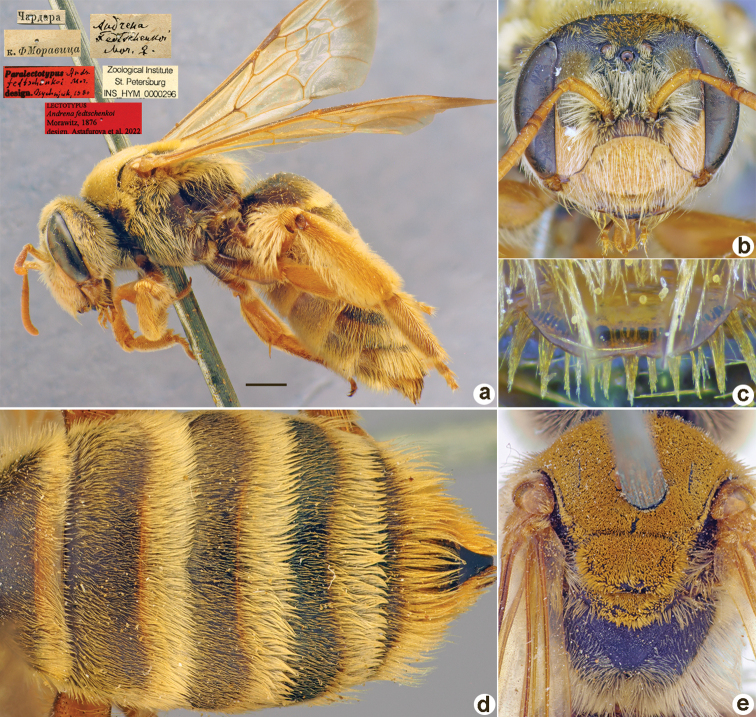
*Andrenafedtschenkoi* Morawitz, 1876, lectotype, female **A** habitus, lateral view and labels **B** head, frontal view **C** labrum, dorsal view **D** metasoma, dorsal view **E** mesosoma, dorsal view. Scale bar: 1.0 mm.

##### Paralectotypes

**(7 ♀, 11 ♂).** 1 ♀, Кизилъкум [Kyzylkum] // к.[оллекция] Ф. Моравица [Collection of F. Morawitz] // *fedtschenkoi* F. Morawitz, ♀ [handwritten by F. Morawitz]; 3 ♂, Чардара // к.[оллекция] Ф. Моравица [Collection of F. Morawitz] // *AndrenaFedtschenkoi* Mor. [handwritten by F. Morawitz]; 1 ♂, 25.[IV.1869] // Заравшан.[ская] дол.[ина] [Zeravshan River valley] // *fedtschenkoi* F. Morawitz, ♂ Typ. [handwritten by F. Morawitz] // Paralectotypus *Andrenafedtschenkoi* Mor., design. Osychnjuk, 1980 <red label> [ZISP]; 1 ♀, 28.[IV.1871] // Кизилъкум // *AndrenaFedtschenkoi* Mor. [handwritten by F. Morawitz] // Lectotypus Warncke 1975 <red label>; 4 ♀, 4 ♂, 25.[IV.1869], 12., 13., 18., 23.[V.1869] // Заравш. дол.; 3 ♂, 25.[IV.1871] // Чардара; 1 ♀, 5.[V.1871] // Карак.[ская] степь [Karak steppe] // Paralectotypus *Andrenafedtschenkoi* Morawitz, 1876, design. Astafurova et al., 2022 <identical red labels on each paralectotype specimen> [ZMMU].

##### Current status.

Andrena (Ulandrena) fedtschenkoi Morawitz, 1876.

##### Distribution.

Turkmenistan, Uzbekistan, Tajikistan, Kazakhstan.

#### ﻿15. Andrena ferghanica

Taxon classificationAnimaliaHymenopteraAndrenidae

Morawitz, 1876

09C1F838-36F7-50E1-B769-D69BF110A5D9

Fig. 15

Andrena ferghanica Morawitz, 1876: 163 (key), 189, ♀.

##### Type locality.

Alai Mts, Kavuk Pass (Kyrgyzstan).

##### Published (original) locality.

Kyrgyzstan: Kavuk Pass.

##### Holotype.

♀, 24.[VI.1871] // Алай [Kyrgyzstan, Alay Mts, Kavuk Pass, 39°40'N, 72°15'E] // к.[оллекция] Ф. Моравица [Collection of F. Morawitz] // *Andrenaferghanica* Morawitz [handwritten by F. Morawitz] // Lectotypus *Andrenaferghanica* Mor., design. Osychnjuk, 1980 <red label> // Holotypus *Andrenaferghanica* Mor., 1876 <red label, labelled by Yu. Astafurova> // Zoological Institute St. Petersburg INS_HYM_0000261 [ZISP].

**Figure 15.**
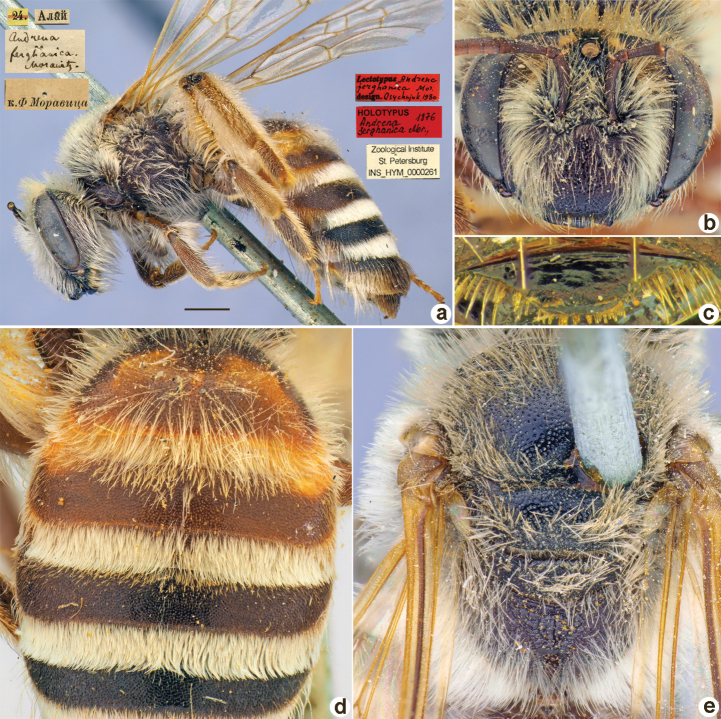
*Andrenaferghanica* Morawitz, 1876, holotype, female **A** habitus, lateral view and labels **B** head, frontal view **C** labrum, dorsal view **D** T1–T4, dorsal view **E** mesosoma, dorsal view. Scale bar: 1.0 mm.

##### Current status.

Andrena (Plastandrena) ferghanica Morawitz, 1876.

##### Remarks.

Description of male: Morawitz 1876: 187 as *A.aulica*, in partim (according to Popov 1949: 391).

##### Distribution.

Iran, Pakistan, Central Asia.

#### ﻿16. Andrena flavitarsis

Taxon classificationAnimaliaHymenopteraAndrenidae

Morawitz, 1876

BC8174E3-1DF8-5368-A470-59979B36B891

Fig. 16

Andrena flavitarsis Morawitz, 1876: 163, 166 (key), 204, ♀, ♂.

##### Type locality.

Tashkent (Uzbekistan).

##### Published (original) locality.

Uzbekistan: Tashkent.

##### Lectotype.

♀, designated by Osytshnjuk et al. 2008: 47, 3.[IV.1871] // Ташкентъ [Uzbekistan, Tashkent, 41°18'N, 69°16'E] // *Andrenaflavitarsis* Mor. ♀ [handwritten by F. Morawitz] // Lectotypus Warncke 1975 <red label> // Lectotypus *Andrenaflavitarsis* Morawitz, 1876, design. Osytshnjuk et al., 2008 <red label, labelled by Yu. Astafurova> [ZMMU].

**Figure 16.**
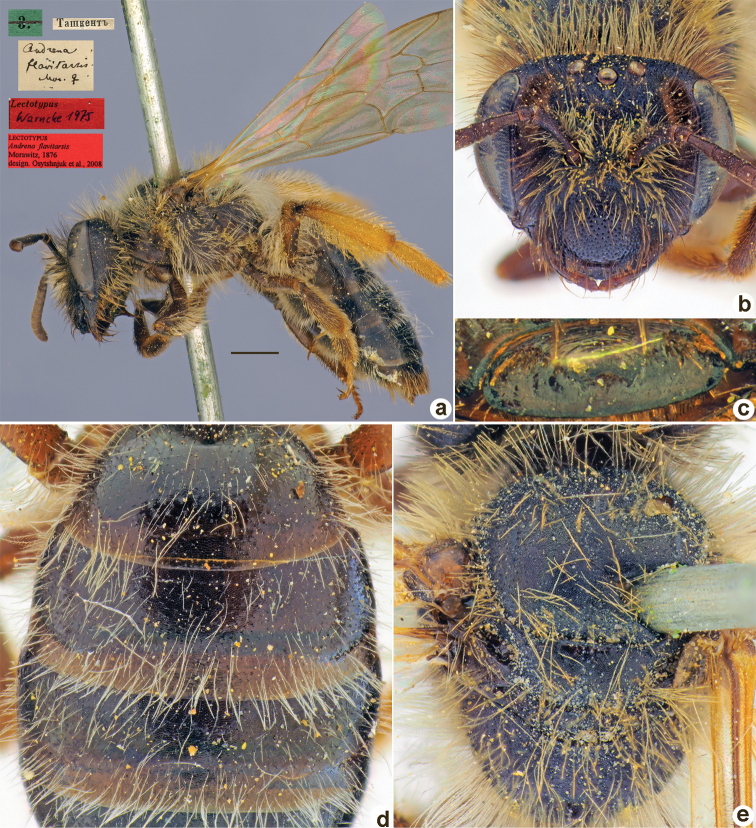
*Andrenaflavitarsis* Morawitz, 1876, lectotype, female **A** habitus, lateral view and labels **B** head, frontal view **C** labrum, dorsal view **D** T1–T3, dorsal view **E** mesosoma, dorsal view. Scale bar: 1.0 mm.

##### Paralectotypes

**(1 ♀, 1 ♂).** 1 ♀, 5.[IV.1871] // Ташкентъ [Tashkent] // к.[оллекция] Ф. Моравица [Collection of F. Morawitz] // *Andrenaflavitarsis* Mor. [handwritten by F. Morawitz] // *Andrenaflavitarsis* Mor., design. Osychnjuk, 1980 < red label > [ZISP]; 1 ♂, 16.[III.1871] // Ташкентъ [Tashkent] // *Andrenaflavitarsis* Mor. [handwritten by F. Morawitz] // Paralectotypus *Andrenaflavitarsis* Morawitz, 1876, design. Osytshnjuk et al., 2008 <red label, labelled by Yu. Astafurova> [ZMMU].

##### Current status.

Andrena (Euandrena) flavitarsis Morawitz, 1876.

##### Distribution.

Uzbekistan.

#### ﻿17. Andrena fuscicollis

Taxon classificationAnimaliaHymenopteraAndrenidae

Morawitz, 1876

7919437C-157C-5B68-91C2-DB45AB668844

Fig. 17

Andrena fuscicollis Morawitz, 1876: 164, 165 (key), 208, ♀, ♂.

##### Type locality.

Tashkent (Uzbekistan).

##### Published (original) locality.

Uzbekistan: Tashkent.

##### Lectotype.

♂, designated by Osytshnjuk et al. 2008: 73, 11.[IV.1871] // Ташкентъ [Uzbekistan, Tashkent, 41°18'N, 69°16'E] // *Andrenafuscicollis* Mor. [handwritten by F. Morawitz] // Lectotypus Warncke 1975 <red label> // Lectotypus *Andrenafuscicollis* Morawitz, 1876, design. Osytshnjuk et al., 2008 <red label, labelled by Yu. Astafurova> [ZMMU].

**Figure 17.**
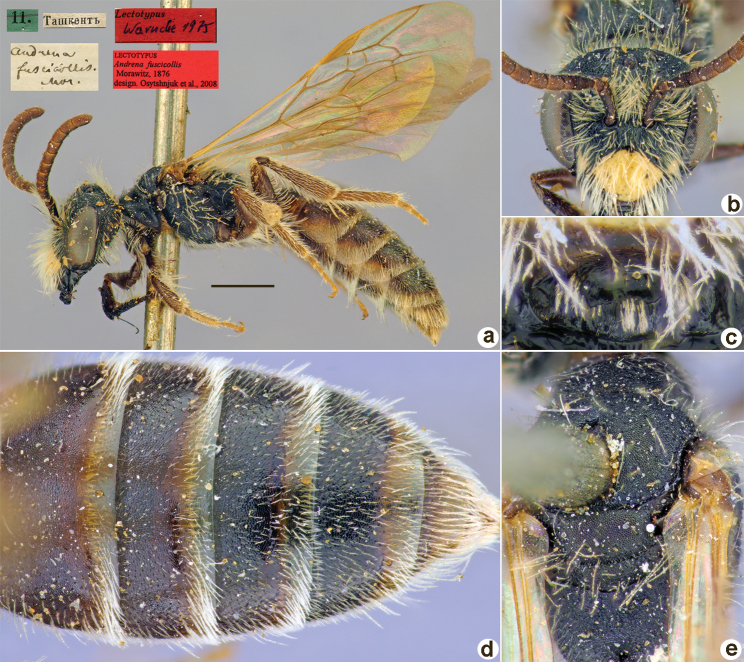
*Andrenafuscicollis* Morawitz, 1876, lectotype, male **A** habitus, lateral view and labels **B** head, frontal view **C** labrum, dorsal view **D** metasoma, dorsal view **E** mesosoma, dorsal view. Scale bar: 1.0 mm.

##### Paralectotypes

**(5 ♀, 5 ♂).** 1 ♂, <golden circle>, 1.[IV.1871] // Ташкентъ // *fuscicollis* Mor. ♂ [handwritten by F. Morawitz]; 4 ♂, 5., 10.[IV.1871] // Ташкентъ // к.[оллекция] Ф. Моравица [Collection of F. Morawitz]; 1 ♀, <golden circle>, 10.[IV.1871] // Ташкентъ // *fuscicollis* Mor. Typ., ♀ [handwritten by F. Morawitz] // Paralectotypus *Andrenafuscicollis* Mor., design. Osychnjuk, 1980 <identical red labels on each paralectotype specimen> [ZISP]; 4 ♀, 1.,10., 11.[IV.1871] Ташкентъ // Paralectotypus *Andrenafuscicollis* Morawitz, 1876, design. Osytshnjuk et al., 2008 <identical red labels on each paralectotype specimen, labelled by Yu. Astafurova> [ZMMU].

##### Current status.

Andrena (Fuscandrena) fuscicollis Morawitz, 1876.

##### Remarks.

The collection date in the original publication is “from 11 February to 11 March”. However, the lectotype and paralectotype specimens designated by A. Osytshnjuk are labelled as collected in April [green label]. Probably “March” was mistakenly mentioned in the Morawitz’ publication instead of April.

##### Distribution.

Turkmenistan, Uzbekistan, Tajikistan.

#### ﻿18. Andrena hieroglyphica

Taxon classificationAnimaliaHymenopteraAndrenidae

Morawitz, 1876

C32A70DA-D344-5CB0-8FDB-BF59457419E3

Fig. 18

Andrena hieroglyphica Morawitz, 1876: 163 (key), 192, ♀.

##### Type locality.

Sokh Enclave (Uzbekistan).

##### Published (original) locality.

Uzbekistan: near Sokh.

##### Holotype.

♀, 28.[VI.1871] // Сохъ [Uzbekistan, Sokh Enclave, 39°57'N, 71°07'E] // *Andrenahieroglyphica* Mor. [handwritten by F. Morawitz] // Lectotypus Warncke 1975 <red label> // Holotypus *Andrenahieroglyphica* Mor., 1876 <red label, labelled by Yu. Astafurova> [ZMMU].

**Figure 18.**
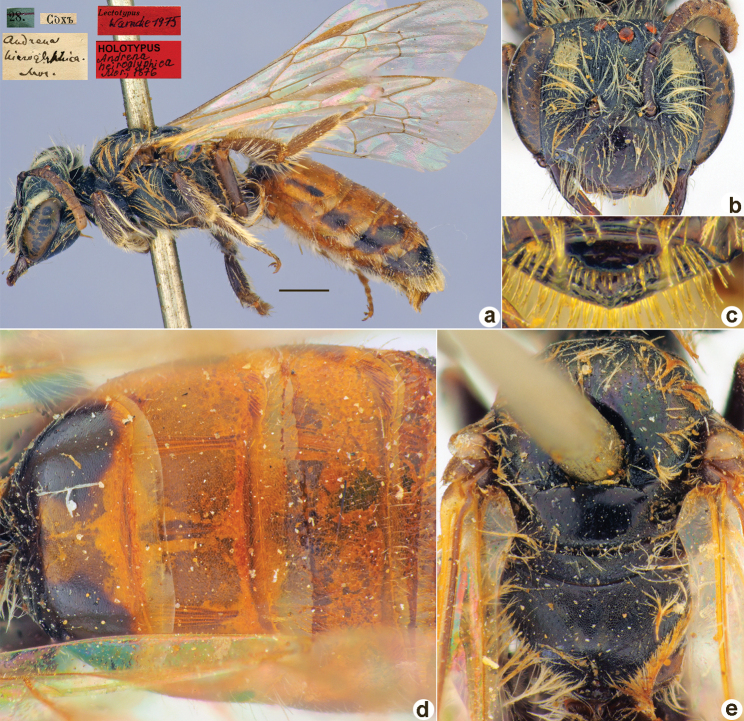
*Andrenahieroglyphica* Morawitz, 1876, holotype, female **A** habitus, lateral view and labels **B** head, frontal view **C** labrum, dorsal view **D** T1–T3, dorsal view **E** mesosoma, dorsal view. Scale bar: 1.0 mm.

##### Current status.

*Andrena* (incertae sedis) *hieroglyphica* Morawitz, 1876.

##### Remarks.

Description of male: Morawitz 1876: 204, as *Andrenatemporalis* Morawitz, 1876 (synonymised by Osytshnjuk 1984: 3).

The lectotype designation by Osytshnjuk et al. (2005: 151) is unnecessary as the species was described from a single male that was directly written about by Morawitz (1876: 192).

##### Distribution.

Iran, Turkmenistan, Uzbekistan, Tajikistan, Pakistan.

#### ﻿19. Andrena infirma

Taxon classificationAnimaliaHymenopteraAndrenidae

Morawitz, 1876

19934B0E-CBF8-514A-9109-668CCF477E38

Fig. 19

Andrena infirma Morawitz, 1876: 164, 166 (key), 195, ♀, ♂.

##### Type locality.

Ayni (Tajikistan).

##### Published (original) locality.

Tajikistan: Varzaminor, near Iskander-Kul Lake; Kyrgyzstan/Tajikistan: Khodzha-Chiburgan River.

##### Lectotype.

♂, designated by by Osytshnjuk et al. 2008: 199, 8.[VI.1869] // Варзаминоръ [Tajikistan, Varzaminor (= Ayni), 39°23'N, 68°32'E] // *Andrenainfirma* Mor. [handwritten by F. Morawitz] // Lectotypus Warncke 1975 <red label> // Lectotypus *Andrenainfirma* Morawitz, 1876, design. Osytshnjuk et al., 2008 <red label, labelled by Yu. Astafurova> [ZMMU].

**Figure 19.**
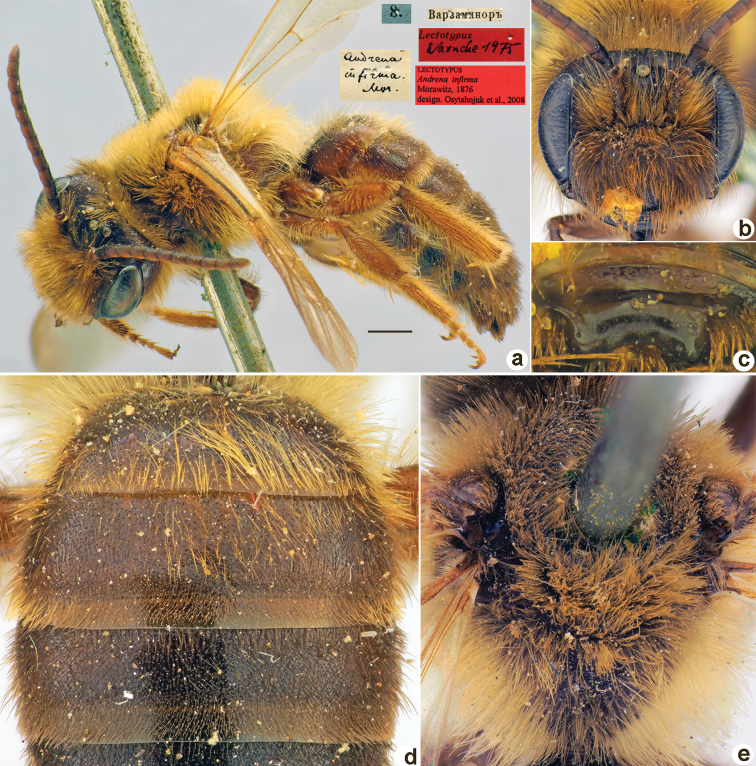
*Andrenainfirma* Morawitz, 1876, lectotype, male **A** habitus, lateral view and labels **B** head, frontal view **C** labrum, dorsal view **D** T1–T3, dorsal view **E** mesosoma, dorsal view. Scale bar: 1.0 mm.

##### Paralectotypes

**(1 ♀, 4 ♂).** 1 ♂, 8.[VI.1870] // Варзаминоръ // к.[оллекция] Ф. Моравица [Collection of F. Morawitz]; 1 ♂, <golden circle> // 21.[VI.1870] // Искандеръ [Iskander] // *infirma* Mor. Typ. [handwritten by F. Morawitz]; 1 ♂, 21.[VI.1870] // Искандеръ // *Andrenainfirma* Mor. [handwritten by F. Morawitz] // к.[оллекция] Ф. Моравица [Collection of F. Morawitz] // Paralectotypus *Andrenainfirma* Mor., design. Osytshnjuk, 1980 <identical red labels on each paralectotype specimen> [ZISP]; 1 ♀ [without head], 9.[VI.1869] // Варзаминоръ // Федченко [Fedtschenko leg.]; 1 ♂, 26.[VI.1871] // Чибурганъ [Khodzha-Chiburgan River] // Paralectotypus *Andrenainfirma* Morawitz, 1876., design. Osytshnjuk et al., 2008 <red label, labelled by Yu. Astafurova> [ZMMU].

##### Current status.

Andrena (Melandrena) infirma Morawitz, 1876.

##### Remarks.

Osytshnjuk et al. (2008: 199) mistakenly mentioned sex of the lectotype as a female instead of male.

##### Distribution.

Afghanistan, Central Asia.

#### ﻿20. Andrena initialis

Taxon classificationAnimaliaHymenopteraAndrenidae

Morawitz, 1876

FC99A7E8-DD11-5754-AFDC-B0A7621D887E

Fig. 20

Andrena initialis Morawitz, 1876: 164, 166 (key), 199, ♀, ♂.

##### Type locality.

Shardara District (Kazakhstan).

##### Published (original) locality.

Kazakhstan: “between Keles [River] and Kosaral [Lake]”.

##### Lectotype (designated here).

♀, 24.[IV.1871] // Косаралъ [Kazakhstan, “Kosaral Lake” (now Shardara Reservoir), ≈ 41°10'N, 68°06'E] // *Andrenainitialis* Mor. [handwritten by F. Morawitz] // к.[оллекция] Ф. Моравица [Collection of F. Morawitz] // Paralectotypus *Andrenainitialis* Mor., design. Osychnjuk, 1980 // Lectotypus *Andrenainitialis* Morawitz, 1876, design. Astafurova et al., 2022 <red label> // Zoological Institute St. Petersburg INS_HYM_0000295.

**Figure 20.**
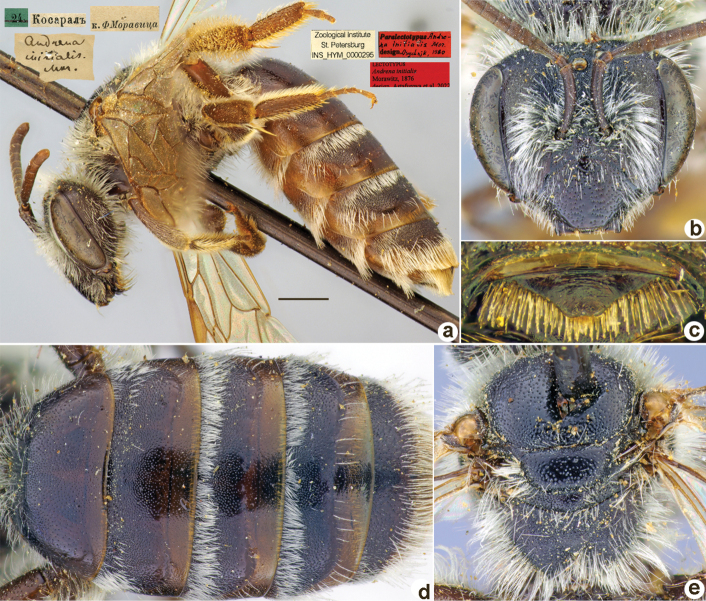
*Andrenainitialis* Morawitz, 1876, lectotype, female **A** habitus, lateral view and labels **B** head, frontal view **C** labrum, dorsal view **D** metasoma, dorsal view **E** mesosoma, dorsal view. Scale bar: 1.0 mm.

##### Paralectotypes

**(3 ♀, 1 ♂).** 1 ♀, Косаралъ // к.[оллекция] Ф. Моравица [Collection of F. Morawitz] // Paralectotypus *Andrenainitialis* Mor., design. Osychnjuk, 1980 [ZISP]; 1 ♀, 24.[IV.1871] // Косаралъ // *Andrenainitialis* Mor. [handwritten by F. Morawitz] // Lectotypus Warncke 1975; 1 ♀, 24.[IV.1871] // Косаралъ; 1 ♂, 24.[IV.1871] // Косаралъ // *Andrenalateralis*, D.A. Sidorov det., 2022 // Paralectotypus *Andrenainitialis* Morawitz, 1876, design. Astafurova et al., 2022 <identical red labels on each paralectotype specimen> [ZMMU].

##### Current status.

*Andrena* (incertae sedis) *initialis* Morawitz, 1876.

##### Remarks.

Male unknown. The single male from the type series belongs to *Andrena* (incertae sedis) *lateralis* Morawitz, 1876, as was mentioned by Gusenleitner and Schwarz (2001: 133).

##### Distribution.

Uzbekistan, Kazakhstan.

#### ﻿21. Andrena laeviventris

Taxon classificationAnimaliaHymenopteraAndrenidae

Morawitz, 1876

D63503E2-A06A-541E-8961-220548D83CFA

Fig. 21

Andrena laeviventris Morawitz, 1876: 163 (key), 182, ♀.

##### Type locality.

Obburdon (Tajikistan).

##### Published (original) locality.

Uzbekistan: Gus [near Urgut]; Tajikistan: Pyandzhikent, Obburden [Obburdon].

##### Lectotype (designated here).

♀, 4.[VI.1870] // Оббурденъ [Tajikistan, Obburdon, 40°25'N, 69°18'E] // *Andrenalaeviventris* Mor. [handwritten by F. Morawitz] // Lectotypus Warncke 1975 <red label> // Lectotypus *Andrenalaeviventris* Morawitz, 1876, design. Astafurova et al., 2022 <red label> [ZMMU].

**Figure 21.**
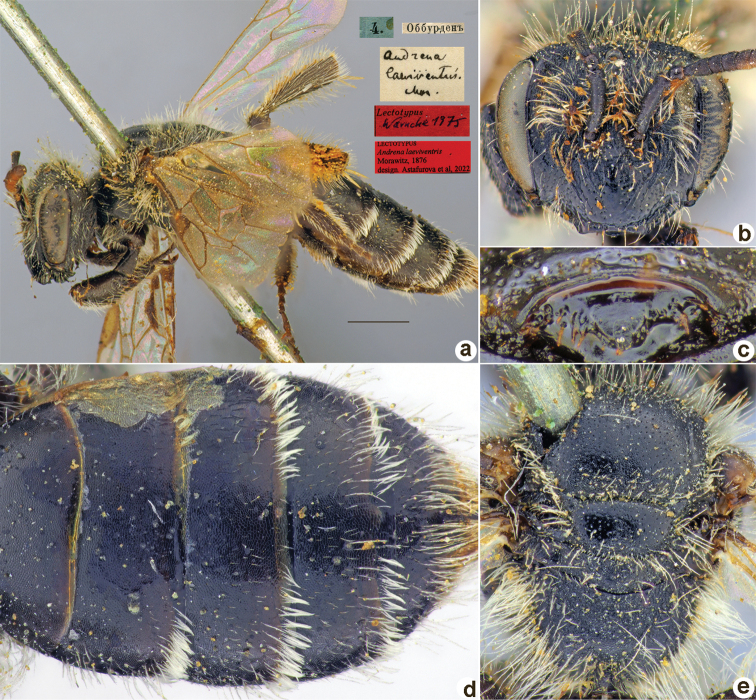
*Andrenalaeviventris* Morawitz, 1876, lectotype, female **A** habitus, lateral view and labels **B** head, frontal view **C** labrum, dorsal view **D** metasoma, dorsal view **E** mesosoma, dorsal view. Scale bar: 1.0 mm.

##### Paralectotype.

1 ♀, 24.[V.1869] // Заравш.[анская] дол.[ина] [Zaravshan River Valley] // Paralectotypus *Andrenalaeviventris* Morawitz, 1876, design. Astafurova et al., 2022 <red label> [ZMMU].

##### Current status.

Andrena (Aciandrena) laeviventris Morawitz, 1876.

##### Remarks.

Male unknown (according to Gusenleitner and Schwarz 2002: 410).

##### Distribution.

Uzbekistan, Tajikistan.

#### ﻿22. Andrena lateralis

Taxon classificationAnimaliaHymenopteraAndrenidae

Morawitz, 1876

5E95C847-D890-53BE-AF42-B943B500DC75

Fig. 22

Andrena lateralis Morawitz, 1876: 163, 166 (key), 200, ♀, ♂.

##### Type locality.

Shardara District (Kazakhstan).

##### Published (original) locality.

Kazakhstan: “between Keles [River] and Kosaral [Lake]”, Kyzyl-Kum [desert]; Uzbekistan: Urmitan [near Katty-Kurgan]; Tajikistan: Varzaminor [Ayni], near Obburden [Obburdon].

##### Lectotype.

♀, designated by by Osytshnjuk et al. 2005: 144, 24.[VI.1871] // Косаралъ [Kazakhstan, “Kosaral Lake” (now Shardara Reservoir), ≈ 41°10'N, 68°06'E] // *Andrenalateralis* Mor. [handwritten by F. Morawitz] // Lectotypus Warncke 1975 <red label> // Lectotypus *Andrenalateralis* Morawitz, 1876., design. Osytshnjuk et al., 2005 <red label, labelled by Yu. Astafurova> [ZMMU].

**Figure 22.**
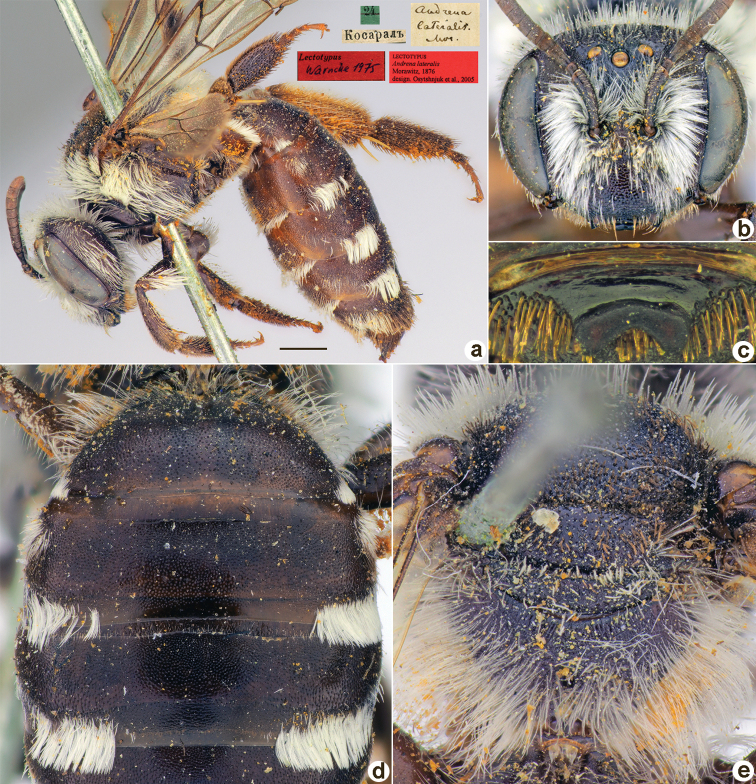
*Andrenalateralis* Morawitz, 1876, lectotype, female **A** habitus, lateral view and labels **B** head, frontal view **C** labrum, dorsal view **D** T1–T3, dorsal view **E** mesosoma, dorsal view. Scale bar: 1.0 mm.

##### Paralectotypes

**(16 ♀, 12 ♂).** 1 ♀, 24.[VI.1871] // Косаралъ // к.[оллекция] Ф. Моравица [Collection of F. Morawitz] // *Andrenalateralis* F.Mor. Popov 1935 det.; 1 ♀, 1 ♂, <golden circle>, 24.[VI.1871] // Косаралъ // *lateralis* Mor. Typ. [handwritten by F. Morawitz]; 2 ♂, 25.[IV.1871] // Чардара [Shardara] // к.[оллекция] Ф. Моравица [Collection of F. Morawitz] // *Andrenalateralis* Mor. [handwritten by F. Morawitz] // Paralectotypus *Andrenalateralis* Mor., design. Osychnjuk, 1980 <identical red labels on each paralectotype specimen> [ZISP]; 4 ♀, 24.[IV.1871] // Косараль; 1 ♀, 30.[IV.1871] // Кизилкумъ [Kyzyl-Kum desert]; 9 ♀, Оббурденъ [Obburden]; 2 ♀, Варзаминоръ [Varzaminor]; 9 ♂, 25.[IV.1871] // Чардара // Lectotypus *Andrenalateralis* Morawitz, 1876, design. Osytshnjuk et al., 2005 <identical red labels on each paralectotype specimen, labelled by Yu. Astafurova> [ZMMU].

##### Current status.

*Andrena* (incertae sedis) *lateralis* Morawitz, 1876.

##### Remarks.

Osytshnjuk et al. (2005: 144) mistakenly mentioned sex of the lectotype as a male instead of female.

##### Distribution.

Europe, Russia (to East Siberia), Caucasus, Turkey, Israel, Iran, Afghanistan, Central Asia.

#### ﻿23. Andrena leucorhina

Taxon classificationAnimaliaHymenopteraAndrenidae

Morawitz, 1876

6D8B14C6-E3AC-5BD3-A0AC-A334A76E8EEE

Fig. 23

Andrena leucorhina Morawitz, 1876: 165 (key), 169, ♂.

##### Type locality.

Shardara (Kazakhstan).

##### Published (original) locality.

Kazakhstan: Syr-Darja River, near Chardara.

##### Lectotype (designated here).

♂, 25.[IV.1871] // Чардара [Kazakhstan, Shardara, 41°18'N, 67°57'E] // *Andrenaleucorhina* Mor. [handwritten by F. Morawitz] // Lectotypus Warmcke 1975 <red label> // Lectotypus *Andrenaleucorhina* Morawitz, 1876 design. Astafurova et al., 2022 <red label> [ZMMU].

##### Paralectotypes

**(2 ♂).** 2 ♂, 25.[IV.1871] // Чардара [Chardara] // к.[оллекция] Ф. Моравица [Collection of F. Morawitz] // *Andrenaleucorhina* Morawitz [handwritten by F. Morawitz] // Paralectotypus *Andrenaleucorhina* Morawitz, 1876 design. Astafurova et al., 2022 <red label> [1 ♂ – ZISP, 1 ♂ – ZMMU].

**Figure 23.**
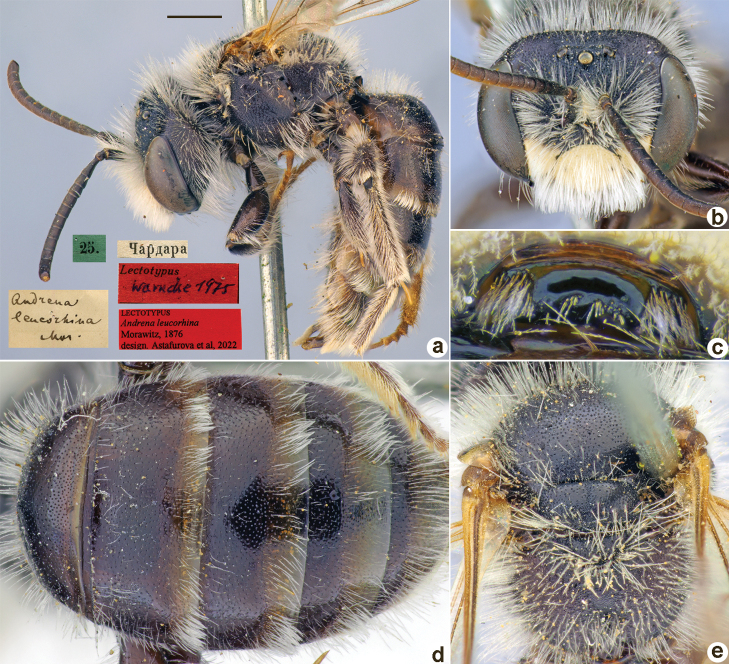
*Andrenaleucorhina* Morawitz, 1876, lectotype, male **A** habitus, lateral view and labels **B** head, frontal view **C** labrum, dorsal view **D** metasoma, dorsal view **E** mesosoma, dorsal view. Scale bar: 1.0 mm.

##### Current status.

Andrena (Ulandrena) abbreviata Dours, 1873 (according to Gusenleitner and Schwarz 2002).

##### Remarks.

Description of female: Morawitz 1876: 185 as *Andrenaravicollis* Morawitz, 1876 (synonymised by Osytshnjuk 1982: 28).

##### Distribution.

Southern Balkans, Cyprus, Turkey, Levant, Caucasus, Ukraine, Russia, Central Asia.

#### ﻿24. Andrena lucidicollis

Taxon classificationAnimaliaHymenopteraAndrenidae

Morawitz, 1876

341C135E-6C05-5F90-BACD-83421509E43E

Fig. 24

Andrena lucidicollis Morawitz, 1876: 165 (key), 181, ♂.

##### Type locality.

Tashkent (Uzbekistan).

##### Published (original) locality.

Uzbekistan: Tashkent.

##### Syntype.

♂, 5.[IV.1871] // Ташкентъ [Uzbekistan, Tashkent, 41°18'N, 69°16'E] // *Andrenalucidicollis* Mor. [handwritten by F. Morawitz] // Lectotypus Warncke, 1975 <red label> // Syntypus *Andrenalucidicollis* Mor., 1876 <red label> [ZMMU].

**Figure 24.**
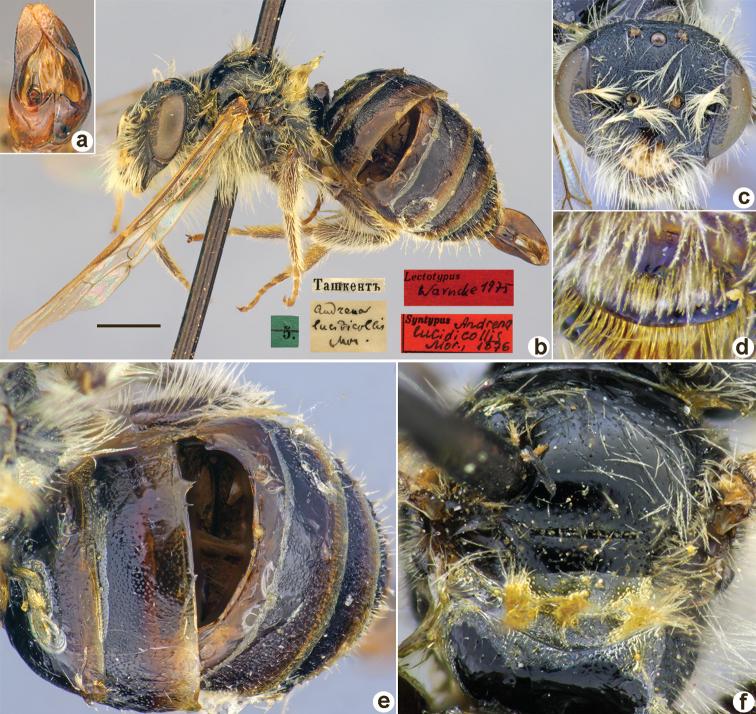
*Andrenalucidicollis* Morawitz, 1876, syntype, male **A** genitalia, dorsal view **B** habitus, lateral view and labels **C** head, frontal view **D** labrum, dorsal view **E** metasoma, dorsal view **F** mesosoma, dorsal view. Scale bar: 1.0 mm.

##### Current status.

Andrena (Poecilandrena) lucidicollis Morawitz, 1876

##### Remarks.

Female unknown (according to Gusenleitner and Schwarz 2002: 443).

Gusenleitner and Schwarz (2001: 140) refer to a “lectotype” deposited at the ZISP. However, we have found a single male specimen labelled “10.[IV.1871] // Ташкентъ // *Andrenalucidicollis* Mor. var. [handwritten by F. Morawitz] // Paralectotypus *Andrenalucidicollis* Mor., design. Osychnjuk, 1980”. This specimen does not belong to the type series formally, as it is labelled “10.[10 April 1871]” (in the original description Morawitz mentioned only specimens collected on 5 April). The syntype (Fig. 24) deposited at the ZMMU and labelled by Warncke as a “Lectotypus” on our opinion is in too poor a condition to be designated as a lectotype. However, both exemplars belong to one species and could be considered to be syntypes.

##### Distribution.

Uzbekistan.

#### ﻿25. Andrena maculipes

Taxon classificationAnimaliaHymenopteraAndrenidae

Morawitz, 1876

0AEAA303-FCD4-5360-A601-5590013F9B1D

Fig. 25

Andrena maculipes Morawitz, 1876: 162, 166 (key), 178, ♀, ♂.

##### Type locality.

Samarkand (Uzbekistan).

##### Published (original) locality.

Uzbekistan: near Samarkand.

##### Lectotype.

♂, designated by Osytshnjuk et al. 2005: 184, <golden circle>, 16.[III.1869] // Самаркандъ [Uzbekistan, Samarkand, 39°39'N, 66°57'E] // *maculipes* Mor. Typ. [handwritten by F. Morawitz] // Lectotypus *Andrenamaculipes* Mor., design. Osychnjuk, 1980 <red label> // Lectotypus *Andrenamaculipes* Morawitz, 1876, design. Osytshnjuk et al., 2005 <red label, labelled by Yu. Astafurova> // Zoological Institute St. Petersburg INS_HYM_0000187 [ZISP].

**Figure 25.**
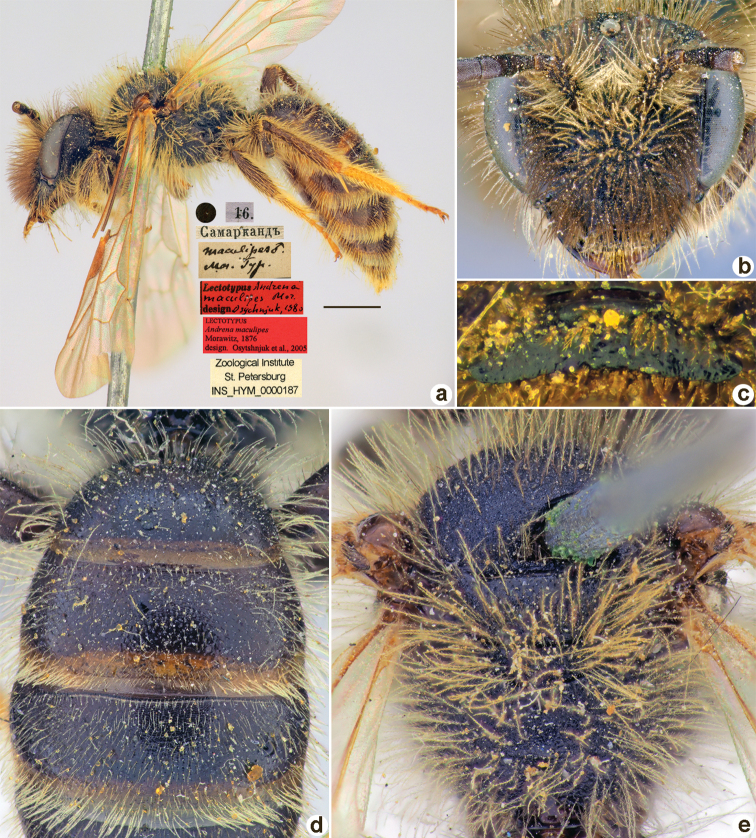
*Andrenamaculipes* Morawitz, 1876, lectotype, male **A** habitus, lateral view and labels **B** head, frontal view **C** labrum, dorsal view **D** T1–T3, dorsal view **E** mesosoma, dorsal view. Scale bar: 1.0 mm.

##### Paralectotypes

**(12 ♀, 2 ♂).** 2 ♀, 2 ♂, Самаркандъ // к.[оллекция] Ф. Моравица [Collection of F. Morawitz] // *Andrenamaculipes* F. Moraw. [handwritten by F. Morawitz]; 1 ♀, <golden circle>, 16.[III.1869] // Самаркандъ // *maculipes* Mor. Typ. [handwritten by F. Morawitz] // Paralectotypus *Andr.maculipes* Mor., design. Osychnjuk, 1980 <identical red labels on each paralectotype specimen> [ZISP]; 7 ♀, 7, 16.[III.1869] // Самаркандъ; 2 ♀, 4.[III.1869] // Заравш.[анская] дол.[ина] [Zaravshan River Valley] // Paralectotypus *Andrenamaculipes* Morawitz, 1876, design. Osytshnjuk et al., 2005 <identical red labels on each paralectotype specimen, labelled by Yu. Astafurova> [ZMMU].

##### Current status.

Andrena (Chrysandrena) maculipes Morawitz, 1876.

##### Remarks.

Osytshnjuk et al. (2005: 184) mistakenly mentioned the lectotype depository as the ZMMU instead of the ZISP.

##### Distribution.

Central Asia.

#### ﻿26. Andrena majalis

Taxon classificationAnimaliaHymenopteraAndrenidae

Morawitz, 1876

AFACB171-0C0A-54F1-AC08-46299ECAF5A7

Fig. 26

Andrena majalis Morawitz, 1876: 163 (key), 182, ♀.

##### Type locality.

Bairkum (Kazakhstan).

##### Published (original) locality.

Kazakhstan: Bairakum, Karak steppe; Uzbekistan: Zeravshan River valley, Dzham Gorge, Oalyk Gorge between Oalyk and Aksay.

##### Lectotype.

♀, designated by Osytshnjuk et al. 2008: 48, 4.[V.1871] // Байракумъ [Kazakhstan, Bairkum, Syr-Darya River, 42°05'N, 68°10'E] // *Andrenamajalis* Mor. [handwritten by F. Morawitz] // Lectotypus Warmcke, 1975 <red label> // Lectotypus *Andrenamajalis* Morawitz, 1876., design. Osytshnjuk et al., 2005 <red label, labelled by Yu. Astafurova> [ZMMU].

**Figure 26.**
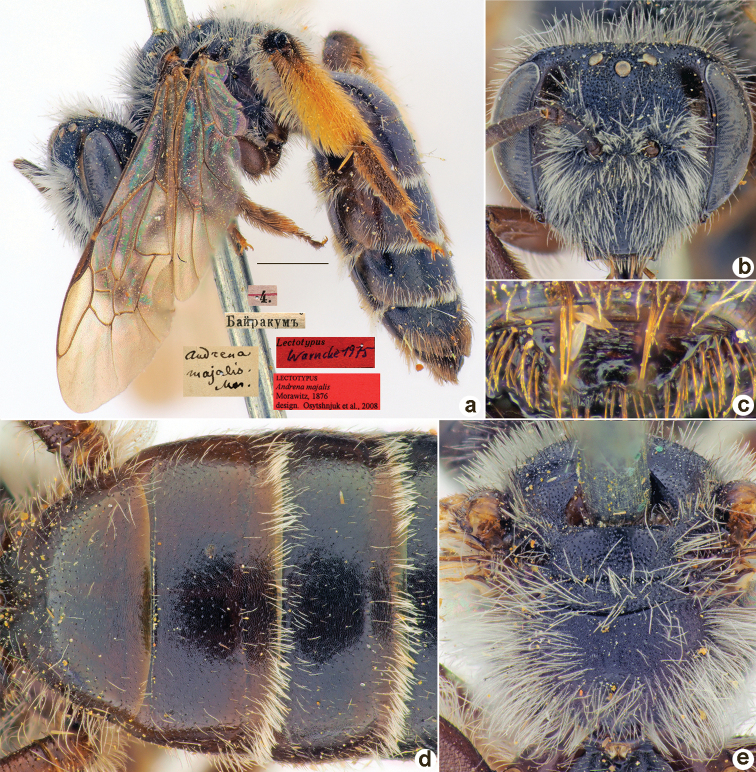
*Andrenamajalis* Morawitz, 1876, lectotype, female **A** habitus, lateral view and labels **B** head, frontal view **C** labrum, dorsal view **D** T1–T3, dorsal view **E** mesosoma, dorsal view. Scale bar: 1.0 mm.

##### Paralectotypes

**(14 ♀).** 3 ♀, the same labels as in the lectotype; 3 ♀, 5.[V.1871] // Каракск.[ая] степь [Karak steppe] // к.[оллекция] Ф. Моравица [Collection of F. Morawitz]// Paralectotypus *Andr.majalis* Mor., design. Osychnjuk, 1980 <identical red labels on each paralectotype specimen> [ZISP]; 4 ♀, 4.[V.1871] // Байракумъ; 1 ♀, 5.[V.1871] // Каракск.[ая] степь; 3 ♀, 16.,17.[V.1869] // Заравш.[анская] дол.[ина] [Zaravshan River Valley] // Paralectotypus *Andrenamajalis* Morawitz, 1876 design. Osychnjuk et al., 2008 <identical red labels on each paralectotype specimen, labelled by Yu. Astafurova> [ZMMU].

##### Current status.

Andrena (Euandrena) majalis Morawitz, 1876.

##### Remarks.

Description of male: Shebl and Tadauchi 2009: 49, figs 23, 24.

##### Distribution.

Central Asia.

#### ﻿27. Andrena mordax

Taxon classificationAnimaliaHymenopteraAndrenidae

Morawitz, 1876

055E3059-8E4C-54CB-BF56-36294DB2D4EE

Fig. 27

Andrena mordax Morawitz, 1876: 165 (key), 196, ♂.

##### Type locality.

Ayni (Tajikistan).

##### Published (original) locality.

Tajikistan: Varzaminor.

##### Lectotype.

♂, designated by Osytshnjuk et al. 2008: 122, 8.[VI.1870] // Варзаминоръ [Tajikistan, Varzaminor (= Ayni), 39°23'N, 68°32'E] // *Andrenamordax* Mor. [handwritten by F. Morawitz] // Lectotypus Warmcke 1975 <red label> // Lectotypus *Andrenamordax* Morawitz, 1876, design. Osytshnjuk et al., 2008 <red label, labelled by Yu. Astafurova> [ZMMU].

**Figure 27.**
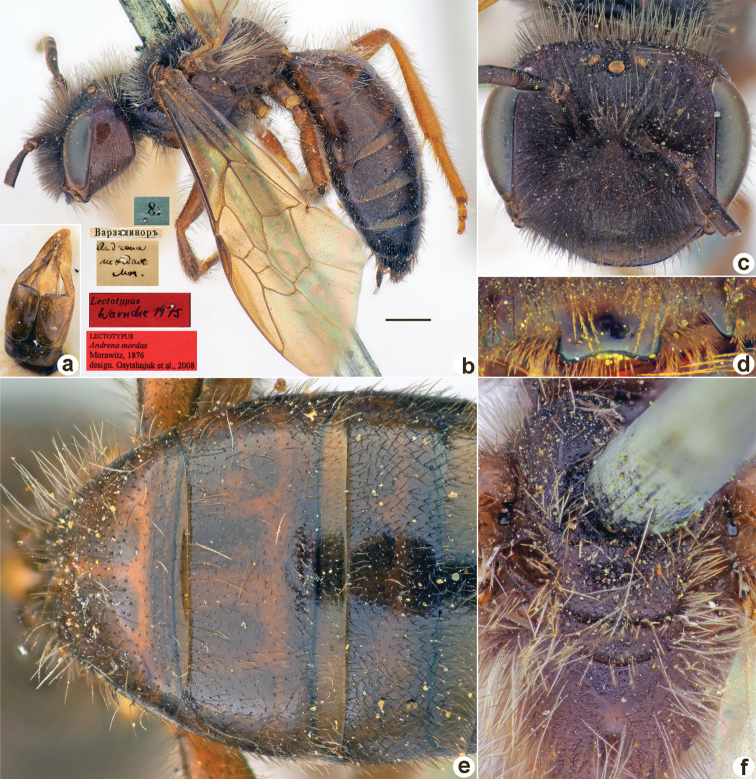
*Andrenamordax* Morawitz, 1876, lectotype, male **A** genitalia, dorsal view **B** habitus, lateral view and labels **C** head, frontal view **D** labrum, dorsal view **E** T1–T3, dorsal view **F** mesosoma, dorsal view. Scale bar: 1.0 mm.

##### Paralectotypes

**(3 ♂).** 2 ♂, 8.[VI.1870] // Варзаминоръ // *Andrenamordax* Mor. [handwritten by F. Morawitz] // Paralectotypus *Andrenamordax* Mor., design. Osychnjuk, 1980 <red label> [ZISP]; 1 ♂, 9.[VI.1870] // Варзаминоръ // Paralectotypus *Andrenamordax* Morawitz, 1876, design. Osytshnjuk et al., 2008 <red label, labelled by Yu. Astafurova> [ZMMU].

##### Current status.

Andrena (Hoplandrena) mordax Morawitz, 1876.

##### Remarks.

Description of female: Morawitz 1880: 362.

##### Distribution.

Uzbekistan, Tajikistan, Kyrgyzstan, Kazakhstan, China.

#### ﻿28. Andrena mucorea

Taxon classificationAnimaliaHymenopteraAndrenidae

Morawitz, 1876

5A85590B-42AF-594E-9EFF-0A4FAA607D96

Fig. 28

Andrena mucorea Morawitz, 1876: 164, 165 (key), 212, ♀, ♂.

##### Type locality.

Between Tashkent and Keless River (Uzbekistan).

##### Published (original) locality.

Uzbekistan: between Tashkent and Keless River, Urmitan [near Katty-Kurgan]; Kazakhstan: “between Keles [River] and Kosaral [Lake]”, Kyzyl Kum [desert].

##### Lectotype (designated here).

♀, 23.[IV.1871] // Келесъ [River] [Uzbekistan, between Tashkent and Keless River, ≈ 41°15'N, 69°10'E] // *Andrenamucorea* Mor. [handwritten by F. Morawitz] // к.[оллекция] Ф. Моравица [Collection of F. Morawitz] // Paralectotypus *Andr.mucorea* Mor., design. Osychnjuk, 1980 <red label> // Lectotypus *Andrenamucorea* Morawitz, 1876 design. Astafurova et al., 2022 <red label> // Zoological Institute St. Petersburg INS_HYM_0000294 [ZMMU].

**Figure 28.**
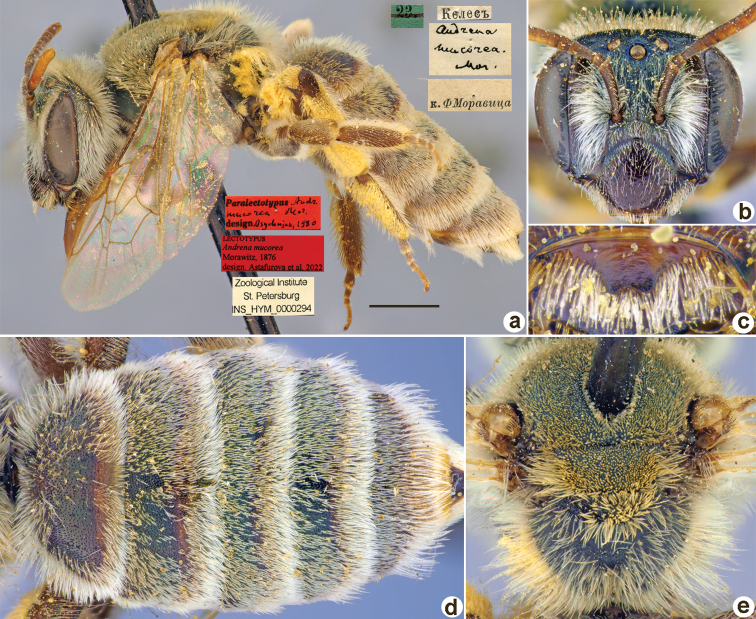
*Andrenamucorea* Morawitz, 1876, lectotype, female **A** habitus, lateral view and labels **B** head, frontal view **C** labrum, dorsal view **D** metasoma, dorsal view **E** mesosoma, dorsal view. Scale bar: 1.0 mm.

##### Paralectotypes

**(12 ♀, 1 ♂).** 1 ♀, 1 ♂, 22., 23.[IV.1871] // Келесъ // к.[оллекция] Ф. Моравица [Collection of F. Morawitz] // *Andrenamucorea* Mor. [handwritten by F. Morawitz]; 1 ♀, 24.IV.1871 // Косаралъ [Kosaral] // к.[оллекция] Ф. Моравица [Collection of F. Morawitz]; 1 ♀, Косаралъ // к.[оллекция] Ф. Моравица [Collection of F. Morawitz] // *mucorea* F. Mor., ♀ [handwritten by F. Morawitz] // Paralectotypus *Andr.mucorea* Mor., design. Osychnjuk, 1980 <identical red labels on each paralectotype specimen > [ZISP]; 1 ♀, 10.[V.1869] // Урмитанъ [Urmitan] // *Andrenamucorea* Mor. [handwritten by F. Morawitz] // Lectotypus Warncke 1975 <red label>; 7 ♀, 22., 23.[IV.1871] // Келесъ; 2♀, 9., 10.[V.1869] // Урмитанъ // Paralectotypus *Andrenamucorea* Morawitz, 1876, design. Astafurova et al., 2022 <identical red labels on each paralectotype specimen> [ZMMU].

##### Current status.

Andrena (Poecilandrena) mucorea Morawitz, 1876.

##### Distribution.

Uzbekistan, Tajikistan, Kazakhstan.

#### ﻿29. Andrena nigrita

Taxon classificationAnimaliaHymenopteraAndrenidae

Morawitz, 1876

91DEE2B8-6FED-5FB1-9E67-2823415B3F1F

Fig. 29

Andrena nigrita Morawitz, 1876: 166 (key), 196, ♂.

##### Type locality.

Iskanderkul Lake (Tajikistan).

##### Published (original) locality.

Tajikistan: Iskanderkul Lake.

##### Holotype.

♂, 18.[VI.1870] // Искандеръ [Tajikistan, Iskanderkul Lake, Hissar Ridge, 39°04'N, 68°22'E] // *Andrenanigrita* Mor. [handwritten by F. Morawitz] // Lectotypus Warmcke 1975 <red label> // Holotypus *Andrenanigrita* Mor., 1876 <red label, labelled by Yu. Astafurova> [ZMMU].

**Figure 29.**
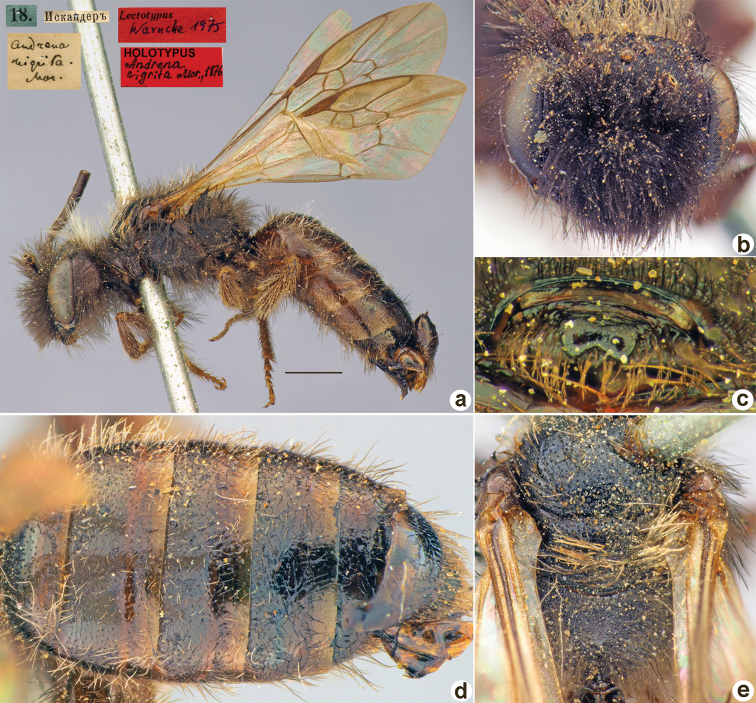
*Andrenanigrita* Morawitz, 1876, holotype, male **A** habitus, lateral view and labels **B** head, frontal view **C** labrum, dorsal view **D** metasoma, dorsal view **E** mesosoma, dorsal view. Scale bar: 1.0 mm.

##### Current status.

Andrena (Euandrena) nigritula Cockerell, 1906 [replacement name by Cockerell 1906: 74 (nec *Andrenanigrita* Fabricius, 1775; nec *Nomadanigrita* Panzer, 1800)].

##### Remarks.

Description of female: Morawitz 1893: 69.

The lectotype designation by Osytshnjuk et al. (2008: 36) is unnecessary as the species was described from a single male that was directly written about by Morawitz (1876: 196).

##### Distribution.

Uzbekistan, Tajikistan, Kyrgyzstan, Kazakhstan.

#### ﻿30. Andrena nitidicollis

Taxon classificationAnimaliaHymenopteraAndrenidae

Morawitz, 1876

6C0500DB-E21A-586A-B461-FBC4277C16E2

Fig. 30

Andrena nitidicollis Morawitz, 1876: 162, 166 (key), 180, ♀, ♂.

##### Type locality.

Kyzylkum Desert (Kazakhstan).

##### Published (original) locality.

Kazakhstan: Kyzyl-kum Desert; Karak mountains [western part of Kysyl-kum, NW Bairkum].

##### Lectotype (designated here).

♀, 29.[IV.1871] // Кизилъкумъ [Kazakhstan, Kyzylkum Desert, NW of Shardara, ≈ 41°58'N, 67°03'E] // *Andrenanitidicollis* Mor. [handwritten by F. Morawitz] // Lectotypus Warncke 1975 <red label> // Lectotypus *Andrenanitidicollis* Morawitz, 1876, design. Astafurova et al., 2022 <red label> [ZMMU].

**Figure 30.**
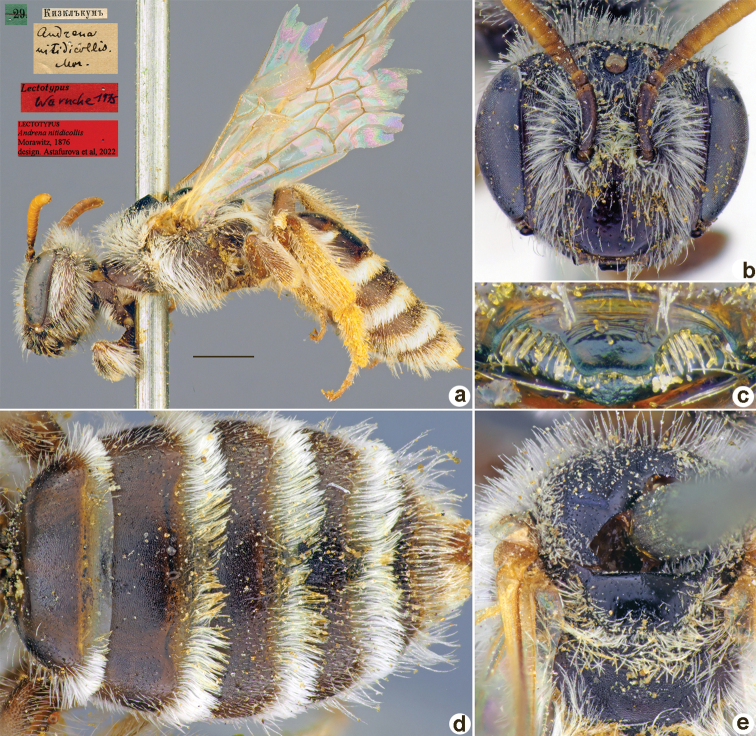
*Andrenanitidicollis* Morawitz, 1876, lectotype, female **A** habitus, lateral view and labels **B** head, frontal view **C** labrum, dorsal view **D** metasoma, dorsal view **E** mesosoma, dorsal view. Scale bar: 1.0 mm.

##### Paralectotype.

1 ♂, 6.[V.1871] // Кизилъкумъ // Paralectotypus *Andrenanitidicollis* Morawitz, 1876, design. Astafurova et al., 2022 <red label> [ZMMU].

##### Current status.

*Andrena* (incertae sedis) *nitidicollis* Morawitz, 1876.

##### Distribution.

Kazakhstan.

#### ﻿31. Andrena nupta

Taxon classificationAnimaliaHymenopteraAndrenidae

Morawitz, 1876

1D4DCA7C-1F1C-5485-AD49-3D7F45537F8A

Fig. 31

Andrena nupta Morawitz, 1876: 163, 166 (key), 191, ♀, ♂.

##### Type locality.

Tashkent (Uzbekistan).

##### Published (original) locality.

Uzbekistan: Tashkent and Samarkand.

##### Lectotype.

♀, designated by Osytshnjuk et al. 2008: 46, 8.[IV.1871] // Ташкентъ [Uzbekistan, Tashkent, 41°18'N, 69°16'E] // *Andrenanupta* Mor. [handwritten by F. Morawitz] // Lectotypus Warncke 1975 <red label> // Lectotypus *Andrenanupta* Morawitz, 1876, design. Osytshnjuk et al., 2008 <red label, labelled by Yu. Astafurova> [ZMMU].

**Figure 31.**
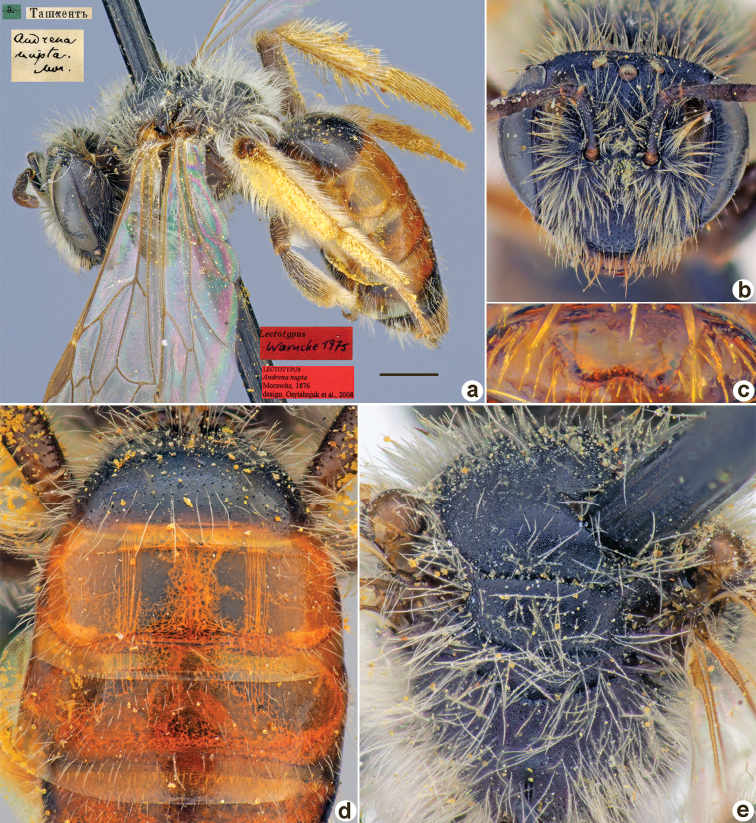
*Andrenanupta* Morawitz, 1876, lectotype, female **A** habitus, lateral view and labels **B** head, frontal view **C** labrum, dorsal view **D** T1–T3, dorsal view **E** mesosoma, dorsal view. Scale bar: 1.0 mm.

##### Paralectotypes

**(5 ♀, 4 ♂).** 1 ♀, the same label as in the lectotype; 2 ♀, 2 ♂, Ташкентъ // к.[оллекция] Ф. Моравица [Collection of F. Morawitz] // *nupta* F. Mor. [handwritten by F. Morawitz] // Paralectotypus *Andr.nupta* Mor., design. Osychnjuk, 1980 <identical red labels on each paralectotype specimen> [ZISP]; 2 ♀, 1 ♂, 27.[III.1871], 3., 8.[IV.1871] // Ташкентъ; 1 ♂, 5.[IV.1869] // Самаркандъ [Samarkand] // Paralectotypus *Andrenanupta* Morawitz, 1876, design. Osytshnjuk et al., 2008 <identical red labels on each paralectotype specimen, labelled by Yu. Astafurova> [ZMMU].

##### Current status.

Andrena (Euandrena) nupta Morawitz, 1876.

##### Distribution.

Uzbekistan, Kazakhstan.

#### ﻿32. Andrena oralis

Taxon classificationAnimaliaHymenopteraAndrenidae

Morawitz, 1876

9B29B2C9-3AE3-56A6-B52E-07F70DB32B87

Fig. 32

Andrena oralis Morawitz, 1876: 162 (key), 177, ♀.

##### Type locality.

Tashkent (Uzbekistan).

##### Published (original) locality.

Uzbekistan: near Tashkent.

##### Lectotype (designated here).

♀, <golden circle> // Ташкентъ [Uzbekistan, Tashkent, 41°18'N, 69°16'E] // *oralis* Mor. Typ. [handwritten by F. Morawitz] // Lectotypus *Andrenaoralis* Mor., design. Osychnjuk, 1980 <red label> // Lectotypus, *Andrenaoralis* Morawitz, 1876, design. Astafurova et al., 2022 <red label> // Zoological Institute St. Petersburg INS_HYM_0000201 [ZISP].

**Figure 32.**
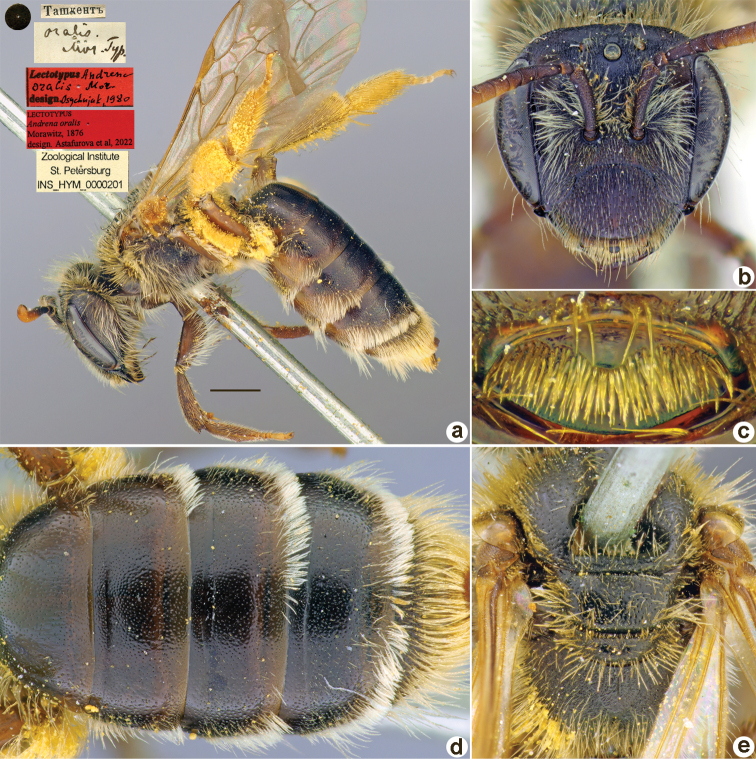
*Andrenaoralis* Morawitz, 1876, lectotype, female **A** habitus, lateral view and labels **B** head, frontal view **C** labrum, dorsal view **D** metasoma, dorsal view **E** mesosoma, dorsal view. Scale bar: 1.0 mm.

##### Paraectotype.

1 ♀, 5.[IV.1871] // Ташкент // *Andrenaoralis* Mor. [handwritten by F. Morawitz] // Lectotypus Warncke 1975 <red label> // Paralectotypus *Andrenaoralis* Morawitz, 1876, design. Astafurova et al., 2022 <red label> [ZMMU].

##### Current status.

Andrena (Orandrena) oralis Morawitz, 1876.

##### Remarks.

Description of male: Morawitz 1876: 177, as *Andrenasogdiana* Morawitz, 1876 (according to Gusenleitner and Schwarz 2001: 150).

##### Distribution.

Europe, Russia (to the Urals), Turkey, Turkmenistan, Uzbekistan, Kazakhstan.

#### ﻿33. Andrena pannosa

Taxon classificationAnimaliaHymenopteraAndrenidae

Morawitz, 1876

9532633F-6932-54FE-9D00-E3F908AAA849

Fig. 33

Andrena pannosa Morawitz, 1876: 162 (key), 197, ♀.

##### Type locality.

Khodzha-Chiburgan Gorge (Kyrgyzstan).

##### Published (original) locality.

Kyrgyzstan: Khodzha-Chiburgan Gorge.

##### Lectotype.

♀, <golden circle>, 21.[VI.1871] // Чибурганъ [Kyrgyzstan, Khodzha-Chiburgan Gorge, S Varuch, ≈ 39°48'N, 70°41'E] // *pannosa* Mor. Typ. [handwritten by F. Morawitz] // Lectotypus *Andrenapannosa* Mor., design. Osychnjuk, 1980 <red label> // Lectotypus, *Andrenapannosa* Morawitz, 1876 design. Astafurova et al., 2022 <red label> // Zoological Institute St. Petersburg INS_HYM_0000287 [ZISP].

**Figure 33.**
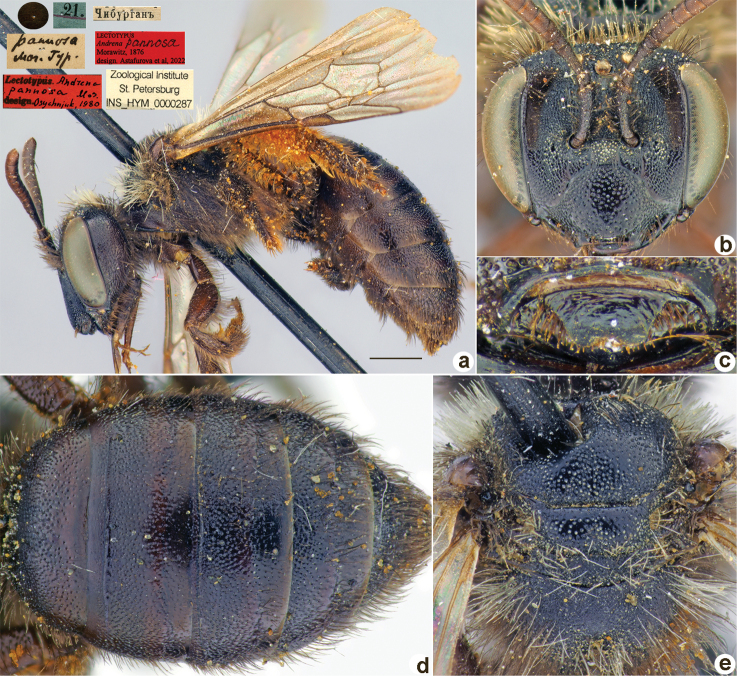
*Andrenapannosa* Morawitz, 1876, lectotype, female **A** habitus, lateral view and labels **B** head, frontal view **C** labrum, dorsal view **D** metasoma, dorsal view **E** mesosoma, dorsal view. Scale bar: 1.0 mm.

##### Paralectotypes

**(2 ♀).** 1 ♀, 21.[VI.1871] // Чибурганъ // к.[оллекция] Ф. Моравица [Collection of F. Morawitz] // *Andrenapannosa* Mor. [handwritten by F. Morawitz] // Paralectotypus *Andr.pannosa* Mor., design. Osychnjuk, 1980 <red label> [ZISP]; 1 ♀, 21.[VI.1871] // Чибурганъ // Paralectotypus *Andrenapannosa* Morawitz, 1876, design. Astafurova et al., 2022 <identical red labels on each paralectotype specimen> [ZMMU].

##### Current status.

Andrena (Euandrena) pannosa Morawitz, 1876.

##### Remarks.

Description of male: Gusenleitner and Schwarz 2001: 153. The lectotype was mistakenly designated by Osytshnjuk et al. (2008: 54) in ZMMU. This specimen is a female labelled “21.[06.1971] // Soch [Uzbek enclave] *Andrenapannosa* Mor. [handwritten by F. Morawitz] // Lectotypus Warncke 1975.” However, according to the original description (Morawitz, 1876), the type location is “Khodzha-Chiburgan Gorge” [Kyrgyzstan, S Varuch]; thus, the lectotype published by Osytshnjuk et al. (2008) is not valid. There are two females in ZISP from “Chiburgan”, which correspond to the original description of Morawitz. One of these females labelled by A. Osytshnjuk as “lectotype” is designated here as a lectotype of *Andrenapannosa*.

##### Distribution.

Uzbekistan, Kyrgyzstan, Tajikistan, Kazakhstan.

#### ﻿34. Andrena planirostris

Taxon classificationAnimaliaHymenopteraAndrenidae

Morawitz, 1876

B23B5039-ED84-5D62-A6C3-AB2075AB4F9B

Fig. 34

Andrena planirostris Morawitz, 1876: 163, 165 (key), 174, ♀, ♂.

##### Type locality.

Tashkent (Uzbekistan).

##### Published (original) locality.

Uzbekistan: near Tashkent.

##### Lectotype (designated here).

♀, 23.[III.1871] // Ташкентъ [Uzbekistan, Tashkent, 41°18'N, 69°16'E] // *Andrenaplanirostris* Mor. [handwritten by F. Morawitz] // к.[оллекция] Ф. Моравица [Collection of F. Morawitz] // Paralectotypus *Andr.planirostris* Mor., design. Osychnjuk, 1980 <red label> // Lectotypus *Andrenaplanirostris* Morawitz, 1876, design. Astafurova et al., 2022 <red label> // Zoological Institute St. Petersburg INS_HYM_0000256 [ZISP].

**Figure 34.**
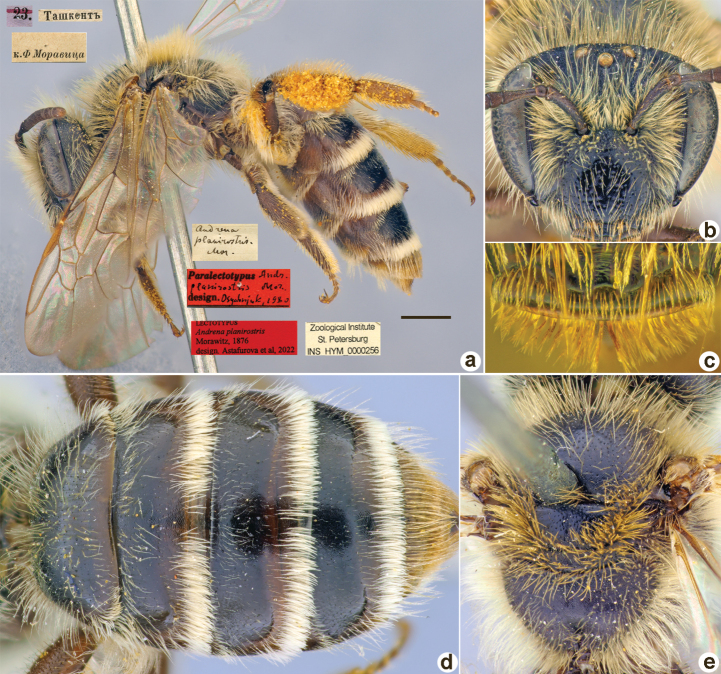
*Andrenaplanirostris* Morawitz, 1876, lectotype, female **A** habitus, lateral view and labels **B** head, frontal view **C** labrum, dorsal view **D** metasoma, dorsal view **E** mesosoma, dorsal view. Scale bar: 1.0 mm.

##### Paralectotypes

**(20 ♀, 2 ♂).** 1 ♀, <golden circle> // 25.[III.1871] // Ташкентъ // *planirostris* Mor. Typ. ♀ [handwritten by F. Morawitz]; 9 ♀, 23., 25.[III.1871] // Ташкентъ // к.[оллекция] Ф. Моравица [Collection of F. Morawitz]; 1 ♂, Ташкентъ // *planirostris* Mor. [handwritten by F. Morawitz] // Paralectotypus *Andr.planirostris* Mor., design. Osychnjuk, 1980 <red label> [ZISP]; 10 ♀, 1 ♂, 11., 22., 23., 24., 25., 26. Ташкентъ // Paralectotypus *Andrenaplanirostris* Morawitz, 1876, design. Astafurova et al., 2022 <identical red labels on each paralectotype specimen> [ZMMU].

##### Remarks.

There is a female specimen labelled by Warcke as a “Lectotypus” [<golden circle> // 5.[IV.1871] // Ташкент // *Andrenaplanirostris* Mor. [handwritten by F. Morawitz] // Lectotypus Warncke 1975; ZMMU]. However, the label date does not correspond to any date [11–26.III.1871) mentioned for the type series by Morawitz (1876: 174).

##### Current status.

Andrena (Planiandrena) planirostris Morawitz, 1876.

##### Distribution.

Uzbekistan, Kazakhstan.

#### ﻿35. Andrena punctifrons

Taxon classificationAnimaliaHymenopteraAndrenidae

Morawitz, 1876

42502708-0CC4-593B-85A3-AEC547D2C9AD

Fig. 35

Andrena punctifrons Morawitz, 1876: 164 (key), 202, ♀.

##### Type locality.

Khodzha-Chiburgan River (Kyrgyzstan).

##### Published (original) locality.

Kyrgyzstan: “Khodzha-Chiburgan River”.

##### Holotype.

♀, Чибурганъ [Kyrgyzstan: Khodzha-Chiburgan River/Gorge, S Varuch, ≈ 39°48'N, 70°41'E] // *Andrenapunctifrons* Mor. [handwritten by F. Morawitz] // Lectotypus Warncke 1975 <red label> // Holotypus *Andrenapunctifrons* Mor., 1876 <red label> [ZMMU].

**Figure 35.**
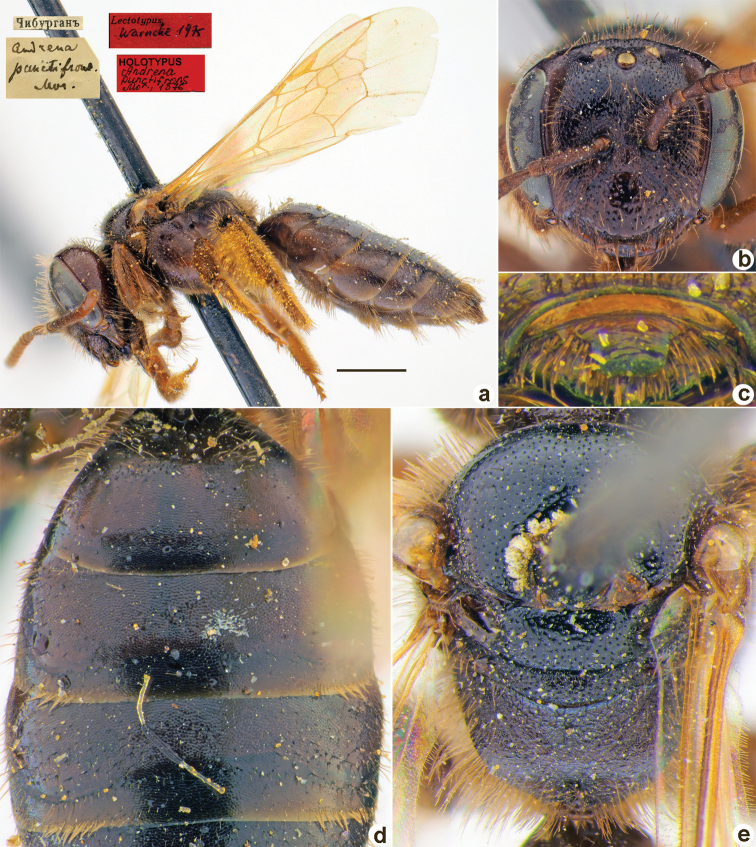
*Andrenapunctifrons* Morawitz, 1876, holotype, female **A** habitus, lateral view and labels **B** head, frontal view **C** labrum, dorsal view **D** T1–T3, dorsal view **E** mesosoma, dorsal view. Scale bar: 1.0 mm.

##### Current status.

Andrena (Micrandrena) punctifrons Morawitz, 1876.

##### Remarks.

Listed as *Graecandrena* by Osytshnjuk et al. (2008: 93). Male unknown (according to Osytshnjuk et al. 2008: 93).

##### Distribution.

Kyrgyzstan.

#### ﻿36. Andrena punctiventris

Taxon classificationAnimaliaHymenopteraAndrenidae

Morawitz, 1876

9252B04B-2569-5D89-8F09-732E73B8D1B8

Fig. 36

Andrena punctiventris Morawitz, 1876: 162 (key), 186, ♀.

##### Type locality.

Iskanderkul Lake (Tajikistan).

##### Published (original) locality.

Tajikistan: near Iskander-Kul Lake.

##### Holotype.

♀, 15.[VI.1870] // Искандеръ [Tajikistan, Iskanderkul Lake, Hissar Ridge, 39°04'N, 68°22'E] // *Andrenapunctiventris* Mor. [handwritten by F. Morawitz] // Lectotypus Warncke 1975 <red label> // Holotypus *Andrenapunctiventris* Mor., 1876 <red label> [ZMMU].

**Figure 36.**
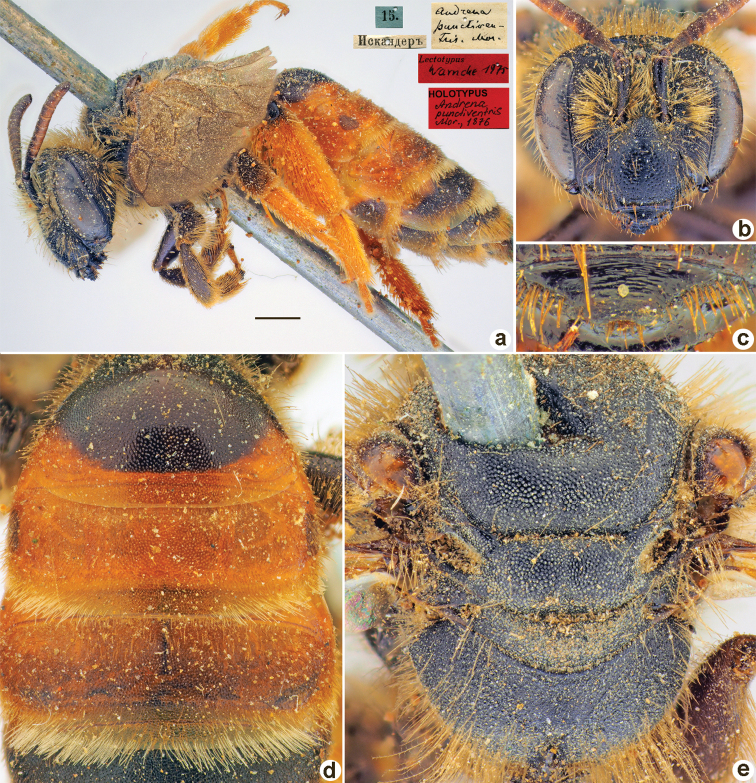
*Andrenapunctiventris* Morawitz, 1876, holotype, female **A** habitus, lateral view and labels **B** head, frontal view **C** labrum, dorsal view **D** T1–T3, dorsal view **E** mesosoma, dorsal view. Scale bar: 1.0 mm.

##### Current status.

Andrena (Lepidandrena) punctiventris Morawitz, 1876.

##### Remarks.

Listed as *Poliandrena* by Osytshnjuk (1994: 30). Description of male: Osytshnjuk 1994: 30, as Andrenapunctiventrisssp.basagiensis Osytshnjuk, 1994.

##### Distribution.

Uzbekistan, Tajikistan, Kazakhstan.

#### ﻿37. Andrena quadrifasciata

Taxon classificationAnimaliaHymenopteraAndrenidae

Morawitz, 1876

3DB49B1D-B095-50BA-878D-2362AF9DC8B7

Fig. 37

Andrena quadrifasciata Morawitz, 1876: 163 (key), 168, ♀.

##### Type locality.

Shakhimardan (Uzbekistan).

##### Published (original) locality.

Uzbekistan: near Shakhimardan.

##### Holotype.

♀, 7.[VII.1871] // Шагимарданъ [Shakhimardan in the Uzbek enclave in the territory of Kyrgyzstan, Alai Ridge, 39°58'N, 71°47'E] // *Andrenaquadrifasciata* Mor. [handwritten by F. Morawitz] // Lectotypus Warncke 1975 <red label> // Holotypus *Andrenaquadrifasciata* Mor., 1876 <red label> [ZMMU].

**Figure 37.**
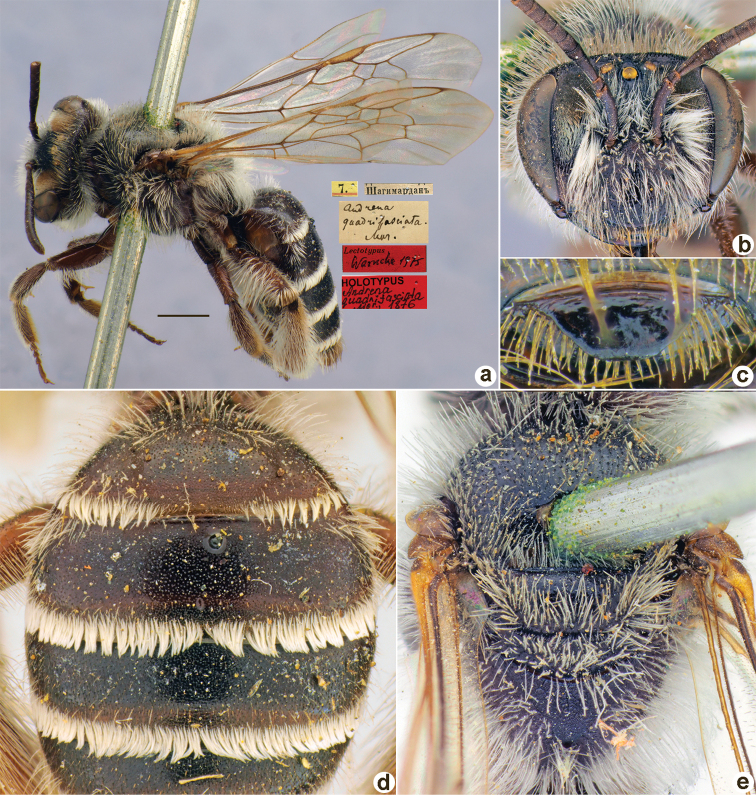
*Andrenaquadrifasciata* Morawitz, 1876, holotype, female **A** habitus, lateral view and labels **B** head, frontal view **C** labrum, dorsal view **D** T1–T3, dorsal view **E** mesosoma, dorsal view. Scale bar: 1.0 mm.

##### Current status.

Andrena (Simandrena) quadrifasciata Morawitz, 1876.

##### Remarks.

Description of male: Gusenleitner and Schwarz 2001: 157.

##### Distribution.

Tajikistan, Kazakhstan.

#### ﻿38. Andrena ravicollis

Taxon classificationAnimaliaHymenopteraAndrenidae

Morawitz, 1876

98834552-FE5D-5218-B35F-00F3F44B8B6B

Fig. 38

Andrena ravicollis Morawitz, 1876: 163 (key), 185, ♀.

##### Type locality.

Shardara (Kazakhstan).

##### Published (original) locality.

Kazakhstan: Chardara.

##### Lectotype (designated here).

♀, <golden circle> // 25.[IV.1871] // Чардара [Kazakhstan, Shardara, 41°18'N, 67°57'E] // *ravicollis* Mor. Typ. [handwritten by F. Morawitz] // Paralectotypus *Andrenaravicollis* Mor., design. Osychnjuk, 1980 <red label> // Lectotypus *Andrenaravicollis* Morawitz, 1876, design. Astafurova et al., 2022 <red label> // *Andrenaleucorhina*, D.A. Sidorov det., 2022 // Zoological Institute St. Petersburg INS_HYM_0000286 [ZISP].

**Figure 38.**
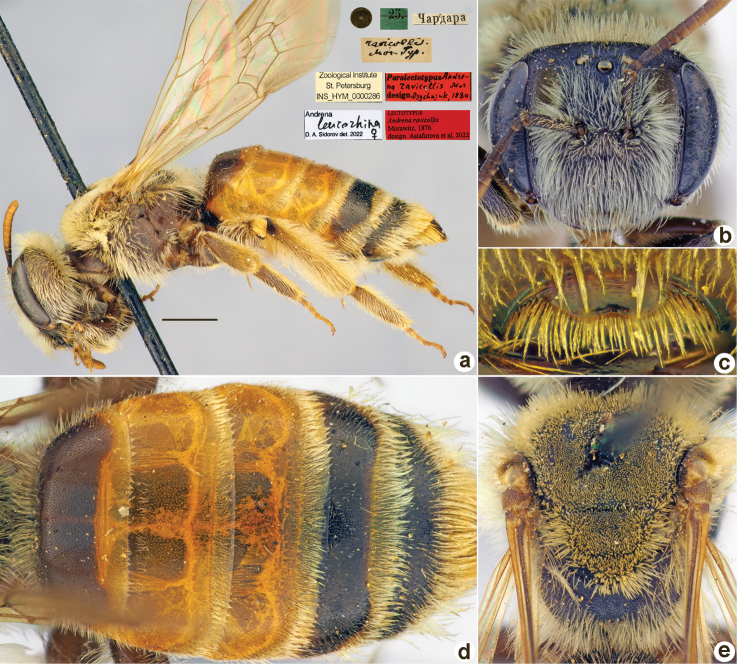
*Andrenaravicollis* Morawitz, 1876, lectotype, female **A** habitus, lateral view and labels **B** head, frontal view **C** labrum, dorsal view **D** metasoma, dorsal view **E** mesosoma, dorsal view. Scale bar: 1.0 mm.

##### Paralectotype.

1 ♀, 25.[IV.1871] // Чардара // *Andrenaleucorhina*, D.A. Sidorov det., 2022 // Paralectotypus *Andrenaravicollis* Morawitz, 1876, design. Astafurova et al., 2022 < red label> [ZMMU].

##### Remarks.

There is a female specimen (in bad condition) labelled by Warcke as a “Lectotypus” [27.[IV.1871] // Чардара // *Andrenaravicollis* Mor. [handwritten by F. Morawitz] // Lectotypus Warncke 1975; ZMMU]. However, the label date does not correspond the date [25.III.1871] mentioned for the type series by Morawitz (1876).

##### Current status.

Andrena (Ulandrena) abbreviata Dours, 1873 (according to Gusenleitner and Schwarz 2002).

##### Distribution.

Southern Balkans, Cyprus, Turkey, Levant, Caucasus, Ukraine, Russia, Central Asia.

#### ﻿39. Andrena rufilabris

Taxon classificationAnimaliaHymenopteraAndrenidae

Morawitz, 1876

29FB8B08-629A-5DB2-A68A-1EDC2E1D675D

Fig. 39

Andrena rufilabris Morawitz, 1876: 162 (key), 180, ♀.

##### Type locality.

NW of Bairkum (Kazakhstan).

##### Published (original) locality.

Uzbekistan: Katty-Kurgan; Kazakhstan: Karak steppe.

##### Lectotype (designated here).

♀, 5.[V.1871] // Каракск.[ая] степь [Kazakhstan, Karak steppe, NW of Bairkum, ≈ 42°46'N, 67°24'E] // *Andrenarufilabris* Mor. [handwritten by F. Morawitz] // Lectotypus Warncke 1975 <red label> // Lectotypus *Andrenarufilabris* Morawitz, 1876, design. Astafurova et al., 2022 <red label> [ZMMU].

**Figure 39.**
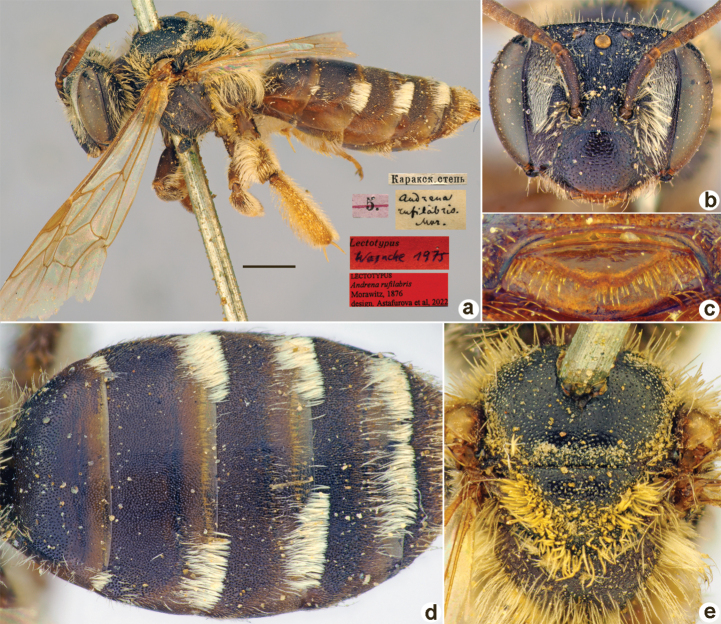
*Andrenarufilabris* Morawitz, 1876, lectotype, female **A** habitus, lateral view and labels **B** head, frontal view **C** labrum, dorsal view **D** metasoma, dorsal view **E** mesosoma, dorsal view. Scale bar: 1.0 mm.

##### Paralectotypes

**(2 ♀).** 2 ♀, 29.[VI.1869] and 6.[V.1869] // Катты Курганъ // *Andrenasatra*, D.A. Sidorov det., 2022 // Paralectotypus, *Andrenarufilabris* Morawitz, 1876, design. Astafurova et al., 2022 <red label> [ZMMU].

##### Current status.

Andrena (Simandrena) sarta Morawitz, 1876 (according to Gusenleitner and Schwarz 2002: 660).

##### Distribution.

Uzbekistan, Kazakhstan.

#### ﻿40. Andrena rufina

Taxon classificationAnimaliaHymenopteraAndrenidae

Morawitz, 1876

2698CE2C-0CD4-5A2A-89ED-33ADE9DA7DCA

Fig. 40

Andrena rufina Morawitz, 1876: 162, 165 (key), 167, ♀, ♂.

##### Type locality.

Anzob (Tajikistan).

##### Published (original) locality.

Tajikistan: Anzob.

##### Lectotype.

♂, designated by Osytshnjuk et al. 2005: 204, 20.VI.1870 // Ягнобъ [Tajikistan, Yagnob River near Anzob, 39°04'N, 68°52'E] // *Andrenarufina* Mor. [handwritten by F. Morawitz] // Lectotypus Warncke 1975 // Lectotypus *Andrenarufina* Morawitz, 1876, design. Osytshnjuk et al., 2005 <red label, labelled by Yu. Astafurova> [ZMMU].

**Figure 40.**
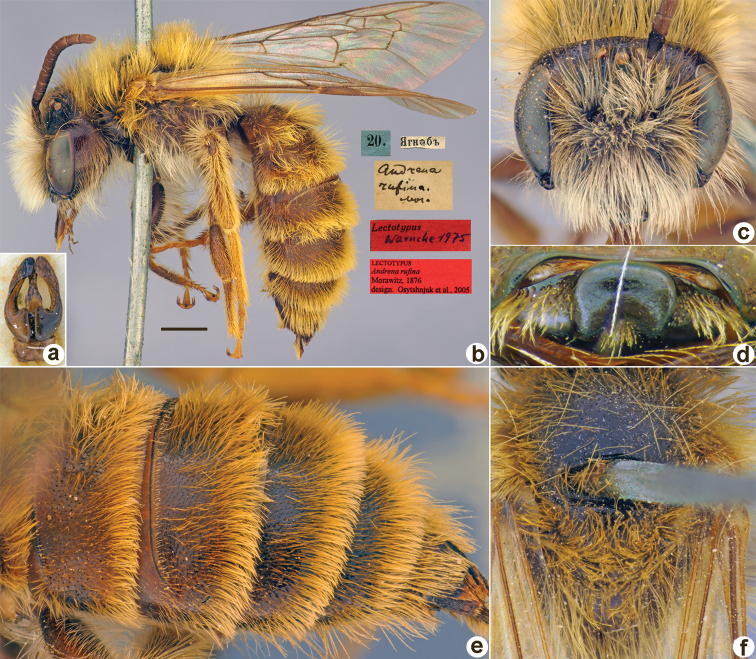
*Andrenarufina* Morawitz, 1876, lectotype, male **A** genitalia, dorsal view **B** habitus, lateral view and labels **C** head, frontal view **D** labrum, latero-dorsal view **E** metasoma, dorsal view **F** mesosoma, dorsal view. Scale bar: 1.0 mm.

##### Paralectotype.

♀, 75. // Туркест.[анский] кр.[ай] [Turkestan] // Paralectotypus, *Andrenarufina* Morawitz, 1876, design. Osytshnjuk et al., 2005 <red label, labelled by Yu. Astafurova> [ZMMU].

##### Current status.

Andrena (Cnemidandrena) rufina Morawitz, 1876.

##### Distribution.

Uzbekistan, Tajikistan, Kazakhstan.

#### ﻿41. Andrena sarta

Taxon classificationAnimaliaHymenopteraAndrenidae

Morawitz, 1876

4801D484-8040-534D-9125-A0496776FEA9

Fig. 41

Andrena sarta Morawitz, 1876: 164 (key), 171, ♀.

##### Type locality.

30 km SE of Kozhatogai, Turkistan Province (Kazakhstan).

##### Published (original) locality.

Kazakhstan: steppe between Tashkent and Syrdarya River.

##### Lectotype (designated here).

♀, 20.[V.1871] // Степь м.[ежду] С.[ыр] [-] д.[арьей] и Т.[ашкентом] [Kazakhstan, Turkistan Province, steppe between Syrdarya River and Tashkent, 30 km SE of Kozhatogai, 41°47'N, 68°23'E] // *satra* Mor. [handwritten by Osytshnjuk] // Lectotypus Warncke 1975 // Lectotypus *Andrenasarta* Morawitz, 1876, design. Astafurova et al., 2022 <red label> [ZMMU].

**Figure 41.**
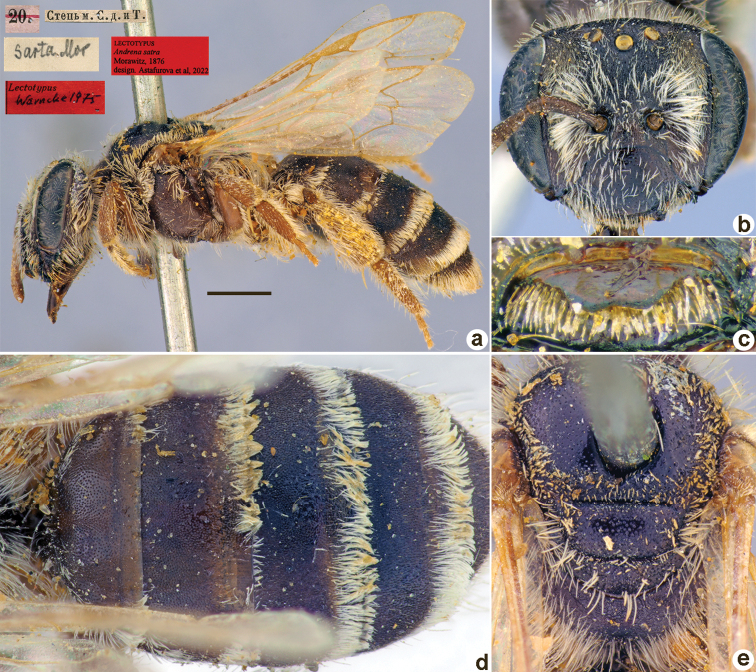
*Andrenasarta* Morawitz, 1876, lectotype, female **A** habitus, lateral view and labels **B** head, frontal view **C** labrum, dorsal view **D** metasoma, dorsal view **E** mesosoma, dorsal view. Scale bar: 1.0 mm.

##### Current status.

Andrena (Simandrena) sarta Morawitz, 1876.

##### Remarks.

Description of male: Gusenleitner and Schwarz 2001: 163.

##### Distribution.

Uzbekistan, Kazakhstan.

#### ﻿42. Andrena semiaenea

Taxon classificationAnimaliaHymenopteraAndrenidae

Morawitz, 1876

7EC68AB0-DB8B-50C4-B89A-0CF6054B5A10

Fig. 42

Andrena semiaenea Morawitz, 1876: 164 (key), 213, ♀.

##### Type locality.

Keles River, Turkistan Province (Kazakhstan).

##### Published (original) locality.

Kazakhstan: between Keles River and dry Keles; near Kosaral Lake; near Chardara.

##### Lectotype.

♀, designated by Osytshnjuk et al. 2008: 155, 23.[IV.1871] // Келесъ [Kazakhstan, Turkistan Province, Keles River] // *Andrenasemiaenea* Mor. [handwritten by F. Morawitz] // Lectotypus Warncke, 1975 // Lectotypus *Andrenasemiaenea* Morawitz, 1876, Osytshnjuk et al., 2008 <red label, labelled by Yu. Astafurova> [ZMMU].

**Figure 42.**
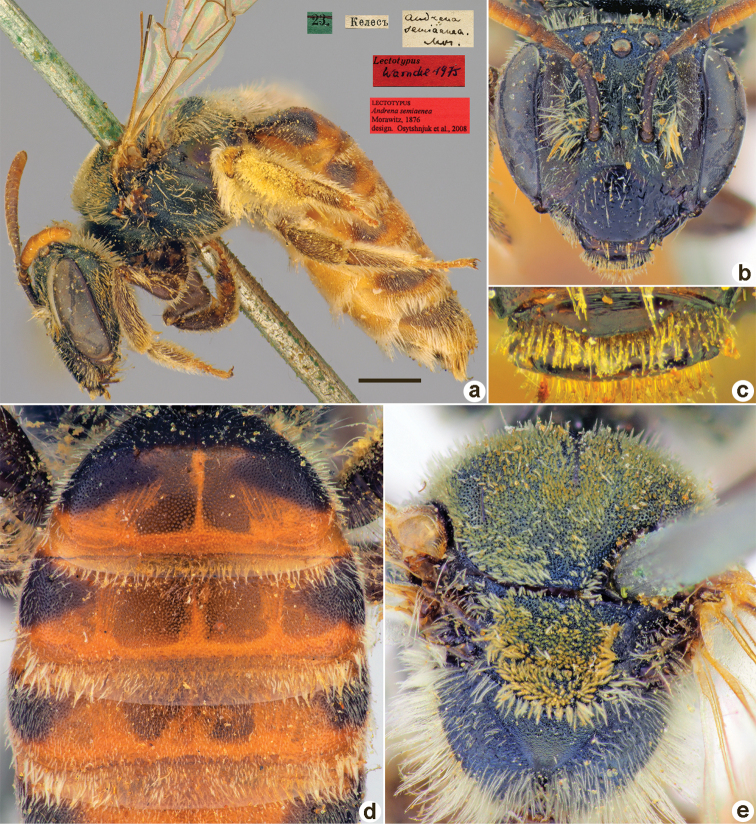
*Andrenasemiaenea* Morawitz, 1876, lectotype, female **A** habitus, lateral view and labels **B** head, frontal view **C** labrum, dorsal view **D** T1–T3, dorsal view **E** mesosoma, dorsal view. Scale bar: 1.0 mm.

##### Paralectotypes

**(4 ♀).** 3 ♀, 25.IV.1871 // Чардара [Chardara] // к.[оллекция] Ф. Моравица [Collection of F. Morawitz] // Paralectotypus *Andr.semiaenea* Mor., design. Osychnjuk, 1980 <red label> [ZISP]; 1 ♀, 24.[IV.1871] // Косараль [Kosaral] // Paralectotypus *Andenasemiaenea* Mor., design. Osytshnjuk et al., 2008 <identical red labels on each paralectotype specimen, labelled Yu. Astafurova> [ZMMU].

##### Current status.

Andrena (Poecilandrena) semiaenea Morawitz, 1876.

##### Remarks.

Listed as *Lepidandrena* by Osytshnjuk et al. (2008: 155). Description of male: Gusenleitner and Schwarz 2001: 167.

##### Distribution.

Afghanistan, Uzbekistan, Kazakhstan, Mongolia.

#### ﻿43. Andrena smaragdina

Taxon classificationAnimaliaHymenopteraAndrenidae

Morawitz, 1876

1DE20C98-571A-51BA-9C30-4D65FD27CD34

Fig. 43

Andrena smaragdina Morawitz, 1876: 164, 165 (key), 211, ♀, ♂.

##### Type locality.

Samarkand (Uzbekistan).

##### Published (original) locality.

Uzbekistan: Samarkand.

##### Lectotype (designated here).

♀, Самаркандъ [Uzbekistan, Samarkand, 39°39'N, 66°57'E] // *Andrenasmaragdina* Mor. [handwritten by F. Morawitz] // к.[оллекция] Ф. Моравица [Collection of F. Morawitz] // Paralectotypus *Andrenasmaragdina* Mor., design. Osychnjuk, 1980 <red label> // Lectotypus *Andrenasmaragdina* Morawitz, 1876, design. Astafurova et al., 2022 <red label> // Zoological Institute St. Petersburg INS_HYM_0000293 [ZISP].

**Figure 43.**
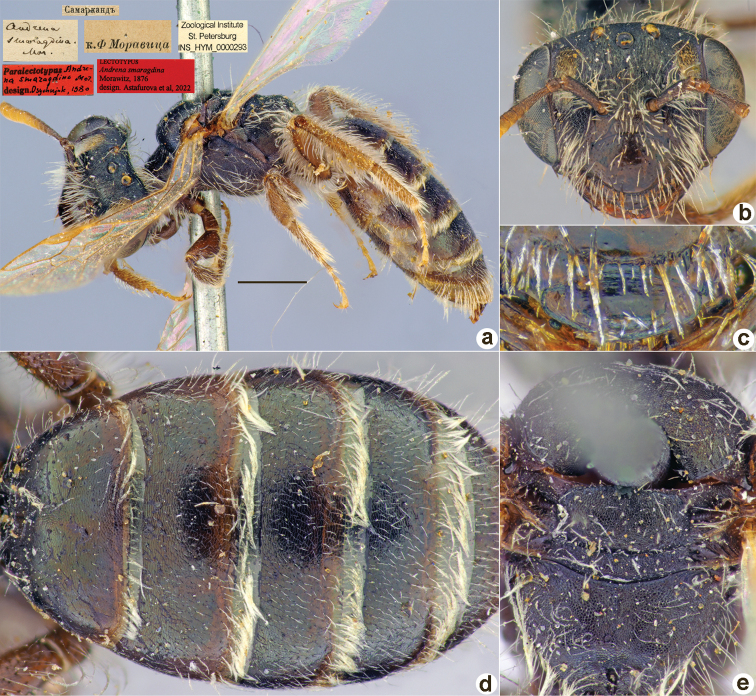
*Andrenasmaragdina* Morawitz, 1876, lectotype, female **A** habitus, lateral view and labels **B** head, frontal view **C** labrum, dorsal view **D** metasoma, dorsal view **E** mesosoma, dorsal view. Scale bar: 1.0 mm.

##### Paralectotypes

**(2 ♀, 1 ♂).** 1 ♀, 28.[II.1869] // Самаркандъ // *Andrenasmaragdina* Mor. [handwritten by F. Morawitz] // Lectotypus Warncke 1975 <red label>; 1 ♀, 1 ♂, 10., 28.[II.1869] // Самаркандъ // Paralectotypus *Andrenasmaragdina* Morawitz, 1876, design. Astafurova et al., 2022 <red label> [ZMMU].

##### Current status.

Andrena (Notandrena) smaragdina Morawitz, 1876.

##### Remarks.

Listed as *Poecilandrena* by Osytshnjuk et al. (2005: 28).

##### Distribution.

Uzbekistan, Tajikistan.

#### ﻿44. Andrena sogdiana

Taxon classificationAnimaliaHymenopteraAndrenidae

Morawitz, 1876

3BEFB11C-F0B3-50CF-BB78-B4EBFD4EF6E0

Fig. 44

Andrena sogdiana Morawitz, 1876: 165 (key), 177, ♂.

##### Type locality.

Samarkand (Uzbekistan).

##### Published (original) locality.

Uzbekistan: Samarkand.

##### Lectotype (designated here).

♂, 11.[IV.1869] // Самаркандъ [Uzbekistan, Samarkand, 39°39'N, 66°57'E] // *Andrenasogdiana* Mor. [handwritten by F. Morawitz] // Paralectotypus *Andrenasogdiana* Mor., design. Osychnjuk, 1980 <red label> // *Andrenaoralis*, ♂, D.A. Sidorov det. 2022 // Lectotypus *Andrenasogdiana* Morawitz, 1876, design. Astafurova et al., 2022 <red label> // Zoological Institute St. Petersburg INS_HYM_0000292 [ZISP].

**Figure 44.**
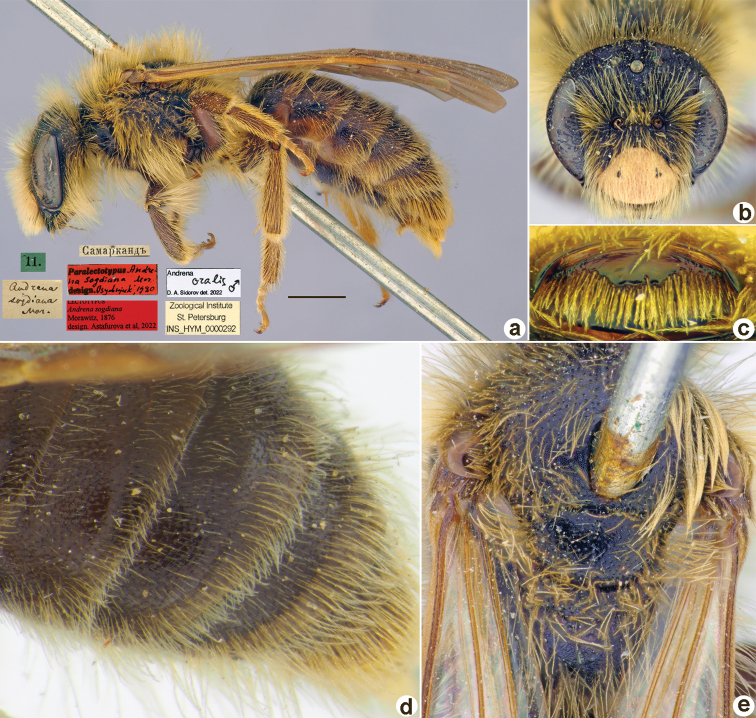
*Andrenasogdiana* Morawitz, 1876, lectotype, male **A** habitus, lateral view and labels **B** head, frontal view **C** labrum, dorsal view **D** metasoma, latero-dorsal view **E** mesosoma, dorsal view. Scale bar: 1.0 mm.

##### Current status.

Andrena (Orandrena) oralis Morawitz, 1876 (according to Gusenleitner and Schwarz 2001: 150).

##### Remarks.

There is a male specimen labelled by Warncke as a “Lectotypus” (3.[IV.1869] // Самарканд // *Andrenasogdiana* Mor.[handwritten by F. Morawitz] // Lectotypus Warncke 1975; ZMMU). However, the label date does not correspond the date [11.IV.1869] mentioned for the type series by Morawitz (1876).

##### Distribution.

Europe, Russia (to the Urals), Turkey, Turkmenistan, Uzbekistan, Kazakhstan.

#### ﻿45. Andrena sordida

Taxon classificationAnimaliaHymenopteraAndrenidae

Morawitz, 1876

1462616D-C19B-5A54-94EC-7FD24029765F

Fig. 45

Andrena sordida Morawitz, 1876: 163, 166 (key), 173, ♀, ♂.

##### Type locality.

Tashkent (Uzbekistan).

##### Published (original) locality.

Uzbekistan: near Tashkent.

##### Lectotype.

♀, designated by Osytshnjuk et al. 2008: 161, 5.[IV.1871] // Ташкентъ [Uzbekistan, Tashkent, 41°18'N, 69°16'E] // *Andrenasordida* Mor. [handwritten by F. Morawitz] // Lectotypus Warncke 1975 <red label> // Lectotypus *Andrenasordida* Morawitz, 1876, design. Osytshnjuk et al., 2008 // <red label, labelled by Yu. Astafurova> [ZMMU].

**Figure 45.**
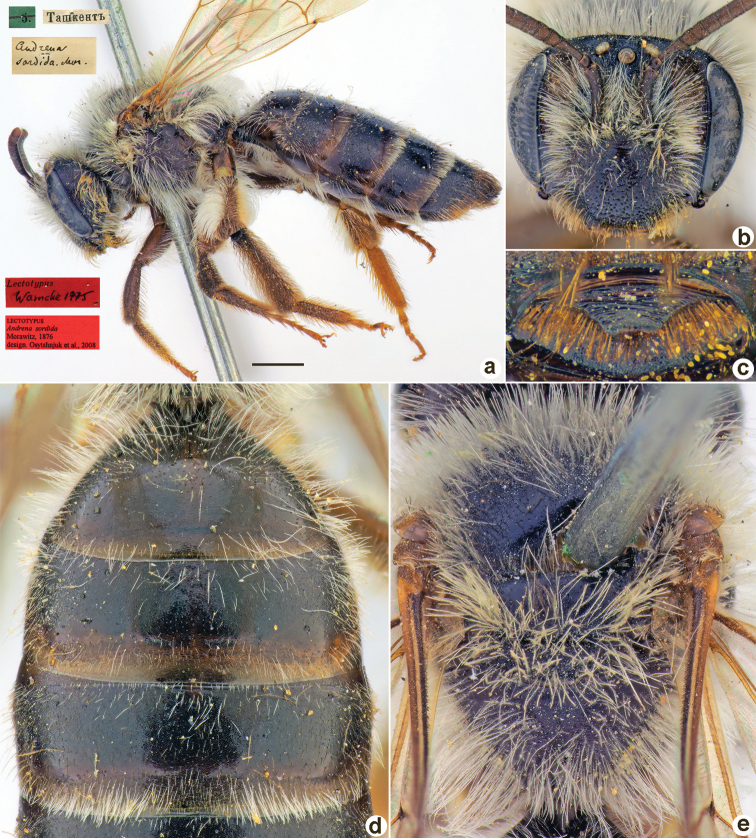
*Andrenasordida* Morawitz, 1876, lectotype, female **A** habitus, lateral view and labels **B** head, frontal view **C** labrum, dorsal view **D** T1–T3, dorsal view **E** mesosoma, dorsal view. Scale bar: 1.0 mm.

##### Paralectotypes

**(83 ♀, 2 ♂).** 1 ♀, 1 ♂, the same label as in the lectotype; 4 ♀, Ташкентъ // к.[оллекция] Ф. Моравица [Collection of F. Morawitz] // *sordida* [handwritten by F. Morawitz] // Paralectotypus *Andr.sordida* Mor., design. Osychnjuk, 1980 <identical red labels on each paralectotype specimen> [ZISP]; 78 ♀, 1 ♂, 1., 3., 5., 8.[IV.1871], 28.[III.1871] // Ташкентъ // Paralectotypus *Andrenasordida* Mor. design. Osytshnjuk et al., 2008 // <red label, labelled by Yu. Astafurova> [ZMMU].

##### Current status.

Andrena (Leucandrena) sordidella Viereck, 1918, replacement name for *A.sordida* Morawitz, 1876 (nec *Apissordida* Scopoli, 1763; nec *Apissordida* Gmelin, 1790).

##### Distribution.

Uzbekistan.

#### ﻿46. Andrena subaenescens

Taxon classificationAnimaliaHymenopteraAndrenidae

Morawitz, 1876

D0F5D5E8-812B-5682-9441-5276A1375D7D

Fig. 46

Andrena subaenescens Morawitz, 1876: 164, 166 (key) 207, ♀, ♂.

##### Type locality.

Samarkand (Uzbekistan).

##### Published (original) locality.

Uzbekistan: Samarkand.

##### Lectotype (designated here).

♀, <golden circle> // 27.[II.1869] // Самаркандъ [Uzbekistan, Samarkand, 39°39'N, 66°57'E] // *subaenescens* Mor. Typ. [handwritten by F. Morawitz] // Paralectotypus *Andrenasubaenescens* Mor., design. Osychnjuk, 1980 <red label> // Lectotypus *Andrenasubaenescens* Morawitz, 1876, design. Astafurova et al., 2022 <red label> // Zoological Institute St. Petersburg INS_HYM_0000255 [ZISP].

**Figure 46.**
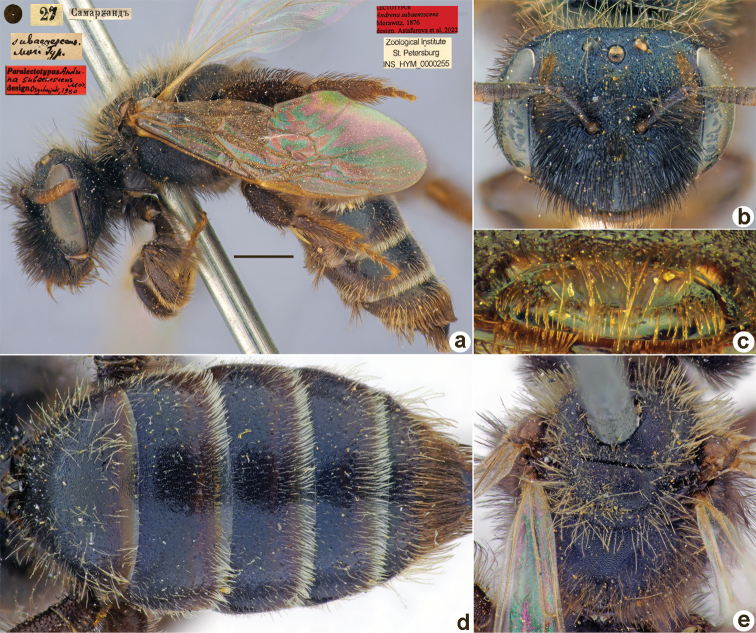
*Andrenasubaenescens* Morawitz, 1876, lectotype, female **A** habitus, lateral view and labels **B** head, frontal view **C** labrum, dorsal view **D** metasoma, dorsal view **E** mesosoma, dorsal view. Scale bar: 1.0 mm.

##### Paralectotypes

**(8 ♀, 1 ♂).** 3 ♀, 27.[II.1869] // Самаркандъ // к.[оллекция] Ф. Моравица [Collection of F. Morawitz] // Paralectotypus *Andr.sordida* Mor., design. Osychnjuk, 1980 <red label> [ZISP]; 1 ♀, 27.[II.1869] // Самаркандъ // *Andrenasubaenescens* Mor. [handwritten by F. Morawitz] // Lectotypus Warncke, 1975 <red label>; 4 ♀, 1 ♂. 27., 28.[II–18.III.1869] // Самаркандъ // Paralectotypus *Andrenasubaenescens* Morawitz, 1876, design. Astafurova et. al., 2022 <identical red labels on each paralectotype specimen> [ZMMU].

##### Current status.

Andrena (Poecilandrena) subaenescens Morawitz, 1876.

##### Distribution.

Uzbekistan.

#### ﻿47. Andrena temporalis

Taxon classificationAnimaliaHymenopteraAndrenidae

Morawitz, 1876

6C6D8114-958B-53F9-A23C-DC08B526346F

Fig. 47

Andrena temporalis Morawitz, 1876: 165 (key), 204, ♂.

##### Type locality.

Samarkand (Uzbekistan).

##### Published (original) locality.

Uzbekistan: Samarkand.

##### Lectotype.

♂, designated by Osytshnjuk et al. 2005: 151, 20.[III.1869] // Самаркандъ [Uzbekistan, Samarkand, 39°39'N, 66°57'E] // *Andrenatemporalis* Mor. [handwritten by F. Morawitz] // Lectotypus Warncke 1975 <red label> // Lectotypus, *Andrenatemporalis* Morawitz, 1876, design. Osytshnjuk et al., 2008 // <red label, labelled by Yu. Astafurova> [ZMMU].

**Figure 47.**
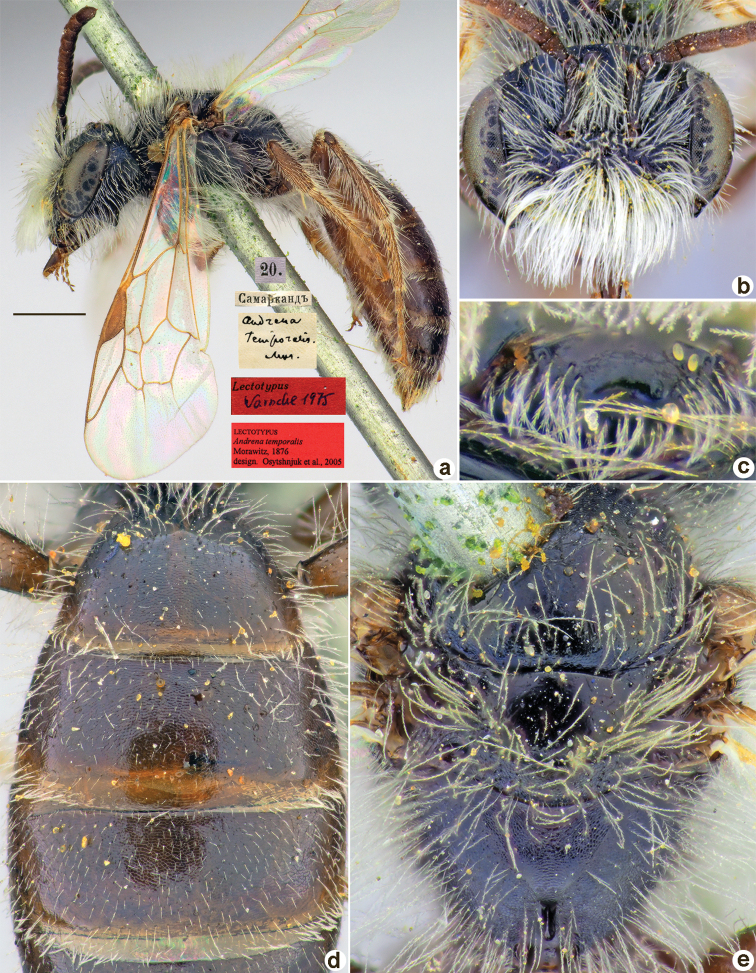
*Andrenatemporalis* Morawitz, 1876, lectotype, male **A** habitus, lateral view and labels **B** head, frontal view **C** labrum, dorsal view **D** T1–T3, dorsal view **E** mesosoma, dorsal view. Scale bar: 1.0 mm.

##### Paralectotypes

**(7 ♂).** 1 ♂, <golden circle> // 20.[III.1869] // Самаркандъ // *temporalis* Mor. Typ. [handwritten by F. Morawitz]; 1 ♂, Самаркандъ // к.[оллекция] Ф. Моравица [Collection of F. Morawitz] // *Andrenatemporalis* F. Morawitz, ♂ [handwritten by F. Morawitz] // Paralectotypus *Andr.temporalis* Mor., design. Osychnjuk, 1980 <red label> [ZISP]; 5 ♂, 16., 20.[III.1869] // Самаркандъ // Paralectotypus, *Andrenatemporalis* Morawitz, 1876, design. Osytshnjuk et al., 2005 et al. <identical red labels on each paralectotype specimen, labelled by Yu. Astafurova> [ZMMU].

##### Current status.

Andrena (Notandrena) hieroglyphica Morawitz, 1876 (synonymised by Osytshnjuk 1984: 3).

##### Distribution.

Iran, Turkmenistan, Uzbekistan, Tajikistan, Pakistan.

#### ﻿48. Andrena tuberculiventris

Taxon classificationAnimaliaHymenopteraAndrenidae

Morawitz, 1876

24AED2F6-5BDF-5506-83FF-44B1EA1B7476

Fig. 48

Andrena tuberculiventris Morawitz, 1876: 166 (key), 184, ♂.

##### Type locality.

Tashkent (Uzbekistan).

##### Published (original) locality.

Uzbekistan: Tashkent.

##### Lectotype (designated here).

♂, 10.[IV.1871] // Ташкентъ [Uzbekistan, Tashkent, 41°18'N, 69°16'E] // *Andrenatuberculiventris* Mor. [handwritten by F. Morawitz] // Lectotypus Warncke 1975 <red label> // Lectotypus *Andrenatuberculiventris* Morawitz, 1876, design. Astafurova et al., 2022 <red label> [ZMMU].

**Figure 48.**
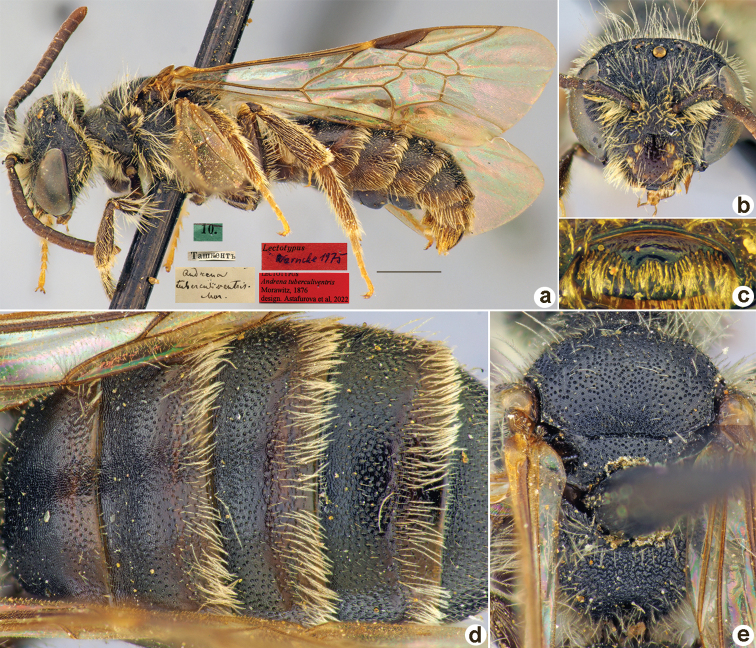
*Andrenatuberculiventris* Morawitz, 1876, lectotype, male **A** habitus, lateral view and labels **B** head, frontal view **C** labrum, dorsal view **D** metasoma, dorsal view **E** mesosoma, dorsal view. Scale bar: 1.0 mm.

##### Paralectotypes

**(3 ♂).** 1 ♂, <golden circle> // 10.[IV.1871] // Ташкентъ // *tuberculiventris* Mor. Typ. [handwritten by F. Morawitz] // Paralectotypus *Andr.tuberculiventris* Mor., design. Osychnjuk, 1980 <red label> [ZISP]; 2 ♂, 10.[IV.1871] // Ташкентъ // Paralectotypus *Andrenatuberculiventris* Morawitz, 1876, design. Astafurova et al., 2022 <red label> [ZMMU].

##### Current status.

Andrena (Parandrenella) tuberculiventris Morawitz, 1876 (according to Scheuchl et al. 2011: 225).

##### Remarks.

Listed as Andrena (Parandrenella) bicarinata Morawitz, 1876 by Gusenleitner and Schwarz (2002: 123).

##### Distribution.

Uzbekistan, Tajikistan.

#### ﻿49. Andrena turkestanica

Taxon classificationAnimaliaHymenopteraAndrenidae

Morawitz, 1876

D1059395-B44F-5680-B205-0F4EA578CEE0

Fig. 49

Andrena turkestanica Morawitz, 1876: 162, 166 (key), 192, ♀, ♂.

##### Type locality.

Urgut, Zeravshan River valley (Uzbekistan).

##### Published (original) locality.

Uzbekistan: Zeravshan River valley; steppe between Ulus and Dzham.

##### Lectotype (designated here).

♀, Верхн.[ий] Заравш.[ан] [Uzbekistan, Zeravshan River valley, near Urgut, 39°24'N, 67°13'E] // *turkestanica* F. Mor. ♀, [handwritten by F. Morawitz] // к.[оллекция] Ф. Моравица [Collection of F. Morawitz] // Paralectotypus *Andr.turkestanica* Mor., design. Osychnjuk, 1980 <red label> // Lectotypus *Andrenaturkestanica* Morawitz, 1876, design. Astafurova et al., 2022 <red label> // Zoological Institute St. Petersburg INS_HYM_0000285 [ZISP].

**Figure 49.**
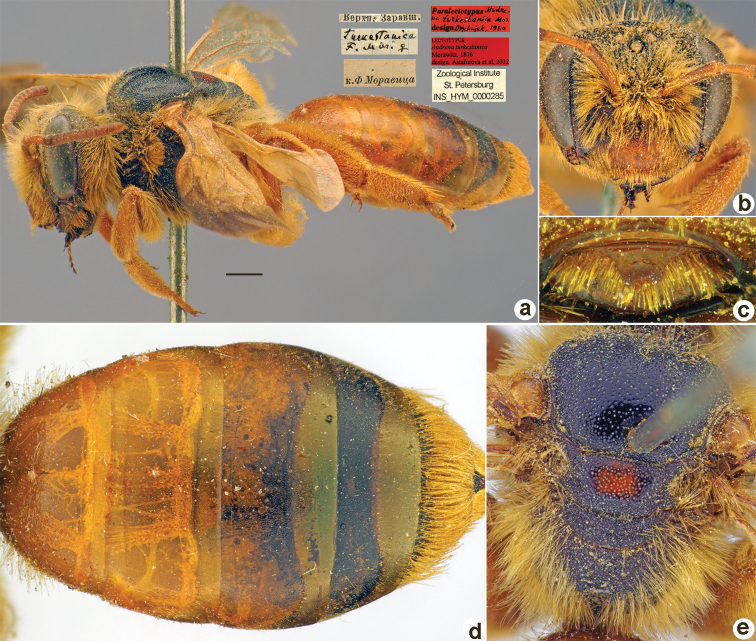
*Andrenaturkestanica* Morawitz, 1876, lectotype, female **A** habitus, lateral view and labels **B** head, frontal view **C** labrum, dorsal view **D** metasoma, dorsal view **E** mesosoma, dorsal view. Scale bar: 1.0 mm.

##### Paralectotypes

**(3 ♀).** 2 ♀, <golden circle> // Верхн. Заравш. // *turkestanica* Mor. Typ. ♀ [handwritten by F. Morawitz] // Paralectotypus *Andr.turkestanica* Mor., design. Osychnjuk, 1980 <red label> [ZISP]; 1 ♀, 12.[V.1869] // Верхн. Заравш. // *Andrenaturkestanica* Mor. // Lectotypus Warncke 1975 <red label> // Paralectotypus *Andrenaturkestanica* Morawitz, 1876, design. Astafurova et al., 2022 <identical red labels on each paralectotype specimen> [ZMMU].

##### Current status.

Andrena (Melanapis) fuscosa Erichson, 1835 (synonymised by Warncke 1967: 315).

##### Remarks.

Listed as “*turcestanica*” by Gusenleitner and Schwarz (2002: 303).

##### Distribution.

Europe, North Africa, Russia (Dagestan Rep.), Caucasus, Turkey, Syria, Lebanon, Israel, Jordan, Iran, Afghanistan, Pakistan, Central Asia, India.

#### ﻿50. Andrena urmitana

Taxon classificationAnimaliaHymenopteraAndrenidae

Morawitz, 1876

EFE42609-8123-5324-8A1B-58479B7E0AD7

Fig. 50

Andrena urmitana Morawitz, 1876: 165 (key), 175, ♂.

##### Type locality.

Urmetan (Tajikistan).

##### Published (original) locality.

Tajikistan: near Urmitan [Urmetan].

##### Holotype.

♂, 2.[V.1869] // Урмитанъ [Tajikistan, Urmetan, 39°26'N, 68°15'E] // *Andrenaurmitana* Mor. [handwritten by F. Morawitz] // Lectotypus Warncke 1975 <red label> // Holotypus *Andrenaurmitana* Mor., 1876 <red label> [ZMMU].

**Figure 50.**
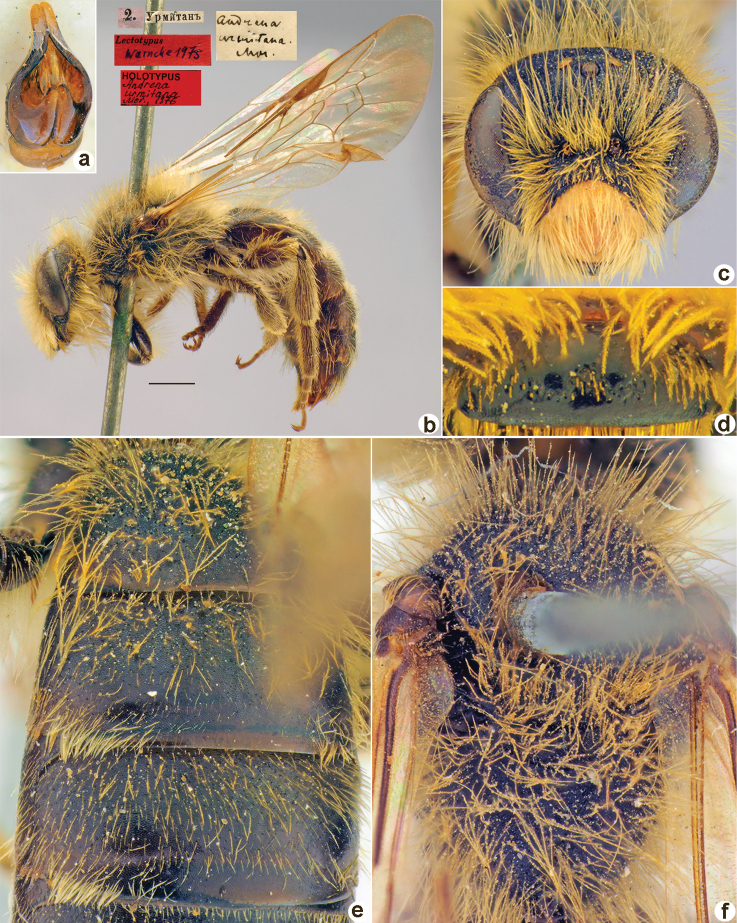
*Andrenaurmitana* Morawitz, 1876, lectotype, male **A** genitalia, dorsal view **B** habitus, lateral view and labels **C** head, frontal view **D** labrum, dorsal view **E** T1–T3, dorsal view **F** mesosoma, dorsal view. Scale bar: 1.0 mm.

##### Current status.

Andrena (Nobandrena) acutilabris Morawitz, 1876 (according to Gusenleitner and Schwarz 2002: 410).

##### Distribution.

Turkmenistan, Uzbekistan, Tajikistan, Kazakhstan.

#### ﻿51. Andrena virescens

Taxon classificationAnimaliaHymenopteraAndrenidae

Morawitz, 1876

3B63BDF3-4103-57A6-84AF-18ABE16ECA87

Fig. 51

Andrena virescens Morawitz, 1876: 164, 165 (key), 209, ♀, ♂.

##### Type locality.

Tashkent (Uzbekistan).

##### Published (original) locality.

Uzbekistan: near Samarkand, near Tashkent, between Tashkent and Keless River.

##### Lectotype (designated here).

♀, Ташкентъ [Uzbekistan, Tashkent, 41°18'N, 69°16'E] // *virescens* Mor., ♀ [handwritten by F. Morawitz] // к.[оллекция] Ф. Моравица [Collection of F. Morawitz] // Paralectotypus *Andr.virescens* Mor., design. Osychnjuk, 1980 <red label> // Lectotypus *Andrenavirescens* Morawitz, 1876, design. Astafurova et al., 2022 <red label> // Zoological Institute St. Petersburg INS_HYM_0000254 [ZISP].

**Figure 51.**
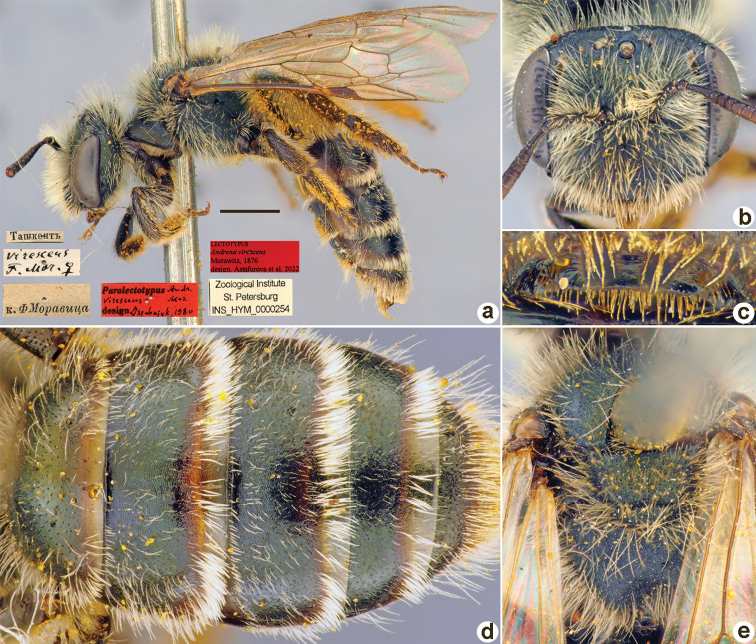
*Andrenavirescens* Morawitz, 1876, lectotype, female **A** habitus, lateral view and labels **B** head, frontal view **C** labrum, dorsal view **D** metasoma, dorsal view **E** mesosoma, dorsal view. Scale bar: 1.0 mm.

##### Paralectotypes

**(7 ♀, 8 ♂).** 4 ♂, 27., 28.[II.1869] // Самаркандъ // к.[оллекция] Ф. Моравица [Collection of F. Morawitz] // Paralectotypus *Andr.virescens* Mor., design. Osychnjuk, 1980 <red label> [ZISP]; 1 ♂, 27.[II.1869] // Самаркандъ // *Andrenavirescens* Mor. [handwritten by F. Morawitz] // Lectotypus Warncke, 1975 <red label>; 5 ♀, 1 ♂, 10., 23., 24.[III.1871], 8.[IV.1871] // Ташкентъ; 1 ♀, 2 ♂, 27.[II.1869] // Самаркандъ ; 1 ♀, 22.[IV.1871] // Келесъ // // Paralectotypus, *Andrenavirescens* Morawitz, 1876, design. Astafurova et al., 2022 <identical red labels on each paralectotype specimen> [ZMMU].

##### Current status.

Andrena (Poecilandrena) virescens Morawitz, 1876.

##### Distribution.

Uzbekistan, Tajikistan.

#### ﻿52. Andrena viridigastra

Taxon classificationAnimaliaHymenopteraAndrenidae

Morawitz, 1876

ACA99AF2-42E2-566A-AEAA-5EDD94580840

Fig. 52

Andrena viridigastra Morawitz, 1876: 164, 165 (key), 206, ♀, ♂.

##### Type locality.

Tashkent (Uzbekistan).

##### Published (original) locality.

Uzbekistan: Tashkent, Samarkand.

##### Lectotype.

♀, designated by Osytshnjuk et al. 2008: 220, 28.[II.1871] // Ташкентъ [Uzbekistan, Tashkent, 41°18'N, 69°16'E] // *Andrenaviridigastra* Mor. [handwritten by F. Morawitz] // Lectotypus Warncke 1975 <red label> // Lectotypus, *Andrenaviridigastra* Morawitz, 1876, design. Osytshnjuk et al., 2008 // <red label, labelled by Yu. Astafurova> [ZMMU].

**Figure 52.**
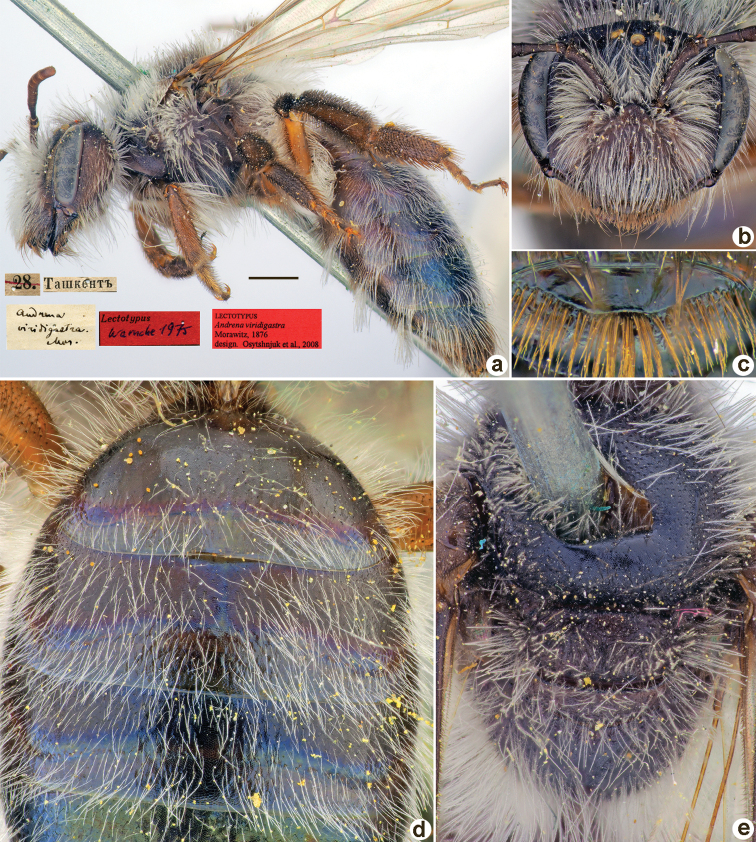
*Andrenaviridigastra* Morawitz, 1876, lectotype, female **A** habitus, lateral view and labels **B** head, frontal view **C** labrum, dorsal view **D** T1–T3, dorsal view **E** mesosoma, dorsal view. Scale bar: 1.0 mm.

##### Paralectotypes

**(29 ♀, 32 ♂).** 1 ♀, <golden circle>, 16.[III.1869] // Самаркандъ // *viridigastra* Mor., ♀, Typ. [handwritten by F. Morawitz]; 2 ♀, 27.[II.1871] // Ташкентъ [Tashkent] // к.[оллекция] Ф. Моравица [Collection of F. Morawitz] // *viridigastra* Mor. [handwritten by F. Morawitz]; 1 ♂, <golden circle> // 16.[III.1871] // Ташкентъ // *viridigastra* Mor. ♂, Typ. [handwritten by F. Morawitz] // Paralectotypus *Andr.viridigastra* Mor., design. Osychnjuk, 1980 <identical red labels on each paralectotype specimen> [ZISP]; 20 ♀, 14 ♂, 28.[II.1871], 16., 23., 24., 25., 27.[III.1871], 5., 8., 10., 11.[IV.1871] // Ташкентъ; 6 ♀, 17 ♂, 16., 18., 21., 25[III.1869] // Самаркандъ // Paralectotypus, *Andrenaviridigastra* Morawitz, 1876, design. Osytshnjuk et al., 2005 <identical red labels on each paralectotype specimen, labelled by Yu. Astafurova> [ZMMU].

##### Current status.

Andrena (Melandrena) viridigastra Morawitz, 1876.

##### Distribution.

Turkmenistan, Uzbekistan, Tajikistan, Kazakhstan.

## Supplementary Material

XML Treatment for Andrena acutilabris

XML Treatment for Andrena ahenea

XML Treatment for Andrena amoena

XML Treatment for Andrena arenaria

XML Treatment for Andrena aulica

XML Treatment for Andrena bairacumensis

XML Treatment for Andrena bicarinata

XML Treatment for Andrena capillosa

XML Treatment for Andrena carinifrons

XML Treatment for Andrena combusta

XML Treatment for Andrena comparata

XML Treatment for Andrena corallina

XML Treatment for Andrena discophora

XML Treatment for Andrena fedtschenkoi

XML Treatment for Andrena ferghanica

XML Treatment for Andrena flavitarsis

XML Treatment for Andrena fuscicollis

XML Treatment for Andrena hieroglyphica

XML Treatment for Andrena infirma

XML Treatment for Andrena initialis

XML Treatment for Andrena laeviventris

XML Treatment for Andrena lateralis

XML Treatment for Andrena leucorhina

XML Treatment for Andrena lucidicollis

XML Treatment for Andrena maculipes

XML Treatment for Andrena majalis

XML Treatment for Andrena mordax

XML Treatment for Andrena mucorea

XML Treatment for Andrena nigrita

XML Treatment for Andrena nitidicollis

XML Treatment for Andrena nupta

XML Treatment for Andrena oralis

XML Treatment for Andrena pannosa

XML Treatment for Andrena planirostris

XML Treatment for Andrena punctifrons

XML Treatment for Andrena punctiventris

XML Treatment for Andrena quadrifasciata

XML Treatment for Andrena ravicollis

XML Treatment for Andrena rufilabris

XML Treatment for Andrena rufina

XML Treatment for Andrena sarta

XML Treatment for Andrena semiaenea

XML Treatment for Andrena smaragdina

XML Treatment for Andrena sogdiana

XML Treatment for Andrena sordida

XML Treatment for Andrena subaenescens

XML Treatment for Andrena temporalis

XML Treatment for Andrena tuberculiventris

XML Treatment for Andrena turkestanica

XML Treatment for Andrena urmitana

XML Treatment for Andrena virescens

XML Treatment for Andrena viridigastra
